# Computational advances in the design and discovery of artemis inhibitors for radiosensitization in cancer therapy

**DOI:** 10.3389/fchem.2025.1597454

**Published:** 2025-07-28

**Authors:** Maryam Bashir, Usman Abdullah, Sadia Nazir, Farhan Siddique, Nasir Jalal

**Affiliations:** ^1^ Department of Pharmaceutical Chemistry, Faculty of Pharmacy, Bahauddin Zakariya University, Multan, Pakistan; ^2^ Department of Biomedical Sciences, Pak-Austria Fachhochschule, Haripur, Pakistan; ^3^ Atta ur Rahman School of Applied Biosciences, National University of Sciences and Technology, Islamabad, Pakistan; ^4^ Baiao Kuntai Biotechnology, China-Europe Innovation Center, Hangzhou, Zhejiang, China

**Keywords:** ARTEMIS, radiosensitizers, DNA repair mechanism, DFT, MM-GBSA, free energy landscape

## Abstract

**Introduction:**

Artemis is a key scaffold repair protein involved in the non-homologous end-joining (NHEJ) DNA repair pathway and is encoded by the DCLRE1C gene in humans. Its inhibition disrupts double-strand break (DSB) repair, sensitizing cancer cells to ionizing radiation (IR). However, no Artemis-targeted inhibitors are currently available for therapeutic use. This study aims to identify and characterize novel small-molecule Artemis inhibitors that act as potential radiosensitizers in cancer treatment.

**Methods:**

Micronuclei formation was assessed in Artemis-deficient (CJ179), proficient (1BR3), and mutant (48BR) cell lines following 1 Gy IR exposure. Initial in vitro screening identified HMAD as a potential Artemis inhibitor. A focused virtual screening of 69 compounds was performed using AutoDock4 and Glide to evaluate binding affinity to Artemis. The top 16 compounds (ΔG < −8.0 kcal/mol) were further analyzed. Density Functional Theory (DFT) calculations at the B3LYP/6−311+G(d,p) level were used to assess frontier molecular orbitals and reactivity. ADMET profiling was conducted to evaluate pharmacokinetic properties. Compounds 42 and 51 were subjected to 100 ns molecular dynamics (MD) simulations with MMGBSA binding free energy calculations, PCA, and FEL analysis.

**Results:**

CJ179 cells exhibited significantly higher micronuclei post-irradiation, confirming Artemis’s role in DNA repair. Among the top hits, compound 42 showed a highly stable binding profile, with a favorable MMGBSA binding energy of −36.94 kcal/mol. ADMET analysis indicated optimal drug-like properties. MD simulations revealed stable interaction trajectories, hydrogen bonding, and a narrow binding pocket. PCA and FEL analysis further supported the dynamic stability of compound 42.

**Discussion:**

This study identifies compound 42 as a promising Artemis inhibitor with potential as a radiosensitizing agent. The integrated *in vitro* and computational findings offer a foundation for further preclinical development, contributing to more effective radiotherapy strategies in cancer treatment.

## Introduction

Radiotherapy has been established as a highly effective treatment strategy for tumors. The ionizing radiation, used for radiotherapeutic purposes, relies on generating DNA double-strand breaks (DSB) in the target cancerous tissue (van de Kamp et al., 2021) that leads to eventual cell death. Radiosensitizers are believed to increase the therapeutic ratio by inhibiting the repair protein activation of DNA-PKcs, XRCC-ligase IV, and Artemis, which are involved in the DNA repair mechanism (van de Kamp et al., 2021). Several chemical inhibitors of DNA-dependent protein kinase catalytic subunit (DNA-PKcs), specifically NU7026 and AZD7648, have been used in versatile experimental scenarios as radiosensitizers (GLORIEUX, 2020). Recently, Artemis inhibition has been reported as a therapeutic strategy for acute lymphoblastic leukemia (Watanabe et al., 2022; Liu et al., 2018; Li et al., 2014) that effectively halted the DNA repair mechanism in irradiated cells, with negligible clinical consequences on normal cells (Watanabe et al., 2022). The small interfering RNA (siRNA) mediated Artemis inhibition increases the radiosensitivity of target colorectal cell line (RKO) cells *in vitro* (Liu et al., 2018). Small organic compounds such as ampicillin, ceftriaxone, ebselen, disulfiram, and auranofin hindered Artemis functioning with modest IC_50_ values (Li et al., 2014; Yosaatmadja et al., 2021), which has caught the attention of researchers who are seeking to identify small molecules as Artemis inhibitors in the development of radiosensitizers.

As part of the DNA double-strand break repair (DSBR) cascade, Artemis is phosphorylated for repair activation at S516 and S645 in unirradiated cells. Following treatment with DSB-inducing drug bleomycin, this phosphorylation is enhanced 10-15-fold (Soubeyrand et al., 2006). In addition to being a phosphorylation target of Ataxia-Telangiectasia Mutated (ATM) and DNA-PKcs, Artemis can also be phosphorylated by the Ataxia-Telangiectasia and Rad3-Related (ATR) kinase. However, it interacts with known cell cycle checkpoint proteins and becomes a target of ATM or ATR after exposure to ionizing radiation (IR) or ultraviolet (UV) radiation, respectively (Zhang et al., 2004). DSBs are among the most lethal forms of DNA damage, and their efficient repair is essential for the survival of cancer cells. One of the primary mechanisms for repairing these DSBs in mammalian cells is the non-homologous end joining (NHEJ) pathway, which is active during the G1 phase of the cell cycle. The NHEJ pathway is largely mediated by a complex of proteins, including DNA-PK, Ku70/80, and Artemis. Artemis plays a critical role in processing DNA ends to accelerate ligation during NHEJ, making it a needed component of the DNA repair machinery. DNA PK-dependent phosphorylation of Artemis after treatment with DSB-inducing agents increased the cellular retention of Artemis, maintained its interaction with DNA at DSBs, and also activated its endonucleolytic activity (Ma et al., 2005). While NHEJ is critical for maintaining genomic stability, it also contributes to the survival of cancer cells after radiation-induced damage. As such, targeting Artemis and other components of the NHEJ pathway has emerged as a promising strategy to enhance the efficacy of radiotherapy. Inhibiting Artemis could impair DNA repair, rendering cancer cells more susceptible to the lethal effects of radiation. This concept of radiosensitization, achieved through the inhibition of DNA repair pathways, could potentially improve treatment outcomes and overcome radioresistance in tumors that are refractory to radiation.

Artemis is a small (78 kDa) protein, a member of the metallo-β-lactamase family. It has both endonuclease and intrinsic 5′-exonuclease activity, while point mutation of the putative active site residue (H115A) can markedly reduce both endo and exonuclease activities. But it can be blocked by small yet specific molecule inhibitors (Li et al., 2014). The protein plays a role in the non-homologous end-joining pathway of DNA repair through its nuclease activity, facilitating the double-strand end processing following ionizing radiation exposure (Riballo et al., 2004). However, identifying and developing specific Artemis inhibitors remains a significant challenge. Traditional drug discovery approaches are time-consuming and expensive, making it essential to utilize computational methods to accelerate the identification of potential inhibitors.

Virtual high-throughput (vHTS) (Prieto-M et al., 2019; Aziz et al., 2022a) screening represented one of the most straightforward applications in drug design (Schmidtke and Barril, 2010; Agoni et al., 2020), employing a molecular docking program to determine how an entire database of existing or virtual compounds will bind to a specific target protein (Zoete et al., 2009). The commonly used docking programs, such as DOCK (Allen et al., 2015), AutoDock (Huey et al., 2007), Glide (Friesner et al., 2004), FlexX (Cross, 2005), GOLD (Verdonk et al., 2003), Surflex-Dock, MOE-Dock, and UCSF DOCK (Pagadala et al., 2017) rely on sampling algorithms (Huang and Zou, 2010) along with scoring functions (Schulz-Gasch and Stahl, 2004) to evaluate molecular interactions. This approach accelerated the drug discovery process by narrowing down vast libraries to a manageable number of promising candidates for detailed experimental testing (Zoete et al., 2009; Anwar et al., 2021). Artificial intelligence (AI) further aids in drug screening by predicting key physicochemical properties, influencing pharmacokinetics, and receptor target specificity (Zang et al., 2017). AI web-based tools like LimTox, SwissADME, admetSAR, ADMETlab3.0, Toxtree, and pkCSM can be used to predict physicochemical properties, thereby effectively reducing costs in drug development (Yang et al., 2019). Density Functional Theory (DFT)-based computations played a pivotal role by providing insights into the electronic properties (Tandon et al., 2019; Arshad et al., 2021), which are fundamental to understanding key molecular interactions, including covalent bonding, dipole-dipole and ion-dipole interactions, hydrogen bonding, hydrophobic effects, and charge transfer mechanisms. Such detailed analysis is crucial for elucidating the interaction dynamics between drug-like molecules and their biological targets, thereby enhancing therapeutics’ rational design and optimization (Reimers et al., 2003; Tuma et al., 1999; Ejaz et al., 2023). Furthermore, Molecular Dynamics (MD)-based binding free energy calculations help prioritize potential drug candidates during the hit identification phase (Li et al., 2019; Abel et al., 2017; Aziz et al., 2023). This approach provides a comprehensive understanding of atomic-level interactions and the dynamic behavior of biomolecular systems (Badar et al., 2022). The combined use of Free Energy Landscapes (FEL) and Principal Component Analysis (PCA) has proven to be a highly effective method for comprehensively exploring the conformational landscape of a protein and identifying its representative substrates.

In this study, we reported the *in silico* studies of 69 small-molecule inhibitors of Artemis, taken from a library with ZINC ID 846591, which was previously developed in the Sanford-Burnham Center for Chemical Genomics (the University of Southern California, Los Angeles, CA). The data was provided to the NIH Molecular Libraries Probe Production Network (https://www.broadinstitute.org/mlpcn/). Initially, virtual high-throughput screening (vHTS) was carried out using AutoDock and Glide docking programs, resulting in the identification of 16 hit compounds with high binding affinities. These compounds were further evaluated through *in silico* ADMET profiling using ADMETlab 3.0 (Valencia et al., 2023), followed by density functional theory (DFT) analysis. Based on favorable physicochemical properties and DFT results, compounds 42 and 51 were selected for MD simulations, FEL, and PCA analysis to assess their stability and interaction dynamics. The current study aims to find an Artemis inhibitor as a potential radiosensitizer adjuvant to cancer chemotherapy by employing the above computational techniques. According to the best of our knowledge, no experimental data have yet been defined on the role of an Artemis inhibitor as a radiosensitizer by *in vitro* or *in vivo* studies. This research can potentially lead to new therapeutic strategies targeting DNA repair mechanisms, offering new hope for patients with radioresistant cancers.

## Experimental methodology

### Sampling and processing of primary ductal carcinoma cells

Five Samples of invasive ductal carcinoma were collected from patients with grade 3 breast cancer after consent from the patients and surgeons from 4 hospitals in Rawalpindi/Islamabad. Samples were named as 1MOS, 3PRF, 2PRF, 1PTN, and 2PTN and collected in ice-cold Roswell Park Memorial Institute (RPMI) 1,640 (Life Technologies^®^ Cat No: 91800-014) supplemented with 10% horse serum (HS). And were processed as per the protocol of Potdar and Chaugule (Potdar et al., 2011) however, the protocol was modified in the lab according to the requirement. Samples were washed twice with 1X PBS. Tissues of 2 mm^2^ around the blood vessels were taken and fed in 10 mL RPMI+10 %HS and 1% Penicillin/Streptomycin (PenStrep) at 37°C and 5% CO_2_. Tissues were checked for logarithmic growth of cells for over a week.

### Primary cell culture

Four cancerous tissues obtained after surgical resection, with patients’ consent (from Jinnah hospital, Islamabad) were washed with ice cold 1XPBS three times; cut in small pieces of 5 × 5 mm and incubated in 4 mL of 0.25% trypsin EDTA (Gibco by Life technologies, reference # 25200-056) for 15 min followed by addition of 1XPBS 10 mL. The tissue pieces were chopped down further and were collected along with 1XPBS in a 15 mL tube and were spun at 1,000 rpm for 5 min. The supernatant and tissue debris were discarded. The cells were collected in the form of a pellet and were suspended in cell culture complete medium (RMPI 1640 medium and supplemented with 10% Horse Serum). Cells were cultured at 37°C and 5% CO_2_ in a humidified incubator in a sterile environment. After 5 days of development of primary culture, the cells were serum-starved. Serum starvation was done by changing the concentration of serum from 10% to 8% for 5 days and from 8% to 6% for the next 5 days, from 6% to 4% for the next 5 days, and then finally, 2% serum was provided. The idea behind the treatment was that the cancerous cell growth was independent of growth factor availability. Viable cells were used for culturing at each step at 1.0 x 10^5^ cells/mL of complete cell culture medium.

### Cell culture of artemis cell lines

The cell lines (CJ179, 1BR3, and 48BR) were obtained from Penelope Jeggo’s lab at the Genome Damage and Stability Centre, University of Sussex, United Kingdom. The Artemis-defective fibroblast CJ179 primary cell line was cultured in DMEM, supplemented with 10% FBS at 37°C with 5% CO_2_ (Krempler, 2004). Artemis proficient cell lines 1BR3 and 48BR were cultured by culture conditions mentioned by the United Kingdom Health Security Agency (https://www.culturecollections.org.uk/nop/product/1br3): briefly, we used DMEM + 2 mM L-glutamine +100 units/mL Penicillin, 100 μg/mL streptomycin, 0.4 μg.mL puromycin. Cells were seeded at 5 × 10,000 cells/cm^2^, using 0.05% trypsin/EDTA for splitting. The Artemis mutant (ATR-proficient) fibroblasts 48BR were cultured in DMEM, supplemented with 15% FBS (Burdak-Rothkamm et al., 2008).

### Micronuclei (MN) quantification

We exposed Artemis deficient (CJ179), proficient (1BR3), and mutated (48BR) cell lines to 1Gy of ionizing radiation and fixed them in methanol acetic acid (3:1 ratio), and spread them onto cold, dried glass slides. The cells were observed under ×100 magnification using oil immersion (Podrimaj-Bytyqi et al., 2018). The cells that were observed under the randomly selected field of view were analyzed for counting micronuclei.

### siRNA inhibition of artemis

Inhibition of Artemis was done at the transcription level by introducing siRNA into the invasive ductal carcinoma cell lines. Lipofectamine ™ 2000(Cat No 1668-027) was purchased from Invitrogen, and siRNA with sequence 5′-UUA​GGA​GUC​CAG​GUU​CAU​G-3′ (Zhang*,* et al., 2004) was purchased in duplex from Eurofins Operon. Two days before transfection, cells were grown in DMEM complete growth medium (Biowest: Cat No S181H-100) supplemented with 10% FBS. Lipofectamine ™ 2000 (3ul) was mixed with 150 µL of DMEM (without FBS) and left at room temperature for 5 min. After 5 min, 20 µL of 20 pmol/μL siRNA stock was added to the lipofectamine solution and incubated at room temperature for 20 min. The complex of siRNA and lipofectamine was added to 1 mL of cell suspension with 60%-70% confluence and incubated for 4 h in cell culture conditions. Then warm 1.5 mL of DMEM +10% FBS was added, and cells were incubated for 48-96 h.

### RNA extraction and cDNA synthesis

RNA was extracted by Trizol method (Sambrook et al., 1989) and was checked on 1% agarose gel by looking for two bands of 28S and 18S. For semi-quantitative analysis of gene expression cDNA was prepared from chemicals purchased from Thermoscientific. Oligo dt (2.5 µL), 5 µL Diethylpyrocarbonate water (DEPC), and RNA (17 µL) were mixed to make 25 µL of total volume, and PCR tubes were incubated at 70^o^C for 10 min and then chilled on ice for 5 min. Reverse Transcriptase (RT) buffer (10 µL), dNTPs (2.5 µL), DEPC water (8.75 µL), RNase inhibitor (1.25 µL), and RT enzyme (2.5 µL) were added to tubes and PCR was run for 1 h at 42°C. Tubes were stored at −20°C.

### Semi-quantitative analysis of gene expression

PCR of p53, DNA-PKcs, and Artemis Primer sequence and annealing temperatures (Table 1) was performed (Sambrook et al., 1989). These PCR products were then run on a 2% agarose gel as the product lengths were 121, 280, and 260 base pairs for p53, Artemis and DNA-PKcs, respectively. Bands were analyzed by the software ImageJ.

**TABLE 1:** qPCR primer sequences of p53, DNA-PKcs, and Artemis.

Serial no.	Gene	Sequence 5′→3′	Opt Tm- °C-PCR	References
1	Artemis-forward	GGA​CAA​GGG​TGG​TTG​GGA​GTA​GA	49.5	Zhang et al. (2009)
	Artemis-reverse	CCC​AAT​TGC​AGG​TAA​AAC​AGT​CAA​G	49.5	
2	DNA-PKcs-forward	CCG​GAC​GGA​CCT​ACT​ACG​ACT	57.1	Yasaei and Slijepcevic (2010)
	DNA-PKcs-reverse	AGA​ACG​ACC​TGG​GCA​TCC​T	57.1	
3	p53-forward	TAA​CAG​TTC​CTG​CAT​GGG​CGG​C	57	Węglarz et al. (2006)
	p53-reverse	AGG​ACA​GGC​ACA​AAC​ACG​CAC​C	57	

### Artemis inhibitor

The Artemis inhibitor used for *in vitro* experiments was (2-hydroxy-5-methoxybenzaldehyde 4-anilino-6-(3,5-dimethyl-H-pyrazol--yl)-,3,5-triazin-2-ylhydrazone, referred to as HMAD and purchased from the University of California San Diego.

### Chromosomal aberration assay

Chromosomal aberrations in Artemis-inhibited and exponentially growing cell lines were determined by metaphase spreads (Xiong et al., 2015). The cells were harvested by trypsinization (using 0.25% trypsin) 24 h after treatment and incubation with 1 μg of Colcemid/mL for 1 h to collect metaphase spreads for analysis. The cells were fixed after treatment with hypotonic solution (0.56% KCl) in Methanol-glacial acetic acid (3:1). Air-dried preparations were used, and slides were stained with 4′, 6-diamidino-2-phenylindole/Vectashield antifade mixture. For chromosomal aberrations, 25 mitotic cells were analyzed for each treatment per cell line at a magnification of ×100.

## Irradiation

a. Radiation of Artemis cell lines

The Artemis cell lines (CJ179, 1BR3, 48BR) were grown to 1 × 10^6^ cells/mL, irradiated with a^137^Cs source at a dose rate of 1.0 Gy/min, brought into the lab, and then evaluated for Micronucleus (MN) formation, and performed at Colorado State University, United States.

b. Radiation of Primary cells

Each sample of primary cells was split into 13 subgroups for irradiation. The first group was the absolute, which was not given any treatment. The next 4 groups (2-5) were incubated with NU7026 for 12 h before irradiation. The next 4 groups (6-9) were treated with artemis inhibitor for 12 h before irradiation. The rest of the 4 groups (10-13) were designated as control groups. Group 2 of each sample was irradiated with a radiation dose of 0.5 Gy, Group 3 of each sample was irradiated with a radiation dose of 1 Gy, Group 4 of each sample was irradiated with a radiation dose of 2 Gy and Group 5 of each sample was irradiated with a radiation dose of 4 Gy. The rest of the parameters were kept the same. Table 2 summarizes the treatment strategy for subgroups of sample 1. The rest of the subgroups from the three samples were treated accordingly. These irradiations utilized a Cobalt source and were performed at the Nuclear Medicine, Oncology Radiotherapy Institute (NORI) Hospital, Pakistan. A^60^Co source (Model: Theratron Phoenix) was used for irradiation of samples at the Nuclear Medicine, Oncology Radiotherapy Institute (NORI), Islamabad, Pakistan. The source diameter was 2 cm, and the source activity was 8,382 Ci.

**TABLE 2:** Inhibitor treatment strategies for primary cells coupled with irradiation dose.

Sample/Group	Radiation dose	Chemical inhibitor
Sample1/Group1	0.0 Gy	None
Sample1/Group2	0.5 Gy	DNA PK (inhibitor NU7026)
Sample1/Group3	1 Gy	DNA PK (inhibitor NU7026)
Sample1/Group4	2 Gy	DNA PK (inhibitor NU7026)
Sample1/Group5	4 Gy	DNA PK (inhibitor NU7026)
Sample1/Group6	0.5 Gy	Artemis inhibitor (HMAD)
Sample1/Group7	1 Gy	Artemis inhibitor (HMAD)
Sample1/Group8	2 Gy	Artemis inhibitor (HMAD)
Sample1/Group9	4 Gy	Artemis inhibitor (HMAD)
Sample1/Group10	0.5 Gy	None
Sample1/Group11	1 Gy	None
Sample1/Group12	2 Gy	None
Sample1/Group13	4 Gy	None

The different parameters calculated in dosimetry (Parallel Opposed Beam Pair Calculation Results) are given below in Table 3.

**TABLE 3:** Dosimetry calculations for irradiation of primary cells.

Parameter	^a^Calculated result	
Total dose	0.5 Gy/1Gy/2Gy/4Gy	
Fraction	0.5 Gy	
Field size (collimator opening)	Rt lateral	20 cmX28cm
	Lt lateral	20 cmX28cm
Depth of dose prescription point	Rt lateral	9 cm
	Lt lateral	9 cm
Dose fraction at Zmax	Rt lateral	0.28Gy
	Lt lateral	0.28Gy
Beam weight	50%	
Current beam output	1.848 Gy/min	

^a^ Parameters related to irradiation were calculated by a professional dosimetrist.

### Comet assay

Cells were incubated at standard conditions for the next 24 h after irradiation. Cells were then collected in a 15 mL tube after trypsinization. The cell suspension was then centrifuged at 1,000 rpm for 5 min to collect the cell pellet. The pellet was resuspended in 200 μL of 1XPBS and mixed with 1% Low Melting Point Agarose in a 1:10 ratio. Glass Slides were coated with 1% normal melting point agarose (molten) and allowed to dry on ice. The cell suspension was added to slides in the form of six drops per slide with a space in between. Cover slips were placed on the drops to embed the cells and allow gel solidification. After 10 min, the cover slips were removed. Slides were then immersed completely in ice-cold lysis solution and placed at 4°C for 1 hour in the dark. The slides were then placed in neutralization buffer for 15 min in the dark at 4 C. Slides were then shifted into 1X TAE buffer in the horizontal electrophoresis tank. The voltage was set to 1 V/cm, and current was supplied for 25 min. Staining was performed with 80 µL 1X Ethidium Bromide staining solution prepared from 10X (20 µg/mL) stock solution. The stain was left for 5 min, and slides were then washed in ice-cold water to remove excess stain. After drying at room temperature in the dark, the slides were observed at a ×40 objective lens in the blue channel of the fluorescence microscope for comet analysis, and images were captured. ImageJ and CASP software were used to analyze the images for comet tail analysis and the percentage of DNA in the comet tail. Multiple slides were prepared for one sample in three independent experiments to ensure the reproducibility of results.

### MTT assay

1.0 × 10^5^ Cells/mL were seeded 1 day before irradiation in 10 mL of RPMI1640 medium supplemented with 10% horse serum in T-25 flask. After 12 h, cells were processed for irradiation as explained above. Followed by irradiation, 100 μL of medium from the culture flask containing cells was taken and plated in a microplate (96-well plate) for 24 h. After 24 h, 20 µL of filter-sterilized MTT (5 mg/mL in PBS) was added to the micro wells containing cells. Following 3 h of incubation with MTT, the cell culture medium was discarded and the purple formazan crystals were dissolved in sterile DMSO (50 µL) by incubating at 37°C for 10-15 min. The absorbance at 490 nm was measured with a spectrophotometric plate reader. The absorbance values were converted to %Cell survival by the following formula;

$$\%\text{Cell survival}=\frac{\text{Absorbance}\;\text{of}\;\text{sample}-\text{Absorbance}\;\text{of}\;\text{blank}}{\text{Absorbance}\;\text{of}\;\text{control}-\text{Absorbance}\;\text{of}\;\text{blank}}$$

### Statistical analysis

Statistical analysis of gene expression was done on GraphPad Prism 5.04, and a tailed paired *t-test* was run between control and siRNA-transfected cells of the same sample for all three genes. For the cell viability and comet assay a two-way ANOVA was used. The *p*-values were calculated, and graphs were plotted with standard error mean values at a significance level of <0.05.

## Computational methodology

### Tanimoto similarity

The structural similarity between the investigated 69 compounds was evaluated using the RDKit program (Kunnakkattu et al., 2023). For each compound, the Tanimoto similarity index (Ts-index) (Vogt and Bajorath, 2017) was calculated against all studied compounds, and the results were visualized using a plot generated with OriginLab 2018 (RCSB, 2025).

### Molecular docking

The predictions obtained by docking simulations can provide useful information about the presence or absence of protein-ligand interactions, how these interactions are established (electrostatic, Van der Waals, hydrogen bonds, hydrophobic interactions), and which residues of the protein are involved. A representative protein X-ray crystal structure of Artemis with Uniprot ID: X6R6W9 and PDB ID: 7ABS (Yosaatmadja et al., 2021) was retrieved from the Uniprot (https://www.uniprot.org/) and RCSB Protein Data Bank (https://www.rcsb.org/), respectively. The BLAST program performed pairwise sequence alignment of these two Artemis proteins (https://blast.ncbi.nlm.nih.gov/Blast.cgi#). For molecular docking, the protein was prepared using the Protein Preparation Wizard module implemented in the Maestro (Schrödinger Release 2016-4: Maestro, Schrödinger, LLC, New York, United States) and optimized with OPLS-2005 force field. The zinc library of 69 compounds was downloaded from the ZINC database (Irwin and Shoichet, 2005) in SDF format and prepared by the LigPrep module in Maestro. The Receptor Grid Generation tool was employed to create a grid box around amino acid residues involved in interaction with the DNA. Finally, ligands were docked at the binding site with the Glide module, and G-scores and E-model scores were recorded for each ligand (Friesner et al., 2004). To perform a docking with AutoDock4, the protein and ligand structure were prepared using MGL tools and saved in. pdbqt format. The grid box was created around the key amino acid residues, and box size/dimensions were recorded to generate a grid parameter file. Finally, a docking parameter file was generated to perform docking. The results were saved as the docking log file, containing information about the binding energies for energetically favorable ten conformational poses of each ligand (Siddique et al., 2024; Farhan et al., 2024).

### ADMET studies

The ADMET properties of the compound library were predicted with the online web server tool ADMETlab-3.0 (Valencia et al.; Zadorozhnii et al., 2022; Xiong et al., 2021). Employing a quantitative structure-property relationship (QSPR) model trained by a multi-task graph attention (MGA) framework (Xiong et al., 2021).

### Density function theory

In the current study, all the computations were done using the Gaussian 09 W software program (Frisch et al., 2009). The molecular geometry of the investigated compounds was subjected to convergence using DFT with the B3LYP functional (Becke, 1993) and 6-311 + g (d,p) basis set (Clark et al., 1983), (Tirado-Rives and Jorgensen, 2008). The vibrational frequencies of the studied compounds were recorded at the same theoretical level to confirm the true global minima of optimized structures. To define the molecular reactivity/kinetic stability of investigated compounds, DFT was employed to calculate global and local reactivity descriptors such as frontier molecular orbital (FMO) energies, HOMO/LUMO energy gaps, and corresponding softness/hardness parameters. Furthermore, the chemical potential, electronegativity, ionization potential, and electron affinity were recorded as reactivity parameters (Bilal et al., 2022; Aziz et al., 2022b), followed by visualization of molecular electrostatic potential (MEP) maps to investigate the nucleophilic and electrophilic attack sites (Yaqoob et al., 2025).

### Molecular dynamics simulation

The 3-D models of protein-ligand complexes were generated in. pdb format using Maestro. They were subjected to molecular dynamics (MD) simulation in the GROMACS 2022.2 software program (Abraham et al., 2022) to evaluate the dynamic behavior of these complexes. The topology parameters for protein were established by the pdb2gmx module, employing the CHARMM27 force field (MacKerell et al., 1998), whereas ligand topology parameters were established using the cegenFF server (https://cgenff.com/). The simulation box of cubic shape was developed, and complexes were centered here under periodic boundary conditions. The system was solvated with water molecules using the TIP3P model (Jorgensen et al., 1983), and Cl^−^ ions were added to neutralize the system. The 50,000 energy minimization steps were conducted using the steepest descent methodology, which is then followed by NVT/NPT equilibration steps, each for 100-ps time duration. The Leapfrog method was employed during NPT equilibration to couple protein, ligand, and other system components. The system’s temperature was maintained at 300K with 1 bar pressure using Berendsen thermostat and barostat coupling constants. The PME algorithm was employed to correct truncation errors arising from the Coulomb interaction cutoffs at 1.2 nm. Finally, MD simulation was performed for a 100 ns period at isothermal/isobaric conditions, and trajectories were visualized in VMD1.9.2 (Humphrey et al., 1996). The MM-GBSA binding free energy calculations were performed using a dielectric constant of 80 for the solvent and 1 for the solute, which are standard values for implicit solvent models (Sun et al., 2014). The Xmgrace 5.1.19 (Turner, 2005) was employed for graph analysis (Lindahl et al., 2001).

### PCA and FEL

To conduct the PCA (Abdi and Williams, 2010), the gmx covar tool within the GROMACS suite was employed to compute the covariance matrix, which helped to assess the correlation between atomic fluctuations in the protein-ligand complex. The corresponding eigenvalues and eigenvectors were extracted via the gmx analog module, and projections on principal component (PC) coordinates were visualized for each frame. Additionally, the FEL analysis was carried out using the gmx sham module in GROMACS, allowing us to identify equilibrium and transition states that characterize protein-ligand complex stability (Papaleo et al., 2009).

## Result and discussion

### Micronucleus (MN) frequency assay

**FIGURE 1:**
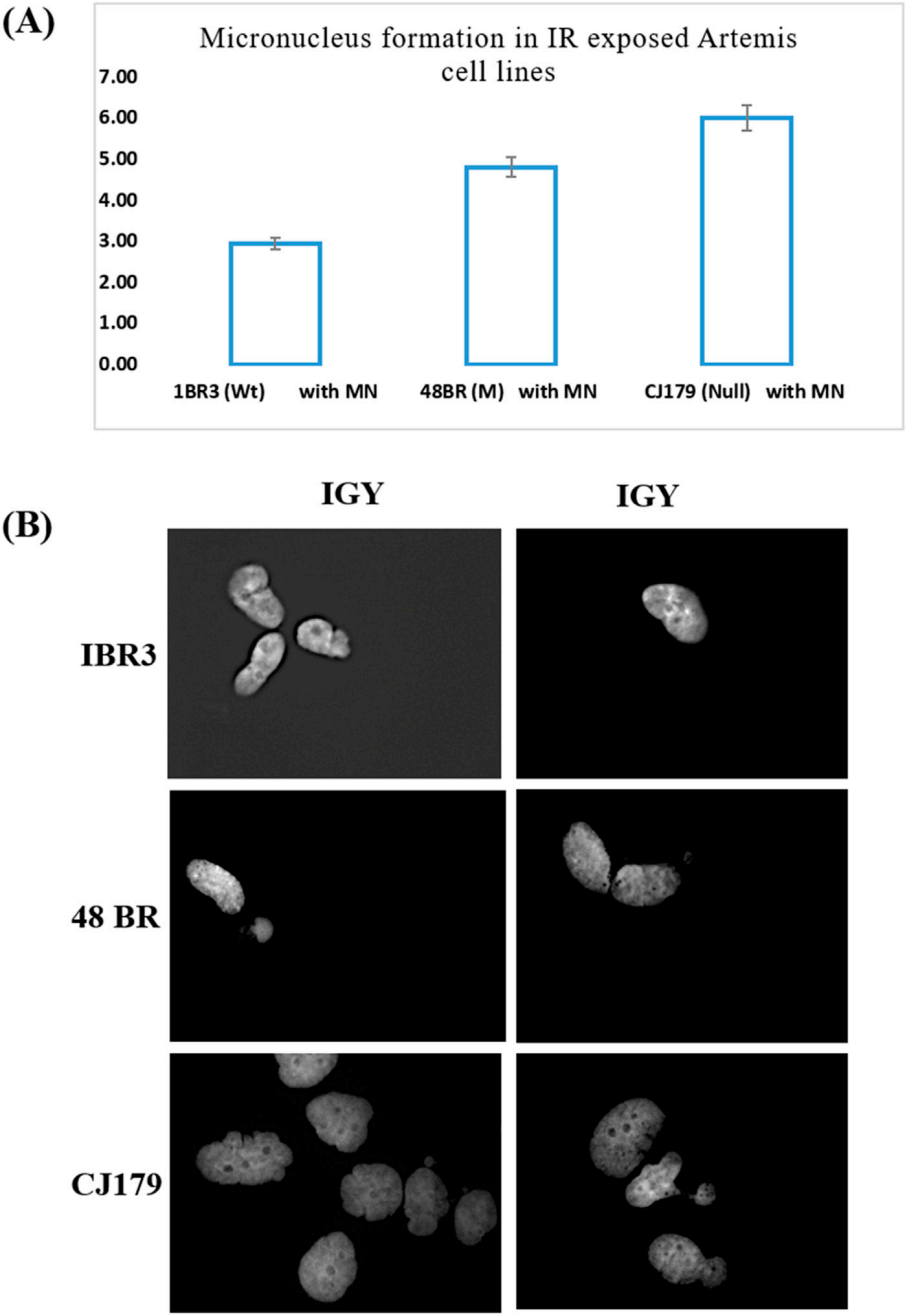
Micronucleus frequency assay: **(A)** Micronucleus formation in IR (1 Gy) exposed Artemis cell lines. The percentage frequency in each cell line was plotted. **(B)** Representative micrographs of micronucleation in Artemis cell lines following exposure to 1 Gy ionizing radiation (Mag x 400).

Our investigation showed that gamma-ray irradiated (1 Gy) Artemis-deficient cell line CJ179 had a higher number of unresolved DNA damage as measured indirectly using MN frequency assay, while Artemis-mutated 48BR cell line could resolve only some of the damage as compared to the Artemis wild-type cell line 1BR3 (Figure 1A), highlighting the potential of Artemis protein as a target for cancer therapy. The Figure 1B represented the micrographs of micronucleation in Artemis cell lines following exposure to 1 Gy ionizing radiation.

### Primary cell culture

**FIGURE 2:**
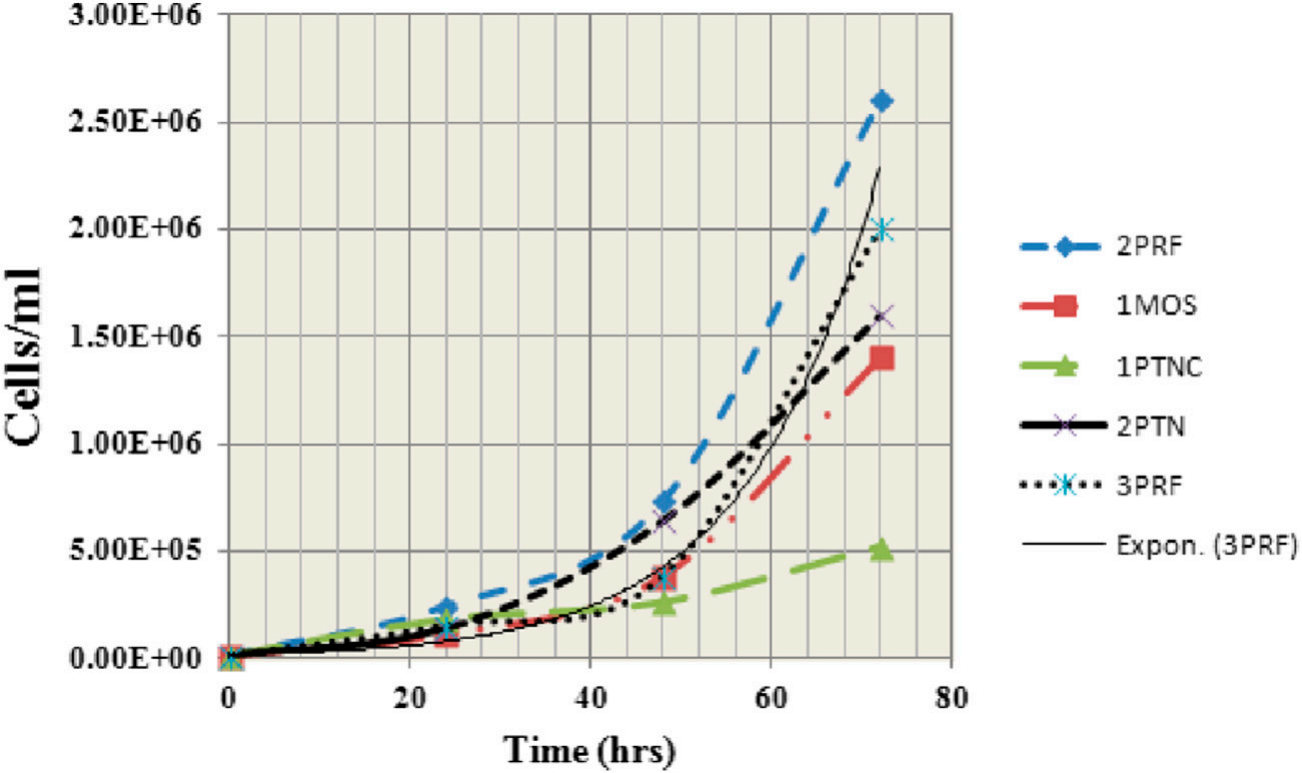
Graph showing growth curves of 5 primary cell lines (derived from invasive ductal carcinomas) at 0, 24, 48, and 72 h. Most aggressive growth is demonstrated by 1MOS and 2PRF whereas 1PTNC is shown to be the slowest in growth.

Samples (1MOS, 1PTNC, 2PTN, 3PRF and 2PRF) were observed for exponential growth starting from 1 × 10 ^4^ cells/mL to1x10^6^ cells/mL. Each sample showed a different growth pattern (Figure 2). 2PRF and 1MOS showed the most aggressive growth, reaching 5 times of initial number (2.4 × 10^6^ and 1.9 × 10^6^ cells/mL) after 24 h and 48 h of incubation, respectively. 1PTNC and 2PTN appeared to be slow growers, with almost double the number of cells after 24 and 48 h 3PRF was doubled thrice of the initial concentration at 24 and 48 h intervals. Morphological analysis of picked colonies after growth showed similarities with their parent colonies. Small cells clumped together to form colonies. Viable cells and colonies appeared to be transparent, whereas dead cells were seen as black and suspended in the medium.

### Artemis siRNA transfection of cells

**FIGURE 3:**
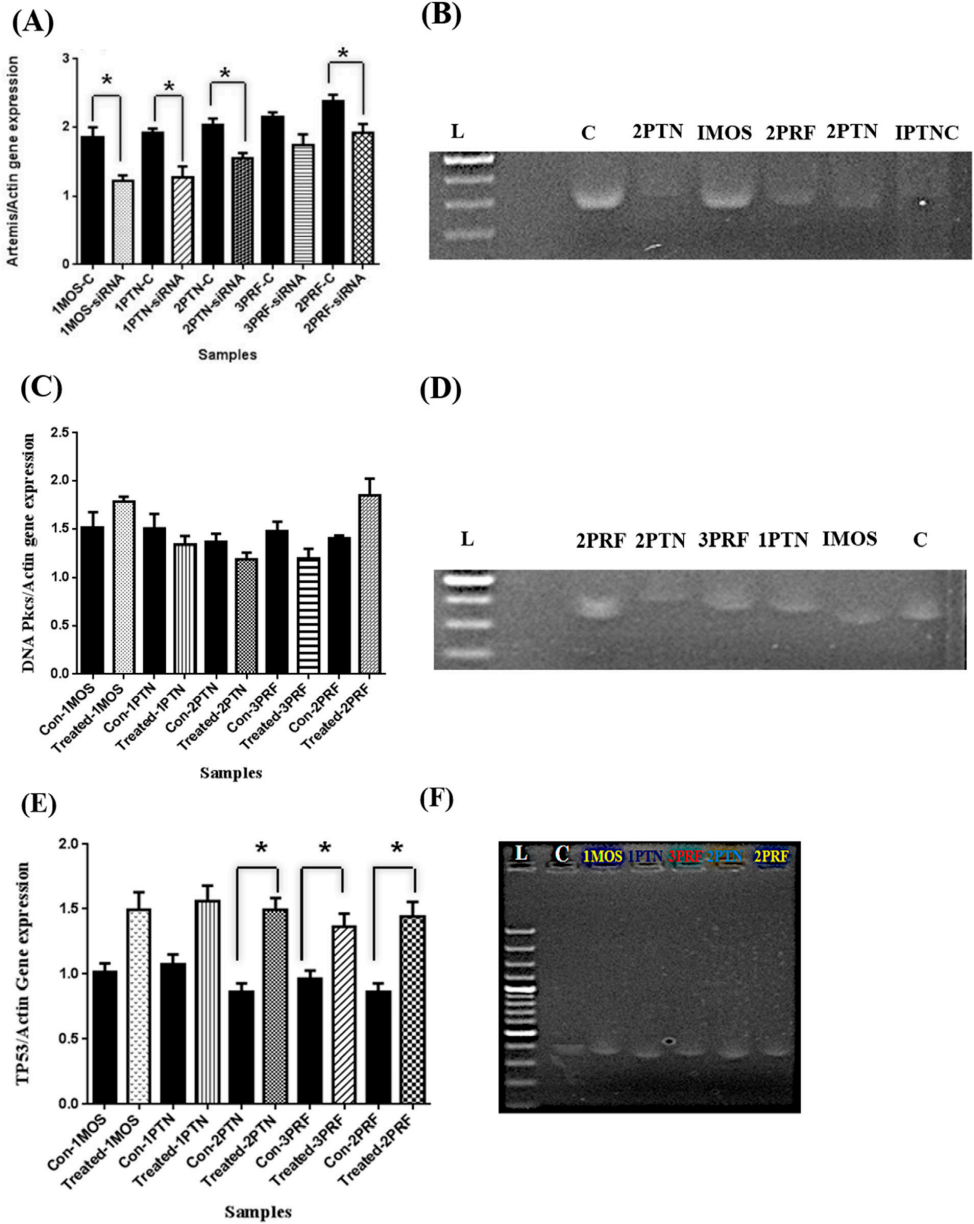
Artemis inhibition expression analysis after siRNA transfection in primary cell lines, Gene expression analysis of DNA-PKcs after inhibition of Artemis, and Gene expression analysis of TP53 after Artemis inhibition. **(A)** The graph represents the expression of the Artemis gene after siRNA transfection. Error bars are Standard Error Mean (SEM). X-axis has samples and Y-axis has Artemis/Actin gene expression values **(B)** Gel electrophoresis picture of amplified cDNA of Artemis gene and inhibition in the Breast cancer samples as compared to control, bands were analyzed by ImageJ (C = control, L = ladder 1000bps and dot in lPTN is gel doc error) **(C)** Graph shows the change in expression of DNA-PKcs with no significance. Values were calculated by dividing DNA PKcs bands measurement by Actin values and **(D)** Gel electrophoresis of DNA-PKcs amplified PCR products (C = control, L = ladder 1000bps) **(E)** Bar graph shows the expression analysis done by ImageJ and TP53 values divided by Actin band values, error bars are Standard error mean (SEM) with significance (p-value <0.05) in 2PTN, 2PRF and 3PRF in paired t-test. **(F)** TP53 amplified on a 2% agarose gel, which shows an increase in gene expression. (C = control, L = Ladder 1000bps, and the dot in mid of the bands is a gel doc error).

Expression analysis was done for the Artemis gene by preparing the cDNA. Artemis gene expression values were divided by the Actin gene to normalize, while analysis of gene expression on ImageJ provided the results of a 40 percent decrease in Artemis gene expression level as compared to control in 1MOS and 1PTNC. In 2PTN, 30 percent whereas around 20 percent decrease in 3PRF and 2PRF samples was observed (Figures 3A,B). Statistical analysis provided significance in samples except 3PRF as compared to the control. The *p*-value of all samples except 3PRF was less than 0.05.

### Gene expression analysis of DNA-PKcs

In gene expression analysis of DNA-PKcs, 1MOS and 2PRF showed an increase in DNA-PKcs gene expression by 10 and 30 percent, respectively. The increase in gene expression shown by IMOS was not significant compared to 2PRF. whereas, other 3 samples had a downregulation effect. 1PTN showed no decreasing trend as compared to 2PTN and 3PRF, which showed almost up to 15 percent and 30 percent decrease, respectively (Figures 3C,D). The *p*-value of all samples was greater than 0.05.

### Gene expression analysis of TP53

Increase or decrease in the TP53 gene, which encodes p53 protein, was also checked before and after inhibition of Artemis. Interestingly, all samples of breast cancer showed a significant increase in p53 gene expression after Artemis inhibition. 2PTN was seen to have the highest increase in p53 gene expression, which was almost 46%. Almost similar trend was observed in 2PRF, whereas 1PTN and 1MOS showed a similarity in increased expression of 38%. 3PRF conferred the lowest increase in expression level of 20% as compared to the control (Figures 3E,F). The *p*–values are >0.05.

### MTT assay

**FIGURE 4:**
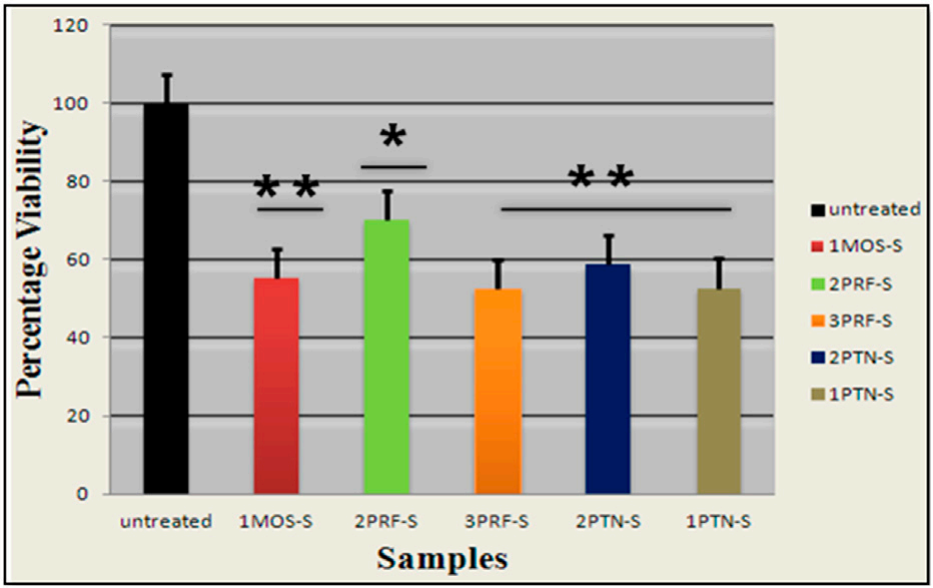
Percentage viability of the cells transfected with siRNA is plotted on the Graph. (p-value <0.05 one star significance and p-value <0.01, two star significance).

The percentage viability of siRNA-transfected cells was determined with an MTT assay, in which conversion of yellow tetrazolium MTT to purple formazan was quantified at dual optical densities of 490 nm and 630 nm. Control has the darkest color of purple formazan, which means that the darkest the color, the higher the optical density, because of a high number of proliferating cells. The highest decrease in percentage cell viability was observed in 1PTN and 3PRF, in which cells lost 50% viability. IMOS and 2PTN showed 60% cell viability, which was decreased by 40%, and lastly, 2PRF showed 70% cell viability (Figure 4).

### COMET assay

To confirm if Artemis inhibitor could be used a novel radiosensitizer we analyzed the DNA damage against different doses of radiation and the results indicated more DNA damage at low dosage of radiation compared to the higher doses. This consequence could be explained due to the activation of DNA repair pathways at high dose of radiation. To check this possibility we blocked NHEJ pathway by inhibiting key factor of NHEJ repair i.e., DNA PKcs; As a result, there was significant increase in accumulated damage over 24 h after high dose radiations. These results ensure that the NHEJ is the main pathway for repair of IR induced damage and that repair system activation at high dose of radiation is the reason for less DNA damage and cell killing rather than low dose of radiation. We also inhibited the NHEJ by using same strategy followed by low dose of radiation but the effect was not remarkable leading to the conclusion that at low dose DNA repair pathways are not activated. We observe that DNA PKcs inhibition impairs cells in IR induced damage repair. The effect of 10 μM concentration of DNA PKcs specific chemical inhibitor (NU7026) was analyzed for its effect on percent cell survival against different dose of radiation and it was found out that it almost decrease the cell survival by 50% at 4Gy of radiation. In the second aspect of our study, we analyzed the effect of artemis inhibition in response to IR induced DNA damage. In this approach we inhibited artemis by using 2-hydroxy-5-methoxybenzaldehyde-4-anilino-6-(3,5-dimethyl-H-pyrazol--yl)-,3,5-triazin-2-ylhydrazone chemical inhibitor.

**FIGURE 5:**
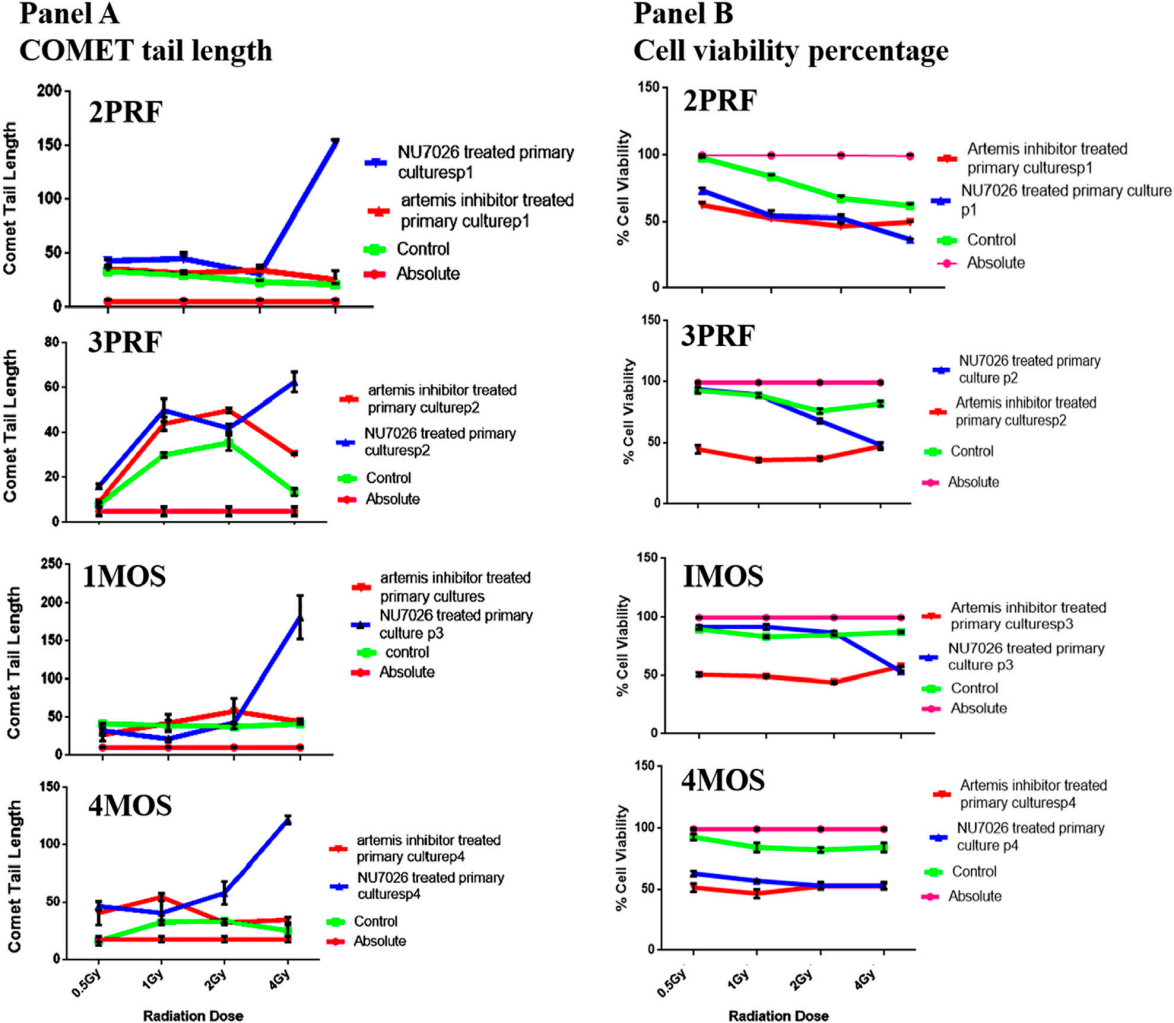
Panel **(A)** shows the graphical plots of comet tail lengths (in A.U.) for 4 primary cell lines (2PRF, 3PRF, 1MOS, and 4MOS), while panel **(B)** shows the cell viability measured with the MTT assay in these primary cell lines. These cell lines were treated with either Artemis inhibitor or DNA PK inhibitor to compare the effects of radiation exposure at 0.5, 1.0, 2.0, and 4.0 Gy.

Single Cell gel electrophoresis assay and MTT assay carried out after IR predicted that role of artemis in cell cycle arrest/apoptosis that is independent of DNA damage repair as shown in Figure 5 (Panel A and Panel B). Repair process was seen to be little defective in response to artemis inhibition as compared to control and the change was negligible. Percentage cell proliferation after IR was remarkably decreased which predicts the role of Artemis in cell cycle check points. Artemis inhibition caused almost 50% decrease in cell survival at 0.5 Gy of IR. This sensitivity does not affect the NHEJ as DNA damage was not accumulated in cells when analyzed after 24 h.

### Tanimoto similarity

**FIGURE 6:**
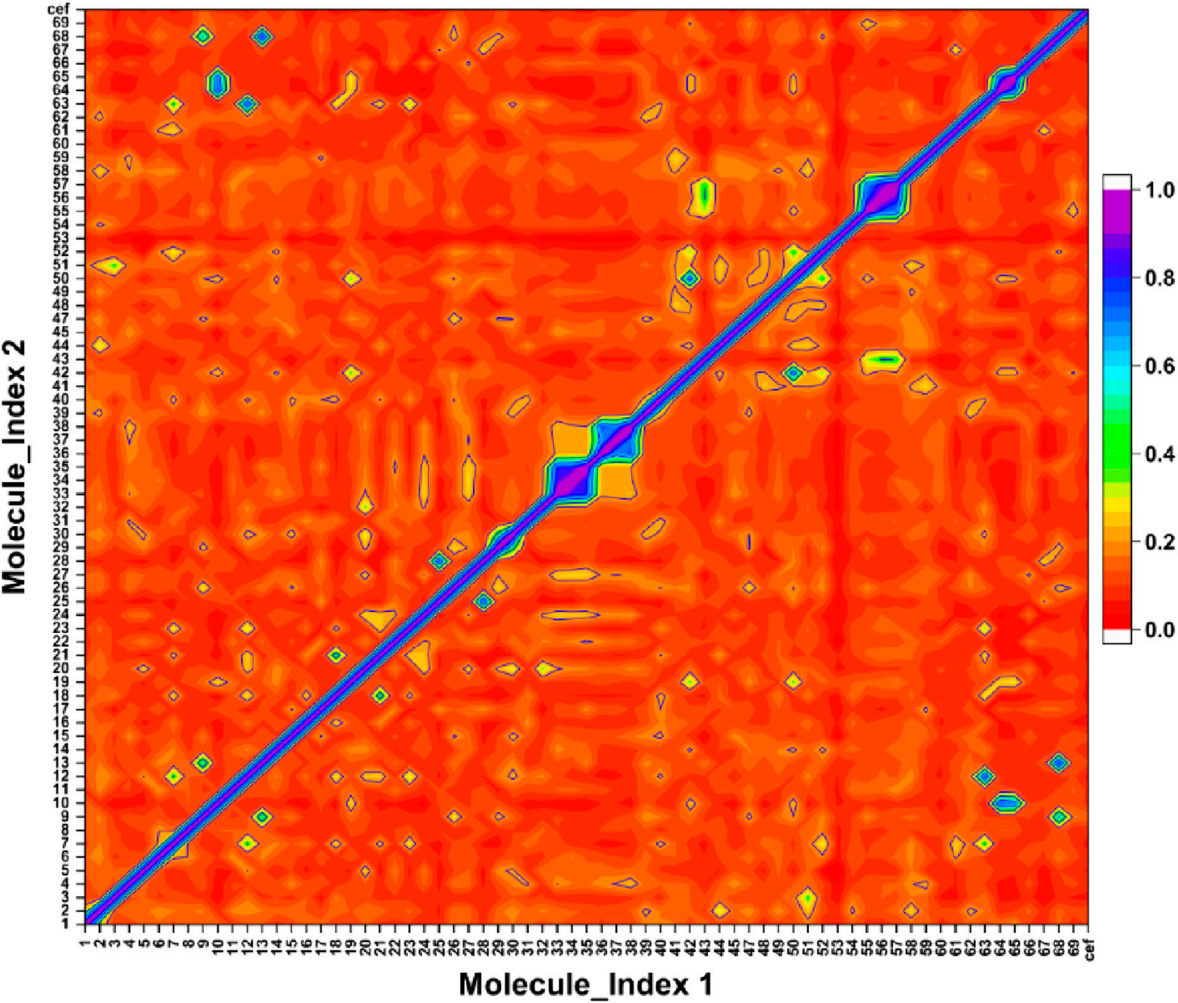
The symmetric matrix shows self-similarity values of 1.0 on the diagonal. The color scale represents pairwise similarity, with purple indicating high similarity and red indicating low similarity.

The Ts-index was computed for the 69 compounds, including the reference. The Figure 6 presented a heatmap that visualizes the pairwise similarity of these compounds using the Ts-index. The Ts-index, widely applied in cheminformatics, measures molecular similarity by comparing structural features. These features are typically represented as binary fingerprints, where each bit signifies the presence or absence of a particular molecular attribute.

On both the x-axis and y-axis, the 69 compounds were numbered from 0 to 68, and the reference was labeled as cef. The heatmap displayed the similarity score for the compounds corresponding to the intersecting data points. The scores ranged from 0 to 1, with a score of 1 (purple highlighted) indicating identical or highly similar compounds, while a score of 0 (red highlighted) represented no similarity. The diagonal line running from the top-left to the bottom-right represents each compound’s comparison with itself, resulting in a purple line with a perfect similarity score of 1. The off-diagonal line indicated the varying levels of similarity between different compounds, with colors transitioning from purple to red as the similarity decreases.

### Docking studies

**FIGURE 7:**
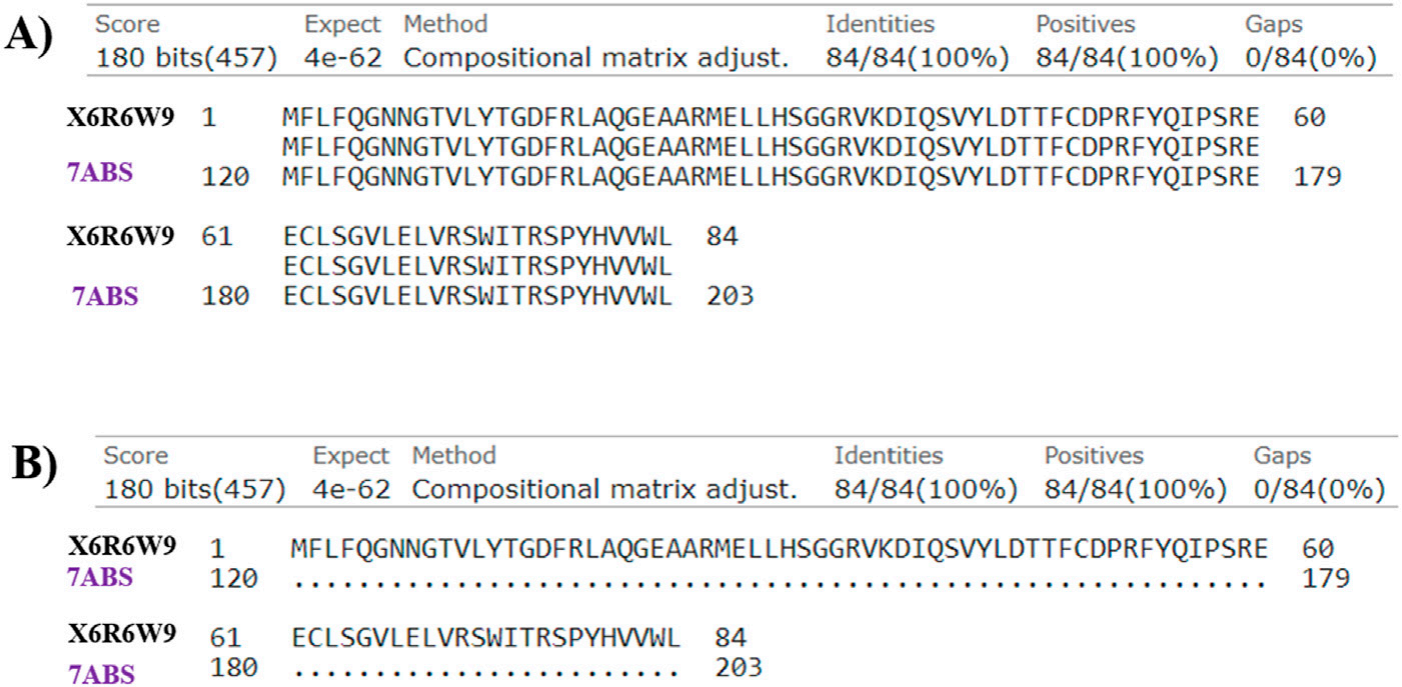
The sequence alignment of investigated Artemis proteins (Uniprot ID: X6R6W9 and PDB ID: 7ABS) in **(A)** pairwise and **(B)** dot representations.

The Artemis protein comprised two major domains: the Metallo-β-lactamase domain (MBL) and the β-CASP domain. The Zn ion in the MBL domain served as a catalytic center for the endonuclease activity of Artemis. The motif I–motif IV (ASP17, HIS33, HIS35, ASP37, HIS38, HIS115, ASP136) and motif C (VAL341), in the MBL domain, were involved in co-ordination with Zn ion in the catalytic site. In contrast, motifs A and B (ASP165 and HIS319) stabilize the product during catalytic reactions. The zinc-finger motif in the β-CASP domain (HIS228, HIS254, CYS256, CYS272) is involved in structural changes in Artemis, constituting the non-catalytic binding site. DNA’s highly negative phosphate groups coordinate with the Zn ion in the catalytic site. In addition to this, the positively charged amino acid residues, including ARG18, ARG21, LYS36, LYS40, ARG43, and LYS74 of the MBL domain and residues such as ARG 172, ASN205, LYS207, and LYS288 of β-CASP domain recognized negatively charged DNA as the substrate. This represented the extended open substrate binding pocket of Artemis located between the MBL domain and β-CASP domain (Yosaatmadja et al., 2021; Karim et al., 2020). The 7ABS incorporates Zn ion, crucial for protein activity, in its structure that is not present in X6R6W9. Furthermore, the sequence alignment analysis represented the 100% similarity of amino acid residues (120–203) of 7ABS with amino acid residues (1-84) of X6R6W9, indicating the metallo-β-lactamase domain of Artemis protein only, as shown in Figure 7. Based on the above analysis, which was 100% similar, the Artemis protein with PDB ID 7ABS was selected to understand better compound interactions with Zn ions within the binding pocket.

Molecular docking studies revealed the significant binding affinity of investigated compounds with 7ABS that displayed coordinate covalent bonds, ionic interactions with Zn ions, and H-bonding interaction with key amino acid residues. The compounds 01, 02, 04, 05, 42, 43, 44, 46, 51, and 58 were identified as top hits by the Glide module with ∆G-scores ranging from −9.1 kcal/mol to −8.0 kcal/mol. The compounds 23, 46, and 49 were selected by AutoDock4 screening with docking scores above −8.0 kcal/mol. However, compounds 28, 55, 59, and 67 demonstrated comparable binding affinities in both, Glide and Autodock4 predictions. The G-scores, Emodel scores, hydrogen bonding, hydrophobic, and ionic interactions of top hits with Artemis endonuclease are outlined in Table 4.

**TABLE 4:** Molecular Glide score, Hydrogen bonding interactions (Å), hydrophobic and other interacting residues for ligands with Artemis protein (PDB ID: 7ABS).

Ligands	G-score (kcal/mol)	Emodel	Autodock4	Ionic/H.B.I residue (distance Å)	Hydrophobic and other interacting residues
01	−8.5	−81.83	−5.88	ASP136 (2.44) THR167 (1.89) Zn 101 (2.00) Zn 402 (3.89)	HIS35, ASP37, THR167, LYS207, ALA209, TYR212, PRO289, SER290, THR291, PHE318, HIS319, VAL341, PRO343, VAL344
02	−8.1	−86.60	−5.62	ASP136 (2.44) THR291 (2.74) Zn 101 (2.20) Zn 402 (3.45)	HIS35, LYS36, ASP37, HIS115, THR167, PHE168, LYS207, ALA208, THR291, SER317, PHE318, HIS319, VAL341, PRO343, VAL344
04	−8.6	−93.86	−5.89	HIS115 (3.09) HIS319 (2.38) Zn402 (2.02)	HIS35, ASP37, HIS115, ASP136, LYS207, ALA209, PHE318, HIS319
05	−8.5	−97.93	−7.20	ASP136 (2.44) PHE318 (3.17) Zn 101 (2.23) Zn 402 (3.37)	HIS35, ASP37, HIS115, PHE168, LYS207, TYR212, SER290, THR291, TRP293, PHE318, HIS319
23	−4.5	−51.63	−8.1	CYS34 (3.20) HIS35 (3.02) HIS35 (3.06) LYS36 (2.17) LYS207 (4.53) TYR212 (2.44) PHE318 (3.11)	HIS35, LYS36, LYS207, ALA209, PHE318
28	−6.8	−73.87	−7.0	LYS36 (2.64) ASP37 (1.67) ASP37 (3.81) LYS207 (2.02) LYS207 (4.63)	LYS207, ALA209, PHE318
42	−8.6	−139.47	−8.03	LYS36 (1.92) LYS36 (3.47) ASP136 (2.44) ALA208 (2.79) TYR210 (1.81) TYR210 (3.25) HIS319 (2.40) HIS319 (4.72) Zn 101 (2.04) Zn 402 (3.43)	CYS34, HIS35, ASP37, LEU68, SER72, PRO73, HIS115, THR167, LYS207, ALA208, ALA209, TYR210, PHE318
43	−8.1	−114.32	−6.26	ASP37 (2.32) ASP136 (2.44) HIS319 (4.72) Zn 101 (2.04) Zn 402 (3.43)	CYS34, HIS35, LYS36, ASP37, HIS115, THR167, LYS207, ALA208, ALA209, TYR210, PHE318, HIS319
44	−8.0	−81.99	−6.29	ASP136 (2.44) PHE318 (2.62) HIS319 (4.46) Zn101 (2.19)	HIS35, ASP37, THR167, LYS207, ALA209, TYR212, THR291, SER317, PHE318, HIS319, VAL341, PRO343
46	−9.1	−112.52	−7.00	PRO289 (2.77) HIS319 (2.10) PHE316 (2.44) Zn402 (3.91)	HIS35, LYS36, ASP37, LYS207, TYR212, PRO289, SER290, THR291, PHE316, SER317, PHE318
49	−6.2	−86.81	−8.1	HIS35 (3.24) LYS36 (2.06) ASP37 (2.13) LYS207 (4.87) ALA208 (1.92)	HIS35, LYS36, LYS207, ALA209, PHE318
51	−9.4	−133.21	−7.69	HIS319 (4.59) Zn402 (3.89)	HIS35, LYS36, ASP37, THR167, LYS207, ALA208, ALA209, VAL341, PRO343, VAL344
55	−7.6	−99.68	−7.1	ASP37 (1.89) ASP136 (1.92) THR219 (3.07) SER317 (3.79) PHE318 (1.91)	PHE168, LYS207, ALA208, ALA209, PHE318
58	−8.4	−105.72	−6.68	ASP136 (2.44) PHE316 (2.56) THR291 (1.82) HIS319 (3.07) Zn101 (2.13) Zn 402 (3.06)	HIS35, THR167, LYS207, TYR212, SER290, THR291, TRP293, PHE316, SER317, PHE318
59	−7.1	−99.68	−6.8	HIS35 (3.37) HIS35 (3.79) ASP37 (3.36) ASP37 (5.19) ASP136 (2.07) ASP136 (1.73) PHE318 (2.36) HIS319 (2.40)	LYS36, LYS207, ALA208, ALA209, TYR212, PHE318
67	−6.5	−93.25	−6.4	HIS35 (3.17) ASP37 (4.00) ALA208 (2.31)	HIS35, LYS36, ALA209, TYR210, PHE318
Ceftriaxone	−9.9	−165.02	−6.6	HIS35 (1.54) LYS36 (2.13) HIS115 (3.29) PHE318 (3.63) HIS319 (2.20) Zn402 (3.93)	CYS34, HIS35, ASP37, THR167, PHE168, LYS207, ALA208, ALA209, TYR210, TYR212, THR291

**FIGURE 8:**
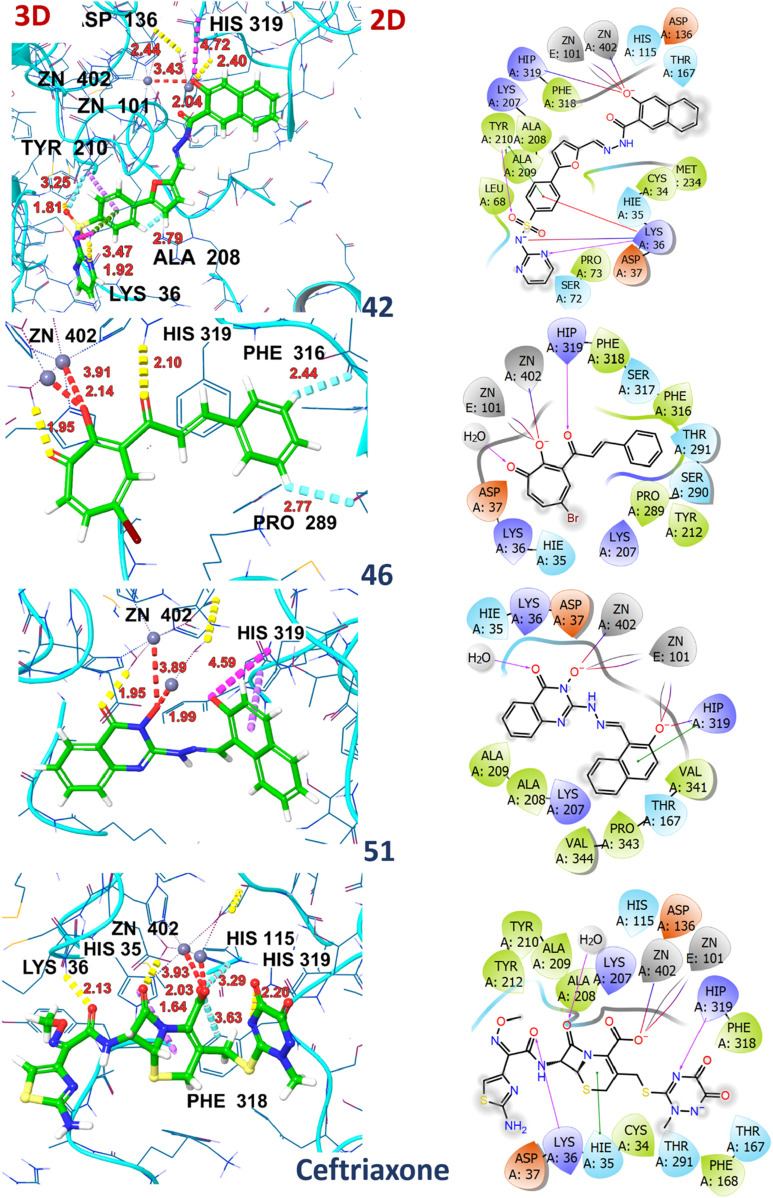
2D and 3D interactions of compounds 42, 46, 51, and ceftriaxone with 7ABS protein.

The ligands 01 and 02 showed H-bonding interactions with ASP136 via conserved water molecules. In contrast, the inhibitory activity of ligand 04 is attributed to the hydrogen bonding with the amino acid residue of motif B (HIS319). Supplementary Figure S1 illustrates the 2D and 3D interaction of ligands 01, 02, and 04 with the target protein. The substantial binding affinity of compound 42, with a ∆G-score of −8.6 kcal/mol, is attributed to strong electrostatic and H-bonding interactions with LYS 36 and HIS319 in the catalytic domain. Compound 43 displayed H-bonding interactions with ASP37 and ASP136 in the MBL domain and ionic interaction with HIS319 in the catalytic binding site, as shown in Supplementary Figure S2. Compound 44 displayed π-π interaction with amino acid LYS207 of the β-CASP domain that stabilizes the substrate. It also exhibited halogen and ionic interactions with PHE318 and HIS319 with a bond distance of 2.62Å and 4.46Å, respectively. The compound 46 exhibited π-hydrogen bonding interactions with amino acid residues PRO289 and HIS 319 with distances of 2.77Å and 2.44Å, respectively. Similarly, compound 51 exhibited ionic interactions and π-π stacking with amino acid residue HIS319 (Supplementary Figure S3). The residue HIS319 of motif B interacts with ASP165 (motif-A) via H-bonding and is considered critical for Artemis’s endonuclease activity. Additionally, the inhibitory activity of all top hits is attributed to the coordination with the Zn ion, which is crucial for the enzyme’s catalytic activity. The high binding affinity was observed for compounds 42, 46, and 51 with ∆G-scores of −8.6 kcal/mol, −9.1 kcal/mol, and −9.4 kcal/mol, respectively. The similar binding affinities (−8.1 kcal/mol) were recorded for compounds 23 and 49 by the AutoDock4 program, showcasing H-bonding interactions with key residues such as HIS35, ASP37 (MBL domain), and LYS 207 (β-CASP domain). Compounds 28, 42, 55, 59, and 67 demonstrated significant binding affinities, recorded by both software programs (AutoDock4 and Glide), showing a docking score deviation of −0.5 kcal/mol. The ceftriaxone exhibited moderate inhibitory activity against Artemis protein with an IC_50_ value of 65 µM (Yosaatmadja et al., 2021; Melenotte et al., 2021) by *in-vivo* studies, used as a reference for result comparison. The significant binding affinity of ceftriaxone with a Glide score of −9.9 kcal/mol is attributed to its metal co-ordination as well as H-bonding interaction with conserved amino acid residues such as HIS35, HIS115, and HIS319, as illustrated in Supplementary Figure S4. The 2D and 3D interactions of compounds 42, 46, 51, and ceftriaxone with the target protein are illustrated in Figure 8. The docking scores of all compounds, recorded by AutoDock4 and the Glide program, were tabulated in Supplementary Table S2 (Supplementary Material).

### ADMET analysis

The ADMET properties of top-hit compounds are presented in Table 5 and Table 6. All the top hits exhibited molecular weight of less than 500 Da except for compound 42 and the reference drug. The compounds 43 and 67 showed significant lipid solubility with high log P values of 4.84 and 4.73, respectively, representing their passive absorption from the GI tract. The TPSA value indicates the polarity of compounds and should be less than 140Ǻ for optimal pharmacokinetic properties. The compound 59 and reference compounds are considered highly polar among the investigated compounds with TPSA values of 152.84Å and 215.69 Å. Almost all the compounds were found to be the substrate of P-glycoprotein, resulting in decreased bioavailability. In contrast, the inhibition of P-glycoprotein may enhance the bioavailability of compounds. However, compounds 28 and 46 were found to mimnimally inhibit P-glycoprotein among the investigated compounds, with values of 0.90 and 0.99, respectively, affecting their bioavailability. Compounds 05 and ceftriaxone demonstrated poor human intestinal absorption among the top hits. Most compounds displayed high Caco-2 membrane permeation with values > −5.0 cm/s, contributing to increased oral absorption. The investigated compounds exhibited significant plasma protein binding affinity (>90%), indicating a lower unbound fraction in plasma. However, compounds 04 and 05 showed 71.21% and 87.62% fractions bound with plasma protein, allowing a significant fraction of free drug to reach its target site.

**TABLE 5:** The ADMET properties include physicochemical parameters, absorption, distribution, and bioavailability of top-hits.

Compounds	MW	TPSA	LogP	P-gp inhibitor	P-gp substrate	HIA	F (20%)	Caco-2	PPB
1	357.17	104.45	2.27	0.39	0.00	0.02	0.11	−5.10	97.93
2	348.12	100.01	2.74	0.09	0.02	0.01	0.01	−5.16	98.10
4	179.06	69.89	0.72	0.00	0.04	0.02	0.06	−5.42	71.21
5	324.11	77.92	1.12	0.69	0.11	0.72	0.09	−4.56	87.62
23	349.13	56.49	3.41	0.61	0.00	0.00	0.02	−4.13	97.48
28	321.1	72.47	3.22	0.90	0.00	0.34	0.04	−4.97	97.59
42	513.11	146.78	3.74	0.00	0.04	0.00	0.92	−5.85	98.81
43	430.19	122.37	4.84	0.05	0.06	0.00	0.64	−5.14	98.87
44	342.1	101.16	3.60	0.03	0.00	0.00	0.03	−4.86	98.62
46	329.99	54.37	3.29	0.99	0.00	0.00	0.04	−4.89	98.88
49	355.11	96.7	3.97	0.00	0.02	0.00	0.01	−4.97	98.67
51	346.11	99.74	3.33	0.00	0.19	0.00	0.81	−4.94	98.86
55	360.13	110.39	3.07	0.05	0.00	0.00	0.04	−4.85	98.20
58	345.11	94.48	2.78	0.02	0.04	0.00	0.04	−4.99	97.95
59	462.15	152.84	2.59	0.00	0.51	0.00	0.99	−5.46	97.73
67	433.13	88.9	4.73	0.56	0.00	0.00	0.11	−5.03	98.23
Ceftriaxone	554.05	214.96	−0.60	0.01	0.00	0.99	0.97	−6.58	92.00

MW, Molecular Weight; TPSA, Topological polar surface area; P-gp, p-glycoprotein; HIA, Human intestinal absorption; F20%, Bioavailability; Caco-2, Caco-2, cell membrane permeability; PPB, plasma protein binding.

**TABLE 6:** The ADMET properties include Blood-brain penetration, metabolism, and excretion regarding the half-life of top hits.

Compounds	BBB	CYP2D6 inhibitor	CYP2D6 substrate	CYP3A4-inhibitor	CYP3A4-substrate	T1/2 (hr)	Lipinski violation
1	0.00	0.00	0.00	0.00	0.99	0.69	0
2	0.66	0.00	0.00	0.00	0.72	1.10	0
4	0.00	0.00	0.00	0.00	0.26	1.53	0
5	0.10	0.00	0.96	0.51	0.01	1.20	0
23	0.81	0.06	0.99	0.98	0.37	0.69	0
28	0.00	0.99	0.00	0.95	0.00	0.52	0
42	0.00	0.00	0.00	0.00	0.99	0.63	1
43	0.00	0.00	0.02	0.99	0.01	0.54	0
44	0.00	0.00	0.24	0.00	0.00	0.71	0
46	0.83	0.01	0.01	0.00	0.00	0.98	0
49	0.78	0.00	0.00	0.02	0.06	0.86	0
51	0.00	0.01	0.01	0.89	0.01	0.78	0
55	0.00	0.10	0.00	0.98	0.00	0.90	0
58	0.09	0.99	0.98	0.99	0.01	1.25	0
59	0.00	0.00	0.00	0.01	0.00	1.08	2
67	0.00	1.00	0.74	1.00	0.09	0.73	0
Ceftriaxone	0.01	0.01	0.00	0.00	0.00	1.92	1

BBB, Blood Brain Barrier (enhanced penetration with values close to 1). T1/2: half life.

The low probability of crossing the blood-brain barrier is observed for compounds 28, 46, and 49 and is considered not associated with CNS-related toxicities. The CYP2D6 and CYP3A4 enzymes inhibition or induction may affect drug metabolism, therapeutic effect, and related toxicities. The high enzyme inhibition probability was observed with compounds 23, 28, 43, 51, 55, and 58. In contrast, the compounds 01, 05, 23, 42, and 58 were found to be the substrate of CYP2D6 and CYP3A4 metabolizing enzyme. The top hits demonstrated short half-lives with T½values ranging from 0.69 h to 1.53 h, representing their rapid elimination. The details of ADMET properties for all compounds are outlined in Supplementary Table S2 and Supplementary Table S3 (Supplementary Material).

## DFT results

### Optimized geometries

The bond lengths and angles were computed at DFT/B3LYP/6-311 + g (d,p) level of theory and are considered accurate from HF calculations, attributed to the inclusion of electron correlation. The optimized geometries of top hits at their most stable conformations are illustrated in Supplementary Figure S5. The optimization energies were computed as −628.95 a. u to −2,856.39 a. u with the lowest Hartree optimization energy value of −1773.27a.u., recoded for compound 67. Most top hits displayed non-planar geometry, particularly observed with compounds 01, 05, 42, 58, 67, and ceftriaxone, likely due to steric hindrance imposed by the adjacent substituents. However, compounds 02, 04, 23, 28, 43, 44, 51, and 55 showed somewhat planar conformations. Table 7 presented the top-hit compounds’ optimization energies, dipole moment, polarizability, and HOMO-LUMO gap values. The dipole moment (*μ*) is one of the significant electronic parameters that illustrates the uneven distribution of charges among atoms in a given compound and is often involved in intermolecular interactions such as dipole-dipole interactions. It is frequently used to study the intermolecular interactions involving the non-bonded type dipole-dipole interactions because the higher the dipole moment is, the stronger the intermolecular interactions. The compounds 01 and 02 displayed higher dipole moments of 9.33 and 10.22 debye, indicating stronger intermolecular interactions, a prerequisite for significant binding affinity with the target protein. However, compound 51 displayed a high binding affinity with the target protein with the lower dipole moment of 1.34 debye, representing that this parameter is not directly correlated with activity. Polarizability (*α*) refers to the ease with which electrons can be displaced from the compounds by an external electric field, reflecting the compound’s softness and reactivity. In general, high polarizability is associated with the softer nature of the compound (Chattaraj et al., 2003). Compound 42 displayed the highest polarizability of 470.71 a. u, among the top hits, with a lower energy gap of 3.46 eV. However, significant polarizability is also perceived for compounds 43, 59, and 67, along with high HOMO energy values ranging from −5.86eV to −5.92eV. The higher HOMO energies of these compounds were accompanied by easy electron displacement, attributed to their high electron-donating capability. The compound 04 showed lower HOMO energy values of −7.34eV indicating its inferior ability to lose electrons, considered to be less reactive as evidenced by a large energy gap of 5.37 eV. The lowest energy gap of 3.15eV is computed for compound 28, attributed to significantly minimum LUMO energy values of −3.34 eV, representing its greater competency to accept electrons. The energy gap of compound 02 is calculated as 3.06 eV, which is suggested to be the soft compound with electron-donating capacity and high reactivity.

**TABLE 7:** Energetic parameters of top hits using DFT in the gas phase.

Compounds	Optimization energy (a.u.)	Dipole moment (debye)	Polarizability (a.u.)	HOMO (eV)	LUMO (eV)	HOMO-LUMO ( ∆ eV)
01	−1,203.45	9.33	250.49	−6.15	−1.42	4.74
02	−1,178.75	10.22	299.02	−5.61	−2.55	3.06
04	−628.95	4.54	125.25	−7.34	−1.97	5.37
05	−1,106.30	7.70	245.19	−6.95	−3.11	3.83
23	−1,121.46	4.17	318.89	−6.32	−2.27	4.05
28	−1,089.04	6.89	270.76	−6.48	−3.34	3.15
42	−2050.71	6.64	470.71	−5.84	−2.38	3.46
43	−1,440.46	5.73	385.81	−5.92	−1.76	4.17
44	−1,482.96	5.17	307.50	−5.67	−2.20	3.47
49	−1,194.51	6.80	295.79	−6.19	−2.20	3.99
51	−1,177.42	1.34	308.20	−5.89	−2.21	3.68
55	−1,212.92	0.32	348.05	−5.71	−2.22	3.49
58	−1,161.42	4.09	288.30	−5.81	−2.35	3.45
59	−1,598.61	2.84	376.82	−5.86	−1.82	4.03
67	−1773.27	5.34	355.51	−5.91	−1.61	4.30
Ceftriaxone	−2,856.39	3.99	375.11	−6.09	−2.32	3.78

**FIGURE 9:**
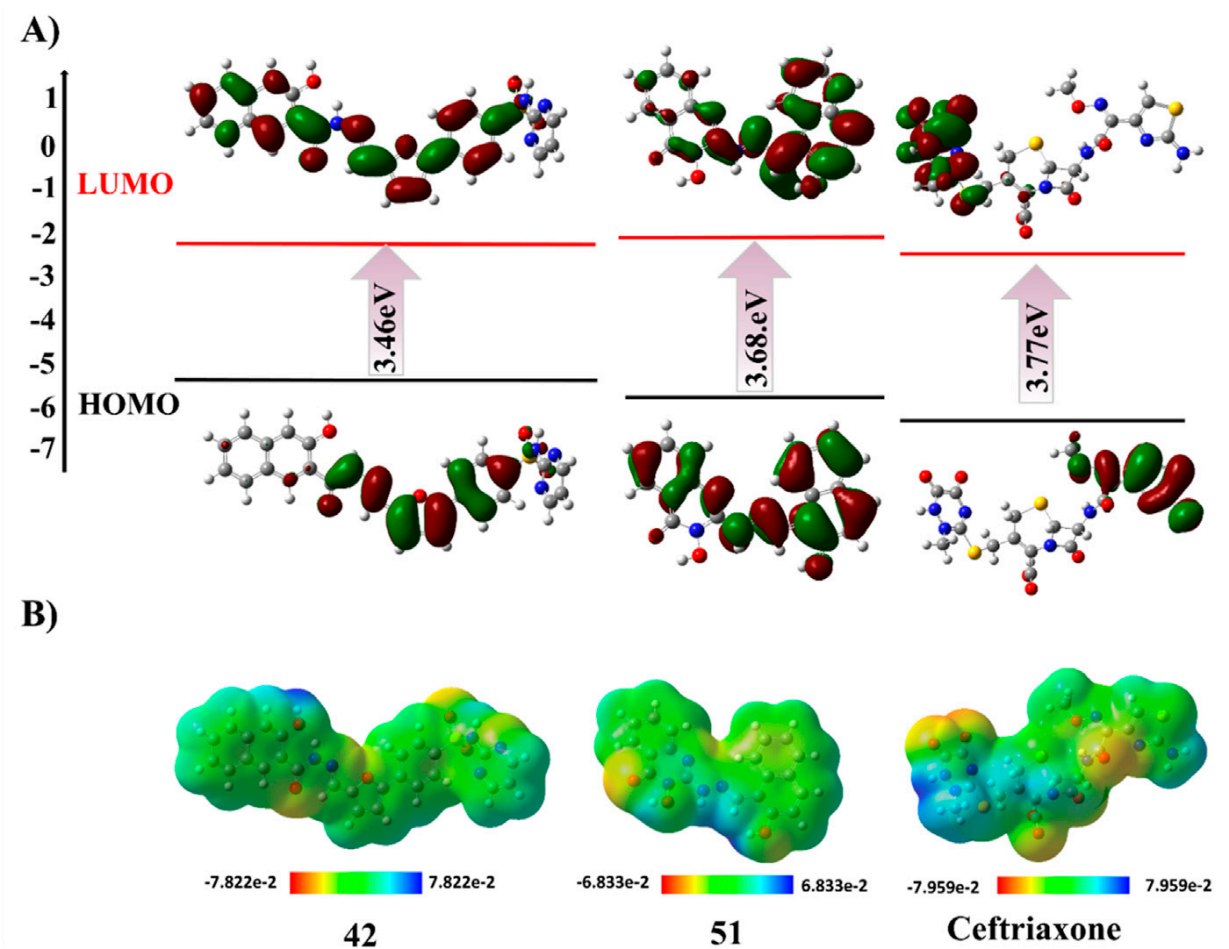
DFT studies of compounds 42, 51, and ceftriaxone. **(A)** HOMO-LUMO contour plot. **(B)** Molecular electrostatic potential map.

The Supplementary Figure S6 illustrates the HOMO-LUMO orbitals and their corresponding energy gap values for compounds 01, 02, 04, 05 and 23. The HOMO contour maps in compound 02 presented the electron density of electron-rich benzimidazole moiety, while LUMO contour maps were centered on the 2-oxo-quinoline moiety, highlighting its charge transfer character. Similarly, compound 23 showed a shift of electron density from electron-rich to electron-deficient regions in HOMO to LUMO contour maps. This charge transfer type is also observed with compounds 28, 42, and 43, as shown in Supplementary Figure S7. In compounds 01, 04, 44, 46, and 51, the HOMO/LUMO contour maps are located across the entire structure, attributed to extended conjugated systems in these compounds (Supplementary Figure S6; Supplementary Figure S8). Conversely, in compound 05, the HOMO and LUMO orbitals were centered on the particular moiety of the structure, indicating the reactive sites. Interestingly, the non-planar rings displayed negligible electron density with respect to the main nucleus in the chemical structures of compounds 58 and 67 (Supplementary Figure S9). The optimized geometries, HOMO-LUMO contour maps, and MEP plots of compounds 42, 51, and ceftriaxone are presented in Figure 9.

The physiochemical properties and quantum chemical descriptors for top hits are outlined in Table 8. The chemical potential (µ) represents the molecule’s inclination to lose or gain electrons, whereas lower µ values signify the greater tendency to capture electrons. The compound 05 and 28 showed lower chemical potentials of −5.03 and −4.91 eV, respectively; compound 01 and reference ceftriaxone exhibited the same chemical potential of −3.78 eV, whereas compound 67 demonstrated the highest potential (µ = −3.76 eV) in comparison to other compounds. Furthermore, compounds 02 and 28 were softer, with chemical softness values of 0.33eV and 0.32 eV, respectively, reflecting their high reactivity. In contrast, the softness potential was the same for compound 05 and ceftriaxone (0.26 eV). Substantive reactivity is also observed with compounds 42 and 51, correlated with their significant binding affinities. Compound 04 was considered the least reactive, with a hardness value of 2.69eV among the top hits. The high electronegativity for compound 05 (χ = 5.03 eV) indicates its greater electron-attracting ability, as evidenced by low LUMO energy and high electron affinity (A = 3.11eV). In contrast, compounds 01 and 67 showed low electronegativity values of 3.78eV and 3.76eV, respectively, representing their lower capacity to attract electrons. Electrophilicity (ω) quantifies the stabilization energy of a compound as it gains electrons. The results displayed high electrophilicity (ω = 7.66) for compound 28, suggested to be a good electrophile compared to other top-hit compounds. These reactivity parameters illustrated the chemical behavior of top-hit compounds and possible interaction types involved in their significant binding affinity.

**TABLE 8:** Physiochemical properties and Quantum chemical descriptors for top hits using DFT in the gas phase.

Compounds	Chemical potential (μ) (eV)	Electro-negativity (χ) (eV)	Chemical hardness (η) (eV)	Chemical softness (ζ) (eV)	Electro-philicity index (ω)	Ionization potential (I) (eV)	Electron affinity (A)
01	−3.78	3.78	2.37	0.21	3.02	6.15	1.42
02	−4.08	4.08	1.53	0.33	5.43	5.61	2.55
04	−4.66	4.66	2.69	0.19	4.04	7.34	1.97
05	−5.03	5.03	1.92	0.26	6.60	6.95	3.11
23	−4.30	4.30	2.03	0.25	4.55	6.32	2.27
28	−4.91	4.91	1.57	0.32	7.66	6.48	3.34
42	−4.11	4.11	1.73	0.29	4.89	5.84	2.38
43	−3.84	3.84	2.08	0.24	3.54	5.92	1.76
44	−3.93	3.93	1.73	0.29	4.46	5.67	2.20
46	0.00	0.00	0.00	0.00	0.00	0.00	0.00
49	−4.20	4.20	2.00	0.25	4.41	6.19	2.20
51	−4.05	4.05	1.84	0.27	4.45	5.89	2.21
55	−3.97	3.97	1.75	0.29	4.51	5.71	2.22
58	−4.08	4.08	1.73	0.29	4.82	5.81	2.35
59	−3.84	3.84	2.02	0.25	3.66	5.86	1.82
67	−3.76	3.76	2.15	0.23	3.29	5.91	1.61
Ceftriaxone	−3.78	4.20	1.89	0.26	4.68	6.09	2.32

The molecular electrostatic potential (MEP) surface is invaluable for understanding the allocation of electrostatic potential over the molecule’s isoelectronic density surface and aids in determining the regions of the molecule that are likely to be involved in binding interactions. The MEP maps displayed the molecule’s high and low electron density regions, presented by a standard color coding system. The red region indicated high electron density with a partial negative charge. In contrast, the blue region signified low electron density with a partial positive charge, turning yellow to green towards neutral regions. In the case of compounds 01, 02, 42, 43, 58, and 59, the positive blue region is centered on the hydrogen atoms bonded to phenolic oxygen, suggested to be a nucleophilic attack site with a maximum of positive electrostatic potential. However, the positive potential is localized on hydrogen atoms bonded to hydroxyl amine in compound 04 and on aromatic hydrogens of compounds 23, 28, 49, and 67, which are suggested to be susceptible to nucleophilic attack. Furthermore, compounds 51 and 55 displayed positive potential sites around the hydrazine and amino groups, respectively, indicating their significant interaction with nucleophiles. Furthermore, the MEP analysis revealed electrophilic attack sites with the most negative potential, centered on carbonyl oxygen and nitrogen atoms of diazole and azo functional groups in the chemical structure of top-hit compounds. The MEP maps of investigated compounds are illustrated in Supplementary Figure S10.

### MD-simulation analysis

The MD simulation analysis of compounds 42, 51, and ceftriaxone was performed by plotting root mean square deviation (RMSD), root mean square fluctuation (RMSF), number of H-bond contacts, and % occupancy of H-bonds with key amino acid residues over 100 ns molecular dynamic simulation along with MMGBSA binding energy calculations at various trajectories.

The RMSD plots of backbone atoms of Artemis in complex with investigated compounds revealed the structural stability of protein during the entire simulation, as shown in Supplementary Figure S11. The protein structure in complex with compound 51 and ceftriaxone showed great stability, with RMSD values of about 0.2 nm. In contrast, the Artemis 42 complex showed slight variation during 32ns–55 ns of simulation, with RMSD values of about 0.25 nm.

**FIGURE 10:**
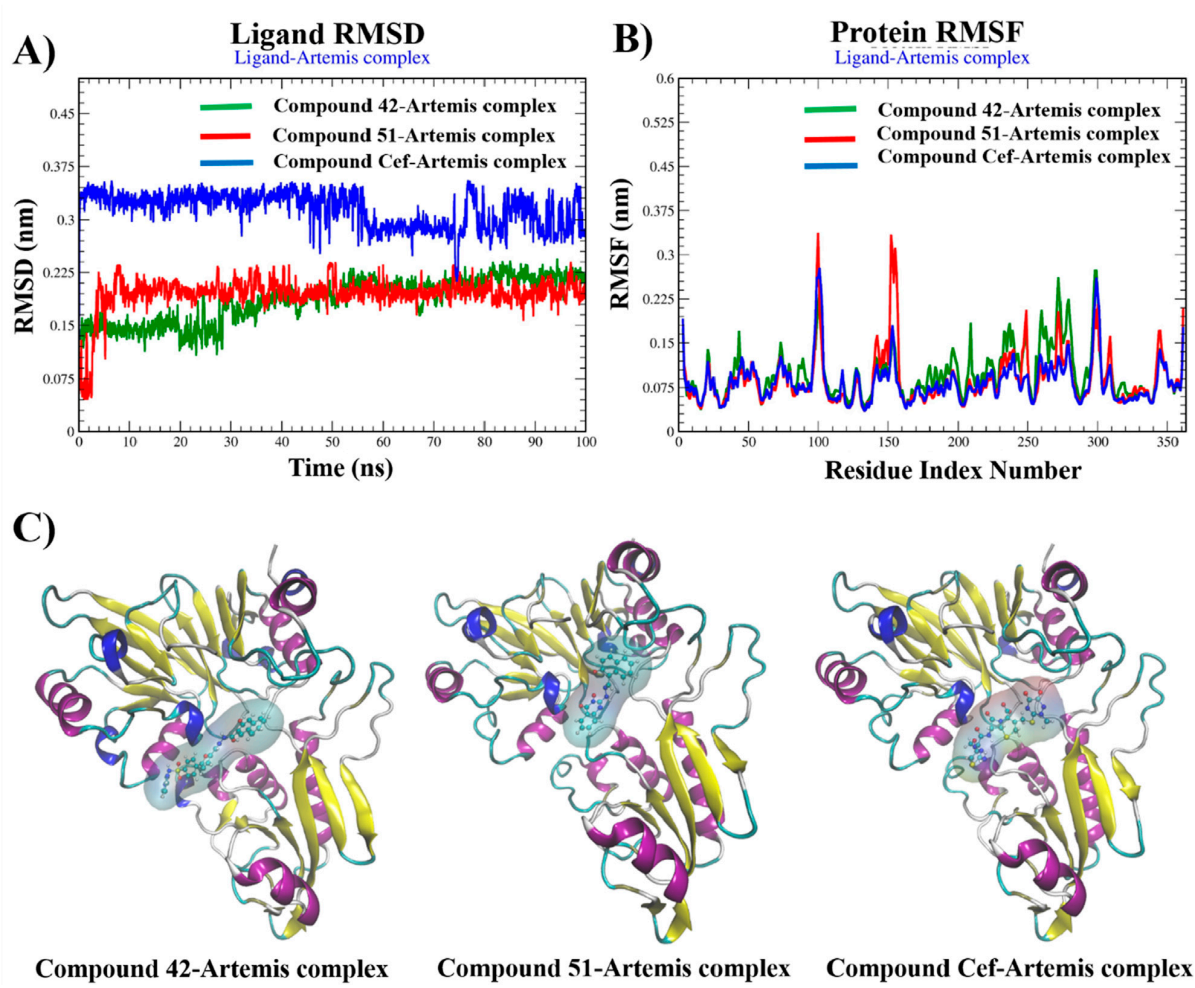
Graphical plots of **(A)** ligand RMSD (nm) versus time (100 ns), **(B)** protein RMSF (nm) versus residue index number, and **(C)** Snapshots of MD-trajectories for compounds 42, 51, and ceftriaxone in complex with Artemis protein.

Figure 10 illustrates the RMSD, RMSF plots, and MD snapshots of top hits, providing valuable insights into the binding stability of these compounds with the target protein. The average RMSD value of 0.35 nm was recorded from 0 ns to 55 ns for ceftriaxone. The structural stability was increased with RMSDs of about 0.3 nm up to 80 of simulation, and fluctuation was observed at the end of the simulation. In contrast, the compounds 42 and 51 were equilibrated at 2 ns and 10 ns of simulation, respectively, and maintained stability, demonstrating minimal deviation in RMSD values (∼2.5 nm) for the rest of the time period. The binding stability was further validated by aligning MD snapshots, representing that compounds remained stably bound to the protein during the simulation.

Protein Root-Mean-Square Fluctuation (RMSF) is invaluable for determining the structural variation of the Artemis protein during complex formation. The RMSF plots revealed the structural stability of the Artemis protein complex, with fluctuations recorded to be less than 2.0 nm for most of the residues. The complex-42 displayed fluctuations greater than 2.0 nm for amino acid residues 100-101, 278-279, and 297–300, representing flexible loops in protein structure. Complex 51 displayed significant variation in amino acid residues such as 100 and 152-156 with RMSF >3.0 nm. However, the key amino acid residues within the binding pocket remained stable in all complexes over 100 ns of the time period and showed RMSF values less than 2.0 nm.

**FIGURE 11:**
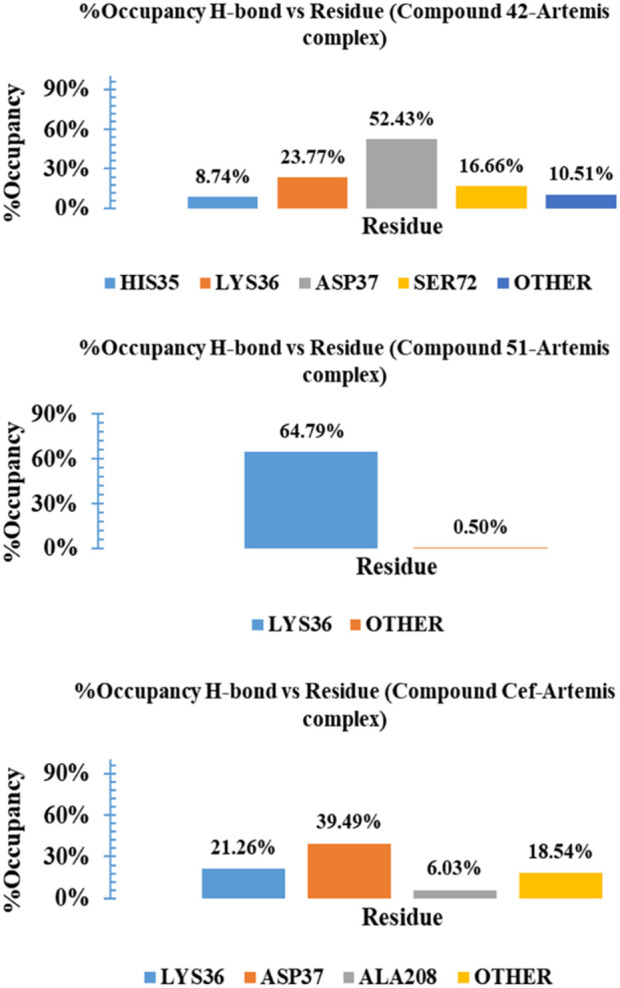
Histogram representation of %occupancies of the H-bond versus residues in protein-ligand contacts for compounds 42, 51, and ceftriaxone in complex with Artemis protein.

The binding stability of complexes relayed on the intermolecular interactions, such as H-bonding with crucial amino acid residues and distortion of these interactions under dynamic conditions, is a key factor of complex instability. Supplementary Figure S12 Illustrates the H-bond interaction analysis of complexes over a 100 ns time period. The complex-42 exhibited 3-4 no. Of H-bonds with three consistent H-bonds for most of the simulation time period. A similar H-bonding pattern is observed for ceftriaxone, reflecting their significant stability during the simulation. Compound 51 displayed only one H-bond number for up to 10 ns and gained stability with two H-bonding interactions for the rest of the simulation. To gain further insights and assess the stability of these interactions, we calculated the percentage occupancies of specific residues involved in H-bonding interactions. Figure 11 presented a histogram of the % occupancy of hydrogen bond contacts formed by compounds 42, 51, and ceftriaxone in complex with Artemis protein. Compound 42 exhibited significant H-bonding interactions among residues ASP37 and LYS36 with occupancy rates of 52.43% and 23.77%, respectively. The H-bonding interaction is also recorded with another catalytic amino acid residue, HIS35, showing 8.74% occupancy. In contrast, compound 51 exhibited H-bonding interaction with LYS36 for most of the simulation time, demonstrating a 64.79% occupancy rate. The reference compound, ceftriaxone, showed 21.26% and 31.29% H-bonding occupancy for LYS36 and ASP37, respectively. These findings concluded the greater effective binding of compound 42, showcasing substantial interaction with catalytic amino acid residues of the target protein.

**FIGURE 12:**
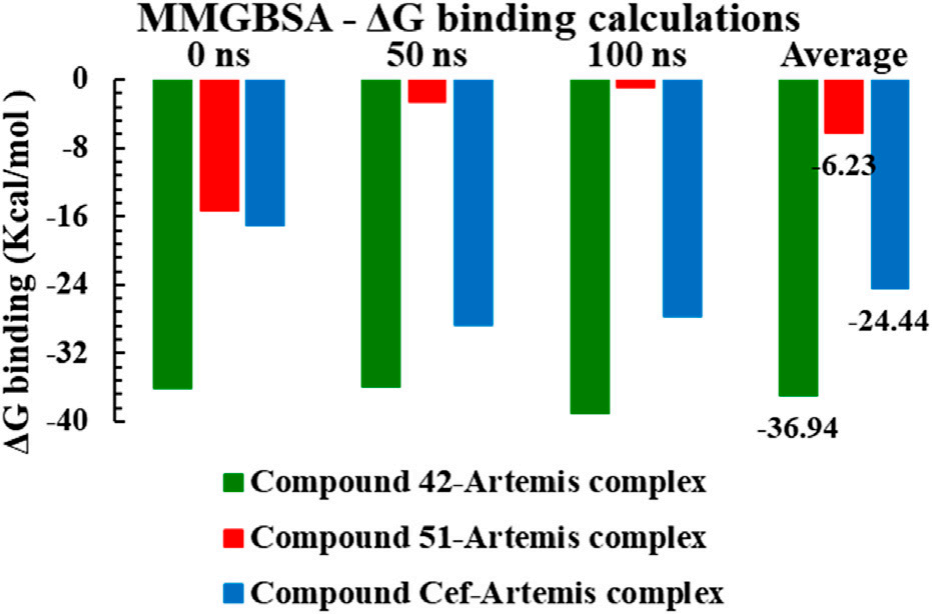
The graphical representation of MMGBSA ΔG binding energy calculations for compounds 42, 51, and ceftriaxone in complex with Artemis protein (PDB ID: 7ABS).

The MM-GBSA method was employed for Gibbs free energy (ΔG) calculations at 0 ns, 50 ns and 100 ns simulation trajectories, tabulated in Supplementary Table S5, to estimate the binding stability of investigated complexes in a dynamic environment. The binding free energy for complex 42 ranged from −38.56 kcal/mol to −37.48 kcal/mol at different simulation trajectories, indicating the complex stability with an average ΔG value of −36.94 kcal/mol. The ceftriaxone complex displayed higher binding affinities for time, maximum at 50 ns of simulation, with an average ΔG value of −24.44 kcal/mol. In contrast, the complex-51 displayed significant binding free energy with ΔG calculated to be −15.58 kcal/mol at 0 ns of trajectory. However, high binding energies of −5.58 kcal/mol and −2.67 kcal/mol were recorded at 50 ns and 100 ns intervals, suggesting that new conformations of compound 51 were found to be energetically less favorable. Figure 12 illustrated the graphical representation of MM-GBSA Gibbs free energy calculations for studied complexes.

### Principle component analysis

Molecular dynamics (MD) simulations are crucial for assessing the flexibility of ligands and receptors. However, the receptor conformations can shift within nanoseconds, so identifying suitable conformations for MD to screen millions of compounds for potential inhibitors with high efficacy poses a significant challenge. By combining these simulations with theoretical models, certain receptor conformations emerge as promising ones, warranting deeper investigation. Despite this, achieving the necessary accuracy in calculations is difficult. The challenge lay in selecting the most promising conformations while managing the long computational times, even though simulations typically span only microseconds (Adcock and McCammon, 2006; Robson, 2022).

To address this, several advanced techniques have been developed to reduce the number of frames needed while retaining critical data. Among these techniques, principal component analysis (PCA) (Das and Mukhopadhyay, 2007), and wavelet analysis (Heidari et al., 2016) were employed to interpret complex data. PCA, in particular, is widely used to simplify the high dimensionality of datasets by focusing on the most relevant principal components. These components capture the key variations in protein motion, enabling the global dynamics of the protein to be represented without losing essential details. PCA reveals important structural variations occurring in the protein’s trajectory by analyzing eigenvalues from the covariance matrix. Through this process, the most dominant protein motions can be identified, highlighting significant conformational changes throughout the simulation (David and Jacobs, 2014).

**FIGURE 13:**
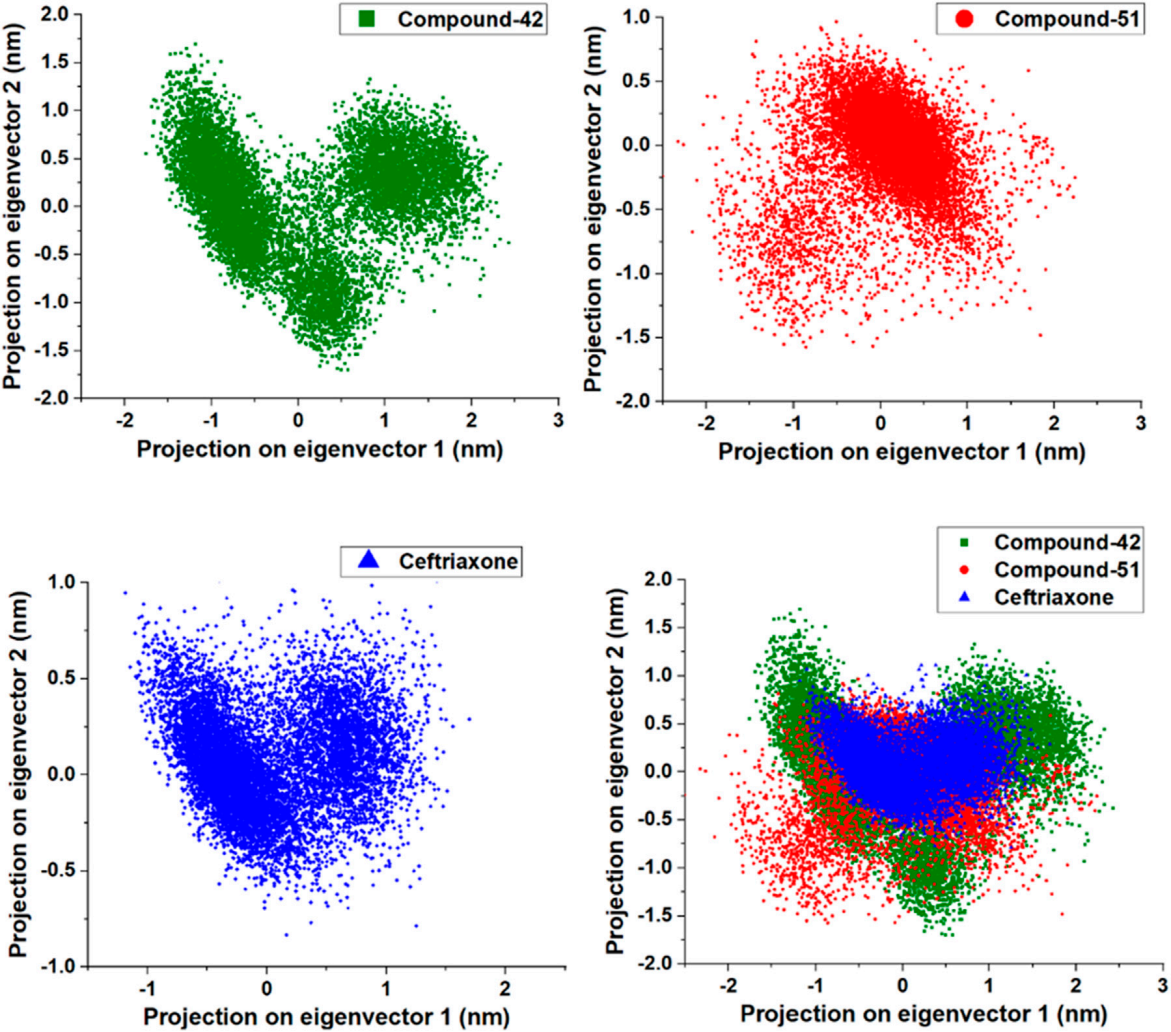
Principal Component analysis for compound-42, compound-51 and cefriaxone in complex with target protein (PDB ID: 7ABS).

This study conducted a two-dimensional (2D) PCA to compare the dynamics of three systems: 7ABS-42 complex, 7ABS-51 complex, and 7ABS-ceftriaxone complex. The goal was to understand how compound-42, compound-51, and ceftriaxone binding influences protein motion. As shown in Figure 13, the binding of compound-42 to 7ABS confined the protein’s motion to a compact phase space with a narrow binding site volume, indicating stronger binding interactions than ceftriaxone. In contrast, the binding of compound-51 led to increased internal motions, resulting in a new configuration with a wider binding site volume. A comparison of the PCA results with the RMSF (Root Mean Square Fluctuations) values for the 7ABS-42 and 7ABS-51 complexes indicated that the 7ABS-51 complex occupied a wider phase space, likely due to higher fluctuations in the 100-150 region. Overall, the PCA results highlighted that the binding site volume decreased in the order of the 7ABS-51 > 7ABS-ceftriaxone > 7ABS-42 complexes, indicating a strong interaction of compound-42 with the target protein.

### Free energy landscape (FEL) analysis

**FIGURE 14:**
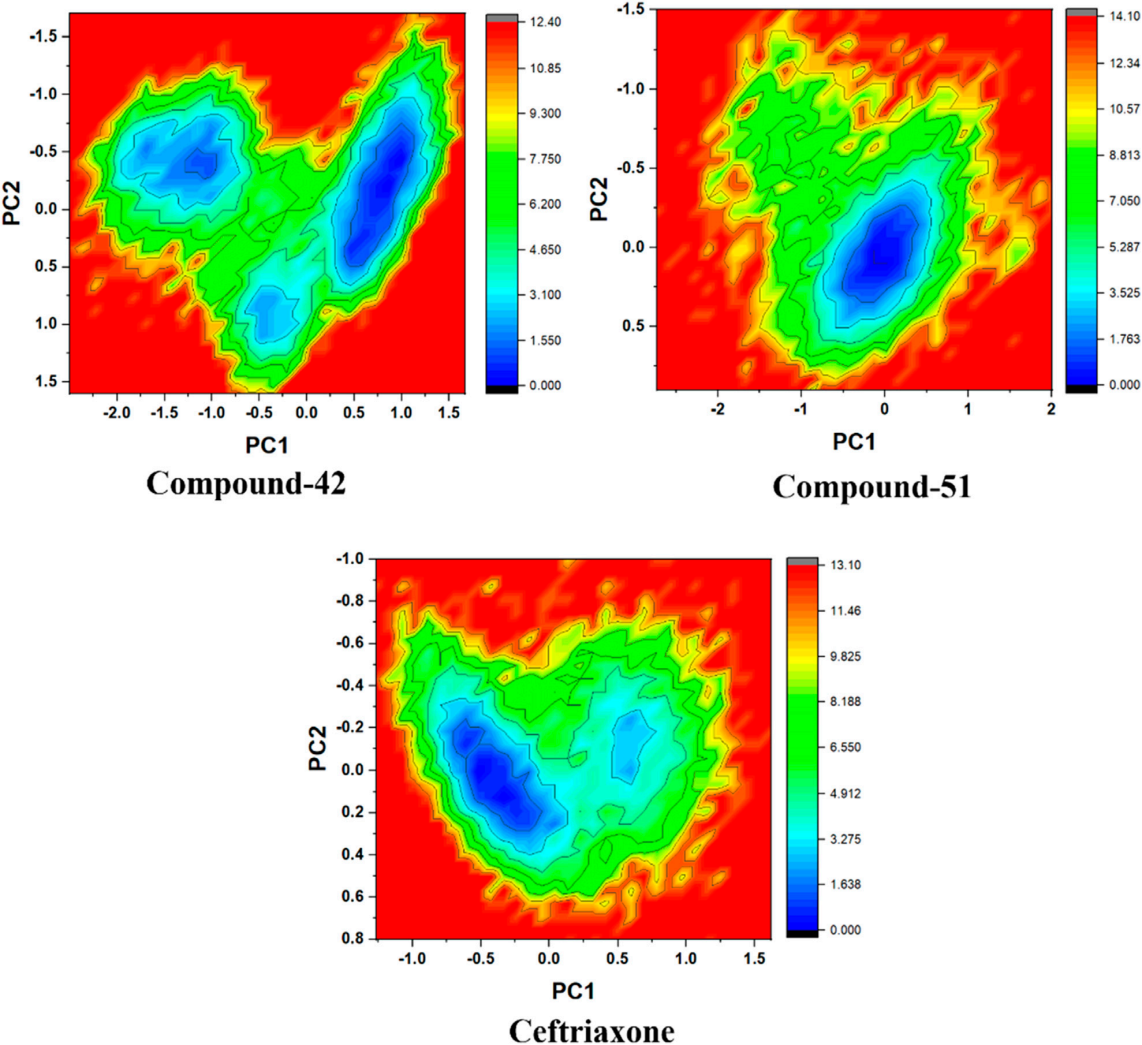
Gibbs free energy landscape for compound-42, compound-51, and cefriaxone in complex with target protein (PDB ID: 7ABS).

Based on PCA, free energy landscape (FEL) analysis provides a more accurate representation of the protein’s conformational space in terms of energy and time. FEL is constructed from the probability distribution of specific data points, which are then transformed into free energy values using a straightforward mathematical relationship (Papaleo et al., 2009). Figure 14 illustrated FEL projections onto the first two principal components for the 7ABS-42, 7ABS-51, and 7ABS-ceftriaxone complexes, focusing on the Cα-atoms of 7ABS. Deep blue areas represented the lowest energy conformations in these plots, while red areas indicated the highest. For the 7ABS-42 complex, a more compact blue region covering a larger surface area is suggested to be a stable cluster. The conformational state with energy minima of varying depths, separated by an energy barrier, indicated that 7ABS-42 could adopt several thermodynamically favorable conformations, allowing for more significant interactions over time.

In contrast, the 7ABS-51 complex displayed a single deep blue region, indicating greater stability with fewer conformational changes than the reference. This is further supported by the 7ABS-51 complex’s low RMSD, which showcases only minor fluctuations throughout the simulation. Meanwhile, the 7ABS-ceftriaxone complex exhibited two distinct blue regions, separated by a small energy barrier, suggesting that it existed in two different energetically favorable conformational states.

The FEL results align well with the hydrogen bonding (H-bonding) analysis. The less conformationally dynamic 7ABS-51 complex consistently maintained two H-bonds throughout the simulation, whereas the 7ABS-ceftriaxone complex displayed up to four H-bonds over time. Similarly, the conformational flexibility of the 7ABS-42 complex resulted in the formation of four H-bonds for most of the simulation period, indicating a more favorable conformational shift than the 7ABS-ceftriaxone complex.

## Conclusion

Our experiments showed that Artemis deficiency in CJ179 cells would leave some DNA breaks unrepaired, as observed by the increase in micronucleus frequency. Overall, Artemis inhibition in most target cell lines should, in principle, radiosensitize them and increase target cell lethality upon irradiation. Experiments with siRNA inhibition of Artemis also exhibited a 40 percent decrease in Artemis gene expression level as compared to control in 1MOS and 1PTNC. In 2PTN, 30 percent, whereas around a 20 percent decrease in 3PRF and 2PRF samples was observed. The increase in DNA PK gene expression shown by IMOS was not significant as compared to 2PRF. whereas other primary cell lines had a downregulation effect. 1PTN showed no decreasing trend as compared to 2PTN and 3PRF, which showed almost up to 15 percent and 30 percent decrease, respectively. Gene expression analysis of TP53 showed that 2PTN had the highest increase in p53 gene expression, which was almost 46%. Almost similar trend was observed in 2PRF, whereas 1PTN and 1MOS showed a similarity in increased expression of 38%. We selected one primary Artemis inhibitor and tested it against 5 primary cell lines derived from invasive ductal carcinomas and found that Artemis inhibition does not affect the DNA repair process, but inhibition results in increased DNA damage as evident from the COMET assay data. This increased DNA damage puts the irradiated cell on a path of apoptosis, notwithstanding the heavy damage. The *in vitro* analysis revealed small molecule (HMAD) as an inhibitor of Artemis protein, further motivating us to conduct an *in silico* screening of the compound library in search of a more potent inhibitor. In the current study, a small molecule library of 69 compounds, retrieved from the ZINC database, was investigated for their inhibiting potential against the endonuclease protein Artemis (PDB ID: 7ABS) to boost the effect of ionizing radiation in cancer therapy. The sixteen compounds were recognized as top hits through docking studies with ∆G-scores greater than −8.0 kcal/mol, which exposed physicochemical and pharmacokinetic properties for further screening. Furthermore, the DFT studies unveiled the electronic properties of top-hit compounds, suggesting the notable reactivity of compounds 42 and 51 with softness values of 0.29 eV and 0.27 eV, respectively. MEP analysis shows these compounds demonstrated the nucleophilic attack sites highlighted with blue color coding, indicating their potential involvement in intermolecular interactions. Based on docking studies along with ADMET and DFT analysis, compounds 42 and 51 were subjected to MD simulation for 100 ns. Compound 42 showed stable trajectories throughout the simulation, evidenced by consistent H-bonding interactions with key amino acid residues. Furthermore, the Gibbs free energies were calculated by the MM-GBSA method, showing higher binding affinities for compound 42 with average ∆G values of −36.94 kcal/mol, suggesting it to be a promising candidate in potentiating the effect of radiotherapy. Furthermore, the PCA and FEL analysesW support the simulation results, indicating the significant potential of these compounds as radiosensitizers in cancer therapy. Future studies could benefit from incorporating MM-PBSA to refine binding energy estimations and to further validate lead compound selection.

## Data Availability

The datasets presented in this study can be found in online repositories. The names of the repository/repositories and accession number(s) can be found in the article/Supplementary Material.

## References

[B1] Abdi H. Williams L. J. (2010). Principal component analysis. Wiley Interdiscip. Rev. Comput. Stat. 2 (4), 433–459. 10.1002/wics.101

[B2] Abel R. Wang L. Harder E. D. Berne B. J. Friesner R. A. (2017). Advancing drug discovery through enhanced free energy calculations. Accounts Chem. Res. 50 (7), 1625–1632. 10.1021/acs.accounts.7b00083 28677954

[B3] Abraham M. J. Murtola T. Schulz R. Páll S. Smith J. C. Hess B. (2022). GROMACS: high performance molecular simulations through multi-level parallelism from laptops to supercomputers. (Elsevier: Netherlands) GROMACS Development Team, 19–25. Available online at: www.gromacs.org.

[B4] Adcock S. A. McCammon J. A. (2006). Molecular dynamics: survey of methods for simulating the activity of proteins. Chem. Rev. 106 (5), 1589–1615. 10.1021/cr040426m 16683746 PMC2547409

[B5] Agoni C. Olotu F. A. Ramharack P. Soliman M. E. (2020). Druggability and drug-likeness concepts in drug design: are biomodelling and predictive tools having their say? J. Mol. Model. 26, 120–11. 10.1007/s00894-020-04385-6 32382800

[B6] Allen W. J. Balius T. E. Mukherjee S. Brozell S. R. Moustakas D. T. Lang P. T. (2015). DOCK 6: impact of new features and current docking performance. J. Comput. Chem. 36 (15), 1132–1156. 10.1002/jcc.23905 25914306 PMC4469538

[B7] Anwar T. Kumar P. Khan A. U. (2021). “Modern tools and techniques in computer-aided drug design,” in Molecular docking for computer-aided drug design (Elsevier), 1–30.

[B8] Arshad M. Ahmed K. Bashir M. Kosar N. Kanwal M. Ahmed M. (2021). Synthesis, structural properties and potent bioactivities supported by molecular docking and DFT studies of new hydrazones derived from 5-chloroisatin and 2-thiophenecarboxaldehyde. J. Mol. Struct. 1246, 131204. 10.1016/j.molstruc.2021.131204

[B9] Aziz M. Ejaz S. A. Rehman H. M. Alsubaie A. S. Mahmoud K. H. Siddique F. (2023). Identification of NEK7 inhibitors: structure based virtual screening, molecular docking, density functional theory calculations and molecular dynamics simulations. J. Biomol. Struct. Dyn. 41 (14), 6894–6908. 10.1080/07391102.2022.2113563 35983608

[B10] Aziz M. Ejaz S. A. Tamam N. Siddique F. Riaz N. Qais F. A. (2022b). Identification of potent inhibitors of NEK7 protein using a comprehensive computational approach. Sci. Rep. 12 (1), 6404. 10.1038/s41598-022-10253-5 35436996 PMC9016071

[B11] Aziz M. Ejaz S. A. Zargar S. Akhtar N. Aborode A. T. A. Wani T. (2022a). Deep learning and structure-based virtual screening for drug discovery against NEK7: a novel target for the treatment of cancer. Molecules 27 (13), 4098. 10.3390/molecules27134098 35807344 PMC9268522

[B12] Badar M. S. Shamsi S. Ahmed J. Alam A. (2022). “Molecular dynamics simulations: concept, methods, and applications,” in Transdisciplinarity (Springer), 131–151.

[B13] Becke A. D. (1993). Density‐functional thermochemistry. III. The role of exact exchange. J. Chem. Phys. 98 (7), 5648–5652. 10.1063/1.464913

[B14] Bilal M. S. Ejaz S. A. Zargar S. Akhtar N. Wani T. A. Riaz N. (2022). Computational investigation of 1, 3, 4 oxadiazole derivatives as lead inhibitors of VEGFR 2 in comparison with EGFR: density functional theory, molecular docking and molecular dynamics simulation studies. Biomolecules 12 (11), 1612. 10.3390/biom12111612 36358960 PMC9687636

[B15] Burdak-Rothkamm S. Rothkamm K. Prise K. M. (2008). ATM acts downstream of ATR in the DNA damage response signaling of bystander cells. Cancer Res. 68 (17), 7059–7065. 10.1158/0008-5472.can-08-0545 18757420 PMC2528059

[B16] Chattaraj P. Nath S. Maiti B. (2003). Reactivity descriptors. Computational medicinal chemistry for drug discovery. 11, 295.

[B17] Clark T. Chandrasekhar J. Spitznagel G. W. Schleyer P. V. R. (1983). Efficient diffuse function-augmented basis sets for anion calculations. III. The 3-21+G basis set for first-row elements, Li–F. J. Comput. Chem. 4 (3), 294–301. 10.1002/jcc.540040303

[B18] Cross S. S. (2005). Improved FlexX docking using FlexS-determined base fragment placement. J. Chem. Inf. Model. 45 (4), 993–1001. 10.1021/ci050026f 16045293

[B19] Das A. Mukhopadhyay C. (2007). Application of principal component analysis in protein unfolding: an all-atom molecular dynamics simulation study. J. Chem. Phys. 127 (16), 165103. 10.1063/1.2796165 17979396

[B20] David C. C. Jacobs D. J. (2014). Principal component analysis: a method for determining the essential dynamics of proteins. Protein Dyn. Methods Protoc. 1084, 193–226. 10.1007/978-1-62703-658-0_11 PMC4676806 24061923

[B21] Ejaz S. A. Aziz M. Ahmed A. Alotaibi S. S. Albogami S. M. Siddique F. (2023). New Insight into the pharmacological importance of atropine as the potential inhibitor of AKR1B1 via detailed computational investigations: DFTs, ADMET, molecular docking, and molecular dynamics studies. Appl. Biochem. Biotechnol. 195 (8), 5136–5157. 10.1007/s12010-023-04411-2 36847982

[B22] Farhan S. Aqdas A. Bashir M. Nadeem S. Rawat R. (2024). Harnessing the potential of natural products in cancer treatment: a comprehensive review. J. Biol. Regul. Homeost. Agents 38 (2), 873–897. 10.23812/j.biol.regul.homeost.agents.20243802.72

[B23] Friesner R. A. Banks J. L. Murphy R. B. Halgren T. A. Klicic J. J. Mainz D. T. (2004). Glide: a new approach for rapid, accurate docking and scoring. 1. Method and assessment of docking accuracy. J. Med. Chem. 47 (7), 1739–1749. 10.1021/jm0306430 15027865

[B24] Frisch M. J. Trucks G. W. Schlegel H. B. (2009). Gaussian 09, revision A.02. Wallingford, CT: Gaussian, Inc.

[B25] Glorieux M. (2020). Combining radiotherapy with DNA repair inhibitors as novel treatment strategy for head and neck cancers. KU Leuven. London, United Kingdom: Nature Portfolio.

[B26] Heidari Z. Roe D. R. Galindo-Murillo R. Ghasemi J. B. Cheatham T. E. III (2016). Using wavelet analysis to assist in identification of significant events in molecular dynamics simulations. J. Chem. Inf. Model. 56 (7), 1282–1291. 10.1021/acs.jcim.5b00727 27286268

[B27] Huang S.-Y. Zou X. (2010). Advances and challenges in protein-ligand docking. Int. J. Mol. Sci. 11 (8), 3016–3034. 10.3390/ijms11083016 21152288 PMC2996748

[B28] Huey R. Morris G. M. Olson A. J. Goodsell D. S. (2007). A semiempirical free energy force field with charge‐based desolvation. J. Comput. Chem. 28 (6), 1145–1152. 10.1002/jcc.20634 17274016

[B29] Humphrey W. Dalke A. Schulten K. (1996). VMD: visual molecular dynamics. J. Mol. Graph. 14 (1), 33–38. 10.1016/0263-7855(96)00018-5 8744570

[B30] Irwin J. J. Shoichet B. K. (2005). ZINC− a free database of commercially available compounds for virtual screening. J. Chem. Inf. Model. 45 (1), 177–182. 10.1021/ci049714+ 15667143 PMC1360656

[B31] Jorgensen W. L. Chandrasekhar J. Madura J. D. Impey R. W. Klein M. L. (1983). Comparison of simple potential functions for simulating liquid water. J. Chem. Phys. 79 (2), 926–935. 10.1063/1.445869

[B32] Karim M. F. Liu S. Laciak A. R. Volk L. Koszelak-Rosenblum M. Lieber M. R. (2020). Structural analysis of the catalytic domain of Artemis endonuclease/SNM1C reveals distinct structural features. J. Biol. Chem. 295 (35), 12368–12377. 10.1074/jbc.ra120.014136 32576658 PMC7458816

[B33] Krempler A. (2004). A pathway of double-strand break rejoining dependent upon ATM. Artemis. Cambridge, MA: Cell Press. 10.1016/j.molcel.2004.10.029 15574327

[B34] Kunnakkattu I. R. Choudhary P. Pravda L. Nadzirin N. Smart O. S. Yuan Q. (2023). PDBe CCDUtils: an RDKit-based toolkit for handling and analysing small molecules in the Protein Data Bank. J. Cheminformatics 15 (1), 117. 10.1186/s13321-023-00786-w PMC10693035 38042830

[B35] Li S. Chang H. H. Niewolik D. Hedrick M. P. Pinkerton A. B. Hassig C. A. (2014). Evidence that the DNA endonuclease ARTEMIS also has intrinsic 5′-exonuclease activity. J. Biol. Chem. 289 (11), 7825–7834. 10.1074/jbc.m113.544874 24500713 PMC3953294

[B36] Li Z. Huang Y. Wu Y. Chen J. Wu D. Zhan C. G. (2019). Absolute binding free energy calculation and design of a subnanomolar inhibitor of phosphodiesterase-10. J. Med. Chem. 62 (4), 2099–2111. 10.1021/acs.jmedchem.8b01763 30689375

[B37] Lindahl E. Hess B. Van Der Spoel D. (2001). GROMACS 3.0: a package for molecular simulation and trajectory analysis. Mol. Model. Annu. 7, 306–317. 10.1007/s008940100045

[B38] Liu H. Wang X. Huang A. Gao H. Sun Y. Jiang T. (2018). Silencing artemis enhances colorectal cancer cell sensitivity to DNA-damaging agents. Oncol. Res. 27 (1), 29–38. 10.3727/096504018x15179694020751 29426373 PMC7848410

[B39] Ma Y. Schwarz K. Lieber M. R. (2005). The Artemis: DNA-PKcs endonuclease cleaves DNA loops, flaps, and gaps. DNA repair 4 (7), 845–851. 10.1016/j.dnarep.2005.04.013 15936993

[B40] MacKerell A. D. Jr. Bashford D. Bellott M. Dunbrack R. L. Jr. Evanseck J. D. Field M. J. (1998). All-Atom empirical potential for molecular modeling and dynamics studies of proteins. J. Phys. Chem. B 102 (18), 3586–3616. 10.1021/jp973084f 24889800

[B41] Melenotte C. Pontarotti P. Pinault L. Mège J. L. Devaux C. Raoult D. (2021). Could β-lactam antibiotics block humoral immunity? Front. Immunol. 12, 680146. 10.3389/fimmu.2021.680146 34603278 PMC8480522

[B42] Pagadala N. S. Syed K. Tuszynski J. (2017). Software for molecular docking: a review. Biophys. Rev. 9, 91–102. 10.1007/s12551-016-0247-1 28510083 PMC5425816

[B43] Papaleo E. Mereghetti P. Fantucci P. Grandori R. De Gioia L. (2009). Free-energy landscape, principal component analysis, and structural clustering to identify representative conformations from molecular dynamics simulations: the myoglobin case. J. Mol. Graph. Model. 27 (8), 889–899. 10.1016/j.jmgm.2009.01.006 19264523

[B44] Podrimaj-Bytyqi A. Borovečki A. Selimi Q. Manxhuka-Kerliu S. Gashi G. Elezaj I. R. (2018). The frequencies of micronuclei, nucleoplasmic bridges and nuclear buds as biomarkers of genomic instability in patients with urothelial cell carcinoma. Sci. Rep. 8 (1), 17873. 10.1038/s41598-018-35903-5 30552338 PMC6294807

[B45] Potdar P. Chaugule S. (2011). Establishment and molecular characterization of breast cancer mesenchymal stem cell line derived from human non-metastasis breast cancer tumor. Stem Cell Discov. 1 (02), 21–28. 10.4236/scd.2011.12003

[B46] Prieto-Martínez F. D. López-López E. Juárez-Mercado K. E. Medina-Franco J. L. (2019). “Computational drug design methods—current and future perspectives,” in In silico drug design, 19–44.

[B47] RCSB (2025). Redesigned PDB statistics support enhanced functionality. Available online at: https://www.rcsb.org/.

[B48] Reimers J. R. Cai Z. Bilić A. Hush N. S. (2003). The appropriateness of density‐functional theory for the calculation of molecular electronics properties. Ann. N. Y. Acad. Sci. 1006 (1), 235–251. 10.1196/annals.1292.017 14976022

[B49] Riballo E. Kühne M. Rief N. Doherty A. Smith G. C. Recio M. J. (2004). A pathway of double-strand break rejoining dependent upon ATM, Artemis, and proteins locating to γ-H2AX foci. Mol. cell 16 (5), 715–724. 10.1016/j.molcel.2004.10.029 15574327

[B50] Robson B. (2022). *De novo* protein folding on computers. Benefits and challenges. Comput. Biol. Med. 143, 105292. 10.1016/j.compbiomed.2022.105292 35158120

[B51] Sambrook J. Fritsch E. F. Maniatis T. (1989). Molecular cloning: a laboratory manual.

[B52] Schmidtke P. Barril X. (2010). Understanding and predicting druggability. A high-throughput method for detection of drug binding sites. J. Med. Chem. 53 (15), 5858–5867. 10.1021/jm100574m 20684613

[B53] Schulz-Gasch T. Stahl M. (2004). Scoring functions for protein–ligand interactions: a critical perspective. Drug Discov. Today Technol. 1 (3), 231–239. 10.1016/j.ddtec.2004.08.004 24981490

[B54] Siddique F. Anwaar A. Bashir M. Nadeem S. Rawat R. Eyupoglu V. (2024). Revisiting methotrexate and phototrexate Zinc15 library-based derivatives using deep learning in-silico drug design approach. Front. Chem. 12, 1380266. 10.3389/fchem.2024.1380266 38576849 PMC10991842

[B55] Soubeyrand S. Pope L. De Chasseval R. Gosselin D. Dong F. de Villartay J. P. (2006). Artemis phosphorylated by DNA-dependent protein kinase associates preferentially with discrete regions of chromatin. J. Mol. Biol. 358 (5), 1200–1211. 10.1016/j.jmb.2006.02.061 16600297

[B56] Sun H. Li Y. Shen M. Tian S. Xu L. Pan P. (2014). Assessing the performance of MM/PBSA and MM/GBSA methods. 5. Improved docking performance using high solute dielectric constant MM/GBSA and MM/PBSA rescoring. Phys. Chem. Chem. Phys. 16 (40), 22035–22045. 10.1039/c4cp03179b 25205360

[B57] Tandon H. Chakraborty T. Suhag V. (2019). A brief review on importance of DFT in drug design. Res. Med. Eng. Sci. 7 (4), 791–795. 10.31031/RMES.2019.07.00068

[B58] Tirado-Rives J. Jorgensen W. L. (2008). Performance of B3LYP density functional methods for a large set of organic molecules. J. Chem. theory Comput. 4 (2), 297–306. 10.1021/ct700248k 26620661

[B59] Tuma C. Boese A. D. Handy N. C. (1999). Predicting the binding energies of H-bonded complexes: a comparative DFT study. Phys. Chem. Chem. Phys. 1 (17), 3939–3947. 10.1039/a904357h

[B60] Turner P. (2005). XMGRACE, version 5.1. 19. Center for coastal and land-margin research, Oregon graduate Institute of science and technology. Beavert. OR 2 (5), 19.

[B61] Valencia E. Olarte W. Sastoque F. (2023). *In silico* study of the admet properties of potential inhibitors of new Delhi methalo-ß-lactamase-1 (NDM-1). Seven Ed. São José dos Pinhais, Brazil: part of Seven Publicações Ltda. 10.56238/uniknowindevolp-013

[B62] van de Kamp G. Heemskerk T. Kanaar R. Essers J. (2021). DNA double strand break repair pathways in response to different types of ionizing radiation. Front. Genet. 12, 738230. 10.3389/fgene.2021.738230 34659358 PMC8514742

[B63] Verdonk M. L. Cole J. C. Hartshorn M. J. Murray C. W. Taylor R. D. (2003). Improved protein–ligand docking using GOLD. Proteins Struct. Funct. Bioinforma. 52 (4), 609–623. 10.1002/prot.10465 12910460

[B64] Vogt M. Bajorath J. (2017). Modeling tanimoto similarity value distributions and predicting search results. Mol. Inf. 36 (7), 1600131. 10.1002/minf.201600131 28032955

[B65] Watanabe G. Lieber M. R. Williams D. R. (2022). Structural analysis of the basal state of the Artemis: DNA-PKcs complex. Nucleic Acids Res. 50 (13), 7697–7720. 10.1093/nar/gkac564 35801871 PMC9303282

[B75] Xiong G. Wu Z. Yi J. Fu L. Yang Z. Hsieh C. (2021). ADMETlab 2.0: an integrated online platform for accurate and comprehensive predictions of ADMET properties. Nucleic acids Res. 49 (W1), W5–W14. 10.1093/nar/gkab255 33893803 PMC8262709

[B66] Węglarz L. Molin I. Orchel A. Parfiniewicz B. Dzierżewicz Z. (2006). Quantitative analysis of the level of p53 and p21WAF1 mRNA in human colon cancer HT-29 cells treated with inositol hexaphosphate. 16733561

[B67] Xiong X. Du Z. Wang Y. Feng Z. Fan P. Yan C. (2015). 53BP1 promotes microhomology-mediated end-joining in G1-phase cells. Nucleic acids Res. 43 (3), 1659–1670. 10.1093/nar/gku1406 25586219 PMC4330367

[B68] Yang X. Wang Y. Byrne R. Schneider G. Yang S. (2019). Concepts of artificial intelligence for computer-assisted drug discovery. Chem. Rev. 119 (18), 10520–10594. 10.1021/acs.chemrev.8b00728 31294972

[B69] Yaqoob F. Aftab H. Sadeghian N. Taslimi P. Siddique F. Nadeem S. (2025). Design, synthesis, antidiabetic evaluation and computational modeling of phenylnaphthalene-2-sulfonate derived hydrazones. J. Mol. Struct. 1335, 141883. 10.1016/j.molstruc.2025.141883

[B76] Yasaei H. Slijepcevic P. (2010). Research Defective Artemis causes mild telomere dysfunction. 10.1186/2041-9414-1-3 PMC2907561 20678254

[B70] Yosaatmadja Y. Baddock H. Newman J. Bielinski M. Gavard A. Mukhopadhyay S. M. M. (2021). Structural and mechanistic insights into the Artemis endonuclease and strategies for its inhibition. Nucleic acids Res. 49 (16), 9310–9326. 10.1093/nar/gkab693 34387696 PMC8450076

[B71] Zadorozhnii P. V. Kiselev V. V. Kharchenko A. V. (2022). *In silico* ADME profiling of salubrinal and its analogues. Future Pharmacol. 2 (2), 160–197. 10.3390/futurepharmacol2020013

[B72] Zang Q. Mansouri K. Williams A. J. Judson R. S. Allen D. G. Casey W. M. (2017). *In silico* prediction of physicochemical properties of environmental chemicals using molecular fingerprints and machine learning. J. Chem. Inf. Model. 57 (1), 36–49. 10.1021/acs.jcim.6b00625 28006899 PMC6131700

[B73] Zhang X. Succi J. Feng Z. Prithivirajsingh S. Story M. D. Legerski R. J. (2004). Artemis is a phosphorylation target of ATM and ATR and is involved in the G2/M DNA damage checkpoint response. Mol. Cell. Biol. 24, 9207–9220. 10.1128/mcb.24.20.9207-9220.2004 15456891 PMC517881

[B77] Zhang X. Zhu Y. Geng L. Wang H. Legerski R. J. (2009). Artemis is a negative regulator of p53 in response to oxidative stress. Oncogene 28 (22), 2196–2204. 19398950 10.1038/onc.2009.100 PMC2692457

[B74] Zoete V. Grosdidier A. Michielin O. (2009). Docking, virtual high throughput screening and *in silico* fragment‐based drug design. J. Cell. Mol. Med. 13 (2), 238–248. 10.1111/j.1582-4934.2008.00665.x 19183238 PMC3823351

