# Supplementary material for: Computational advances in the design and discovery of artemis inhibitors for radiosensitization in cancer therapy
Source: Front Chem. 2025 Jul 28;13:1597454. doi: 10.3389/fchem.2025.1597454 (PMC12336226; doi:10.3389/fchem.2025.1597454)
Supplement: Supplementary file 1 [file DataSheet1.docx]

**Supplementary information**

**Computational Advances in the Design and Discovery of Artemis Inhibitors for Radiosensitization in Cancer Therapy**

**Contents**

[**Table S1:** The IUPAC names and chemical structures of 69 compounds. 4](#_Toc184057654)

[**Table S2:** The SMILES name, G-score and E-model scores for best conformational pose of each ligand docked with Artemis (PDB ID: 7ABS). 24](#_Toc184057656)

[**Table S3:** The ADMET properties including physicochemical parameters, absorption, distribution and bioavailability of investigated compounds. 28](#_Toc184057657)

[**Table S4:** The ADMET properties including Blood brain penetration, metabolism and excretion in terms of half-life of investigated ligands. 30](#_Toc184057658)

[**Table S5:** MMGBSA ΔG binding energy calculations for compounds 42, 51, and ceftriaxone in complex with Artemis protein (PDB ID: 7ABS). 31](#_Toc184057659)

[**Figure S1:** 2D and 3D interaction of investigated ligands 01, 02 and 04 with Artemis protein (PDB ID: 7ABS). 33](#_Toc184057660)

[**Figure S2:** 2D and 3D interaction of investigated ligands 05, 42 and 43 with Artemis protein (PDB ID: 7ABS). 34](#_Toc184057661)

[**Figure S3:** 2D and 3D interaction of investigated ligands 44, 46 and 51 with Artemis protein (PDB ID: 7ABS). 35](#_Toc184057662)

[**Figure S4:** 2D and 3D interaction of investigated ligands 58 and ceftriaxone with Artemis protein (PDB ID: 7ABS). 36](#_Toc184057663)

[**Figure S5:** Optimized structures of the investigated compounds at B3LYP/6-311+g(d, p) level of DFT calculations in the gas phase. 37](#_Toc184057664)

[**Figure S6:** The contour plots of HOMOs and LUMOs for compounds 01, 02, 04, 05 and 23. 38](#_Toc184057665)

[**Figure S7:** The contour plots of HOMOs and LUMOs for compounds 28, 42 and 43. 38](#_Toc184057666)

[**Figure S8:** The contour plots of HOMOs and LUMOs for compounds 44, 46, 49 and 51. 39](#_Toc184057667)

[**Figure S9:** The contour plots of HOMOs and LUMOs for compounds 55, 58, 59, 67 and ceftriaxone. 39](#_Toc184057668)

[Figure S10: Molecular Electrostatic Potential (MEP) maps for the investigated compounds. 40](#_Toc184057669)

[**Figure S11:** Graphical plots of Artemis-protein RMSD (nm) versus time (100 ns) for compounds 42, 51, and ceftriaxone in complex with the target protein. 41](#_Toc184057670)

[**Figure S12:** Graphical representation of the number of H-bond contacts formed by compounds 42, 51, and ceftriaxone in complex with Artemis protein. 42](#_Toc184057671)

**Table S1:** The IUPAC names and chemical structures of 69 compounds.

| **Compounds** | **IUPAC Name** | **Structure** |
| --- | --- | --- |
| **1** | 4-hydroxy-N-(3-hydroxypyridin-2-yl)-1-isobutyl-2-oxo-1,2,5,6,7,8-hexahydroquinoline-3-carboxamide |  |
| **2** | N-(1H-benzo[d]imidazol-2-yl)-1-ethyl-4-hydroxy-2-oxo-1,2-dihydroquinoline-3-carboxamide |  |
| **3** | 1-((Z)-(((1S,3S,8S)-1,3,6-triazatricyclo[4.3.1.13,8]undecan-8-yl)imino)methyl)naphthalen-2-ol |  |
| **4** | (E)-2-(1-(hydroxyimino)ethyl)benzoic acid |  |
| **5** | 2-hydroxy-6-(piperidine-1-carbonyl)-1H-benzo[de]isoquinoline-1,3(2H)-dione |  |
| **6** | 1-(furan-2-ylmethyl)-2-imino-N-(4-methylbenzyl)-5-oxo-1,5-dihydro-2H-dipyrido[1,2-a:2',3'-d]pyrimidine-3-carboxamide |  |
| **7** | N-(furan-2-ylmethyl)-2-(pyridin-2-yl)quinazolin-4-amine |  |
| **8** | (E)-3-(2-(cyclohex-1-en-1-yl)ethyl)-11-((furan-2-ylmethylene)amino)-2-methyl-3,11-dihydro-4H-pyrimido[5',4':4,5]pyrrolo[2,3-b]quinoxalin-4-one |  |
| **9** | N-(5-(4-methoxyphenyl)-7-phenyl-4,5,6,7-tetrahydro-[1,2,4]triazolo[1,5-a]pyrimidin-2-yl)thiophene-2-carboxamide |  |
| **10** | 2-(3,5-dimethyl-1H-pyrazol-1-yl)-N-(2-methoxyethyl)-5,6,7,8-tetrahydrobenzo[4,5]thieno[2,3-d]pyrimidin-4-amine |  |
| **11** | 5-benzyl-8-methyl-3-(methylthio)-5H-[1,2,4]triazino[5,6-b]indole |  |
| **12** | 4-(azepan-1-yl)-2-(pyridin-2-yl)quinazoline |  |
| **13** | N-(5-(4-chlorophenyl)-7-phenyl-4,5,6,7-tetrahydro-[1,2,4]triazolo[1,5-a]pyrimidin-2-yl)furan-2-carboxamide |  |
| **14** | 6-(3-methylbenzofuran-2-yl)-N2-phenyl-1,3,5-triazine-2,4-diamine |  |
| **15** | N-hydroxy-2-(2-phenylthiophen-3-yl)acetamide |  |
| **16** | 7-(pyridin-2-yl(pyridin-2-ylamino)methyl)quinolin-8-ol |  |
| **17** | 4-(4-chloro-2-methylphenoxy)-N-hydroxybutanamide |  |
| **18** | 2-(pyridin-2-yl)-3H-naphtho[1,2-d]imidazole |  |
| **19** | 4,6-bis(3,5-dimethyl-1H-pyrazol-1-yl)-N-(2-(trifluoromethyl)phenyl)-1,3,5-triazin-2-amine |  |
| **20** | 2-(phenylamino)-3-(piperidin-1-yl)naphthalene-1,4-dione |  |
| **21** | 2-(pyridin-2-yl)-1H-benzo[d]imidazol-5-amine |  |
| **22** | 2-((3-chlorobenzyl)oxy)isoindoline-1,3-dione |  |
| **23** | 1-(4-(2-(pyridin-2-yl)pyrimidin-4-yl)phenyl)-1H-benzo[d]imidazole |  |
| **24** | 2-aminophenanthrene-9,10-dione |  |
| **25** | 6-((4,5-dihydrothiazol-2-yl)thio)-N2,N4-bis(4-methoxyphenyl)-1,3,5-triazine-2,4-diamine |  |
| **26** | (E)-1-(1-(4-((2,4-dichlorobenzyl)oxy)phenyl)-2,5-dimethyl-1H-pyrrol-3-yl)-N-(4H-1,2,4-triazol-4-yl)methanimine |  |
| **27** | 3-(4-methoxyphenyl)-1-phenyl-N-(thiazol-2-yl)-1H-pyrazole-4-carboxamide |  |
| **28** | ethyl 4-((3,4-dioxo-3,4-dihydronaphthalen-1-yl)amino)benzoate |  |
| **29** | (E)-1-(1-(4-((4-chlorobenzyl)oxy)phenyl)-2,5-dimethyl-1H-pyrrol-3-yl)-N-(4H-1,2,4-triazol-4-yl)methanimine |  |
| **30** | 4-methoxy-N-(4-(pyrrolidin-1-yl)phenyl)benzamide |  |
| **31** | 2-phenyl-N-(4-(pyrrolidin-1-yl)phenyl)acetamide |  |
| **32** | (E)-1-(pyridin-2-yl)decan-1-one oxime |  |
| **33** | 2-(1H-benzo[d][1,2,3]triazol-1-yl)-3-morpholinonaphthalene-1,4-dione |  |
| **34** | 3-benzoyl-2-methylnaphtho[1,2-b]furan-4,5-dione |  |
| **35** | 2-methyl-3-(4-methylbenzoyl)naphtho[1,2-b]furan-4,5-dione |  |
| **36** | 3-(4-chlorobenzoyl)-2-methylnaphtho[1,2-b]furan-4,5-dione |  |
| **37** | (Z)-N'-(3-oxobenzo[b]thiophen-2(3H)-ylidene)-N-(m-tolyl)acetohydrazide |  |
| **38** | (Z)-N'-(3-oxobenzo[b]thiophen-2(3H)-ylidene)-N-(o-tolyl)acetohydrazide |  |
| **39** | (Z)-N'-(3-oxobenzo[b]thiophen-2(3H)-ylidene)-N-phenylacetohydrazide |  |
| **40** | N-(5-ethyl-4,5,6,7-tetrahydrothiazolo[5,4-c]pyridin-2-yl)-3,3-diphenylpropanamide |  |
| **41** | 4-(phenylthio)-N-(4-(pyridin-2-yl)thiazol-2-yl)butanamide |  |
| **42** | (E)-4-(5-((2-(3-hydroxy-2-naphthoyl)hydrazineylidene)methyl)furan-2-yl)-N-(pyrimidin-2-yl)benzenesulfonamide |  |
| **43** | (E)-2-((2-(4-(3,5-dimethyl-1H-pyrazol-1-yl)-6-(phenylamino)-1,3,5-triazin-2-yl)hydrazineylidene)methyl)-6-methoxyphenol |  |
| **44** | (E)-2-amino-3-((4-chlorophenyl)diazenyl)-5,6,7,8-tetrahydropyrazolo[5,1-b]quinazolin-9-ol |  |
| **45** | (E)-2-((2-(1H-benzo[d]imidazol-2-yl)hydrazineylidene)methyl)-5-(diethylamino)phenol |  |
| **46** | 5-bromo-3-cinnamoyl-2-hydroxycyclohepta-2,4,6-trien-1-one |  |
| **47** | 3-methyl-N-(4-methyl-5-(phenylcarbamoyl)thiazol-2-yl)-1-phenyl-1H-thieno[2,3-c]pyrazole-5-carboxamide |  |
| **48** | (E)-6-bromo-2-(2-((5-nitrofuran-2-yl)methylene)hydrazineyl)-4-phenylquinazoline |  |
| **49** | N-(2-(naphthalen-1-yl)benzo[d]oxazol-5-yl)-1H-1,2,4-triazole-3-carboxamide |  |
| **50** | (E)-2-((2-(4-(3,5-dimethyl-1H-pyrazol-1-yl)-6-(phenylamino)-1,3,5-triazin-2-yl)hydrazineylidene)methyl)-4-methoxyphenol |  |
| **51** | (E)-3-hydroxy-2-(2-((2-hydroxynaphthalen-1-yl)methylene)hydrazineyl)quinazolin-4(3H)-one |  |
| **52** | (E)-6-(2-benzylidenehydrazineyl)-N2-(furan-2-ylmethyl)-N4-(4-methoxyphenyl)-1,3,5-triazineN2,N4-di-tert-butyl-6-hydrazineyl-1,3,5-triazine-2,4-diamine2,4-diamine |  |
| **53** | N2,N4-di-tert-butyl-6-hydrazineyl-1,3,5-triazine-2,4-diamine |  |
| **54** | 4,4'-((1-(naphthalen-1-ylmethyl)-1H-indol-3-yl)methylene)bis(3-methyl-1H-pyrazol-5-ol) |  |
| **55** | 2-amino-3-((3-methoxyphenyl)diazenyl)-5-phenylpyrazolo[1,5-a]pyrimidin-7-ol |  |
| **56** | (E)-2-amino-5-phenyl-3-(phenyldiazenyl)pyrazolo[1,5-a]pyrimidin-7-ol |  |
| **57** | (E)-2-amino-5-phenyl-3-(p-tolyldiazenyl)pyrazolo[1,5-a]pyrimidin-7-ol |  |
| **58** | N'-(acridin-9-yl)-2,4-dihydroxybenzohydrazide |  |
| **59** | N'-((Z)-3,4-dihydroxybenzylidene)-2-(2-(2-((E)-3,4-dihydroxybenzylidene)hydrazineyl)-2-oxoethoxy)benzohydrazide |  |
| **60** | (Z)-2-(ethylthio)-4-((5-(2-nitrophenyl)furan-2-yl)methylene)thiazol-5(4H)-one |  |
| **61** | N-(3-(1H-imidazol-1-yl)propyl)-7-(furan-2-ylmethyl)-5,6-diphenyl-7H-pyrrolo[2,3-d]pyrimidin-4-amine |  |
| **62** | ethyl 2-(2-((1H-[1,2,4]triazino[5,6-b]indol-3-yl)thio)acetamido)-6-methyl-4,5,6,7-tetrahydrothieno[2,3-c]pyridine-3-carboxylate |  |
| **63** | 4-(4-phenylpiperazin-1-yl)-2-(pyridin-2-yl)quinazoline |  |
| **64** | 3-((2-(3,5-dimethyl-1H-pyrazol-1-yl)-5,6,7,8-tetrahydrobenzo[4,5]thieno[2,3-d]pyrimidin-4-yl)amino)propan-1-ol |  |
| **65** | 1-((2-(3,5-dimethyl-1H-pyrazol-1-yl)-5,6,7,8-tetrahydrobenzo[4,5]thieno[2,3-d]pyrimidin-4-yl)amino)propan-2-ol |  |
| **66** | diethyl (S)-(3-bromo-4-oxo-1-(phenylsulfonamido)-1,4-dihydronaphthalen-1-yl)phosphonate |  |
| **67** | 5-((3-(1H-imidazol-1-yl)propyl)amino)-2-(4-((4-chlorobenzyl)oxy)phenyl)oxazole-4-carbonitrile |  |
| **68** | N-(5-(4-chlorophenyl)-7-(4-methoxyphenyl)-4,5,6,7-tetrahydro-[1,2,4]triazolo[1,5-a]pyrimidin-2-yl)furan-2-carboxamide |  |
| **69** | 6-(2,6-dimethoxyphenyl)-3-(5-phenyl-1H-pyrazol-3-yl)-[1,2,4]triazolo[3,4-b][1,3,4]thiadiazole |  |
| **Ceftriaxone** | (6R,7R)-7-[[(2Z)-2-(2-amino-1,3-thiazol-4-yl)-2-methoxyiminoacetyl]amino]-3-[(2-methyl-5,6-dioxo-1H-1,2,4-triazin-3-yl)sulfanylmethyl]-8-oxo-5-thia-1-azabicyclo[4.2.0]oct-2-ene-2-carboxylic acid |  |

**Ceftriaxone:** Reference compound

**Table S2:** The SMILES name, G-score and E-model scores for best conformational pose of each ligand docked with Artemis (PDB ID: 7ABS).

| **Sr. No.** | **SMILES** | **Autodock** | **G-score** | **E-model** |
| --- | --- | --- | --- | --- |
| **1** | OC1=C(C(NC2=NC=CC=C2O)=O)C(N(CC(C)C)C3=C1CCCC3)=O | **-5.8** | **-8.5** | **-117.22** |
| **2** | OC1=C(C(NC2=NC3=CC=CC=C3N2)=O)C(N(CC)C4=CC=CC=C14)=O | **-5.6** | **-8.1** | **-86.6** |
| **3** | OC1=CC=C2C(C=CC=C2)=C1/C=N\[C@]3(CN(CC4)C5)C[N@@]5C[N@@]4C3 | -5.6 | -6.1 | -59.51 |
| **4** | O=C(C1=CC=CC=C1/C(C)=N/O)O | **-5.8** | **-8.6** | **-93.86** |
| **5** | O=C(C1=C(C2=CC=C3)C3=C(C(N4CCCCC4)=O)C=C1)N(C2=O)O | **-7.2** | **-8.4** | **-97.93** |
| **6** | O=C1N(C=CC=C2)C2=NC(N3CC4=CC=CO4)=C1C=C(C(NCC5=CC=C(C)C=C5)=O)C3=N | -7.5 | -2.5 | -44.87 |
| **7** | C12=CC=CC=C1N=C(C3=CC=CC=N3)N=C2NCC4=CC=CO4 | -6.1 | -5.2 | -58.12 |
| **8** | O=C1N(CCC2=CCCCC2)C(C)=NC3=C1C4=NC5=CC=CC=C5N=C4N3/N=C/C6=CC=CO6 | -7.3 | -3.8 | -56.65 |
| **9** | O=C(C1=CC=CS1)NC2=NN3C(NC(C(C=C4)=CC=C4OC)CC3C5=CC=CC=C5)=N2 | -6.6 | -5.4 | -55.08 |
| **10** | CC1=CC(C)=NN1C(N=C2NCCOC)=NC3=C2C(CCCC4)=C4S3 | -5.9 | -4.0 | -48.55 |
| **11** | CC1=CC=C2N(CC3=CC=CC=C3)C4=NC(SC)=NN=C4C2=C1 | -6.0 | -4.4 | -46.56 |
| **12** | N1(CCCCCC1)C2=NC(C3=CC=CC=N3)=NC4=CC=CC=C24 | -6.5 | -4.7 | -49.74 |
| **13** | ClC1=CC=C(C=C1)C2NC3=NC(NC(C4=CC=CO4)=O)=NN3C(C5=CC=CC=C5)C2 | -6.5 | -5.5 | -63.16 |
| **14** | NC1=NC(NC2=CC=CC=C2)=NC(C3=C(C)C4=CC=CC=C4O3)=N1 | -7.5 | -5.8 | -55.25 |
| **15** | O=C(CC1=C(C2=CC=CC=C2)SC=C1)NO | -5.1 | -4.5 | -46.01 |
| **16** | OC(C1=NC=CC=C1C=C2)=C2C(C3=CC=CC=N3)NC4=CC=CC=N4 | -6.3 | -4.4 | -59.27 |
| **17** | ClC1=CC=C(C(C)=C1)OCCCC(NO)=O | -4.6 | -2.3 | -38.30 |
| **18** | C1(C2=CC=CC=C2C=C3)=C3NC(C4=CC=CC=N4)=N1 | -6.2 | -4.6 | -41.60 |
| **19** | FC(F)(C1=CC=CC=C1NC2=NC(N3N=C(C)C=C3C)=NC(N4N=C(C)C=C4C)=N2)F | -6.4 | -5.3 | -63.13 |
| **20** | O=C(C1=CC=CC=C1C2=O)C(N3CCCCC3)=C2NC4=CC=CC=C4 | -6.1 | -4.1 | -46.86 |
| **21** | NC1=CC=C2NC(C3=CC=CC=N3)=NC2=C1 | -5.4 | -4.5 | -39.86 |
| **22** | ClC1=CC=CC(CON2C(C3=CC=CC=C3C2=O)=O)=C1 | -6.4 | -4.3 | -47.53 |
| **23** | N1(C(C=C2)=CC=C2C3=NC(C4=CC=CC=N4)=NC=C3)C5=CC=CC=C5N=C1 | -8.1 | -4.5 | -51.63 |
| **24** | O=C1C2=CC(N)=CC=C2C3=CC=CC=C3C1=O | -5.9 | -5.4 | -38.26 |
| **25** | COC1=CC=C(C=C1)NC2=NC(NC(C=C3)=CC=C3OC)=NC(SC4=NCCS4)=N2 | -- | -- | -- |
| **26** | ClC1=CC(Cl)=CC=C1COC2=CC=C(C=C2)N3C(C)=C(/C=N/N4C=NN=C4)C=C3C | -7.2 | -3.9 | -47.55 |
| **27** | O=C(C1=CN(C2=CC=CC=C2)N=C1C(C=C3)=CC=C3OC)NC4=NC=CS4 | -6.5 | -4.6 | -59.99 |
| **28** | O=C1C2=CC=CC=C2C(NC3=CC=C(C(OCC)=O)C=C3)=CC1=O | -7.0 | -6.8 | -73.87 |
| **29** | ClC1=CC=C(C=C1)COC2=CC=C(C=C2)N3C(C)=C(/C=N/N4C=NN=C4)C=C3C | -7.6 | -3.7 | -42.27 |
| **30** | O=C(C(C=C1)=CC=C1OC)NC2=CC=C(C=C2)N3CCCC3 | -6.1 | -4.2 | -40.86 |
| **31** | O=C(CC1=CC=CC=C1)NC2=CC=C(C=C2)N3CCCC3 | -6.6 | -3.9 | -42.40 |
| **32** | O/N=C(CCCCCCCCC)/C1=CC=CC=N1 | -5.0 | -0.7 | -31.63 |
| **33** | O=C(C1=CC=CC=C21)C(N3N=NC4=CC=CC=C34)=C(C2=O)N5CCOCC5 | -6.6 | -4.0 | -46.87 |
| **34** | O=C1C2=C(C3=CC=CC=C3C1=O)OC(C)=C2C(C4=CC=CC=C4)=O | -6.8 | -4.4 | -46.91 |
| **35** | O=C1C2=C(C3=CC=CC=C3C1=O)OC(C)=C2C(C4=CC=C(C)C=C4)=O | -7.1 | -5.1 | -54.28 |
| **36** | ClC1=CC=C(C=C1)C(C2=C(C)OC(C3=CC=CC=C3C4=O)=C2C4=O)=O | -7.2 | -4.7 | -50.36 |
| **37** | O=C1/C(SC2=CC=CC=C12)=N/N(C(C)=O)C3=CC=CC(C)=C3 | -6.8 | -3.8 | -43.71 |
| **38** | O=C1/C(SC2=CC=CC=C12)=N/N(C(C)=O)C3=CC=CC=C3C | -6.7 | -4.8 | -47.22 |
| **39** | O=C1C2=CC=CC=C2S/C1=N\N(C(C)=O)C3=CC=CC=C3 | -6.6 | -3.7 | -42.26 |
| **40** | O=C(CC(C1=CC=CC=C1)C2=CC=CC=C2)NC3=NC4=C(CN(CC)CC4)S3 | -6.4 | -3.5 | -47.39 |
| **41** | O=C(CCCSC1=CC=CC=C1)NC2=NC(C3=CC=CC=N3)=CS2 | -6.4 | -5.1 | -62.74 |
| **42** | O=S(NC1=NC=CC=N1)(C(C=C2)=CC=C2C3=CC=C(/C=N/NC(C4=CC5=CC=CC=C5C=C4O)=O)O3)=O | **-8.0** | **-8.5** | **-139.47** |
| **43** | OC(C(OC)=CC=C1)=C1/C=N/NC2=NC(NC3=CC=CC=C3)=NC(N4N=C(C)C=C4C)=N2 | **-6.2** | **-8.1** | **-114.32** |
| **44** | ClC1=CC=C(C=C1)/N=N/C2=C(N=C3C(CCCC3)=C4O)N4N=C2N | -6.2 | -8.0 | -81.99 |
| **45** | OC1=CC(N(CC)CC)=CC=C1/C=N/NC2=NC3=CC=CC=C3N2 | -6.0 | -4.2 | -45.67 |
| **46** | BrC1=CC(C(/C=C/C2=CC=CC=C2)=O)=C(C(C=C1)=O)O | -7.0 | -9.1 | -112.52 |
| **47** | O=C(C1=CC2=C(N(C3=CC=CC=C3)N=C2C)S1)NC4=NC(C)=C(C(NC5=CC=CC=C5)=O)S4 | -7.6 | -4.5 | -59.63 |
| **48** | BrC1=CC2=C(C3=CC=CC=C3)N=C(N/N=C/C4=CC=C([N+]([O-])=O)O4)N=C2C=C1 | -7.6 | -4.7 | -58.87 |
| **49** | O=C(C1=NNC=N1)NC2=CC=C3OC(C4=CC=CC5=CC=CC=C45)=NC3=C2 | -8.1 | -6.2 | -86.81 |
| **50** | OC(C(/C=N/NC1=NC(NC2=CC=CC=C2)=NC(N3N=C(C)C=C3C)=N1)=C4)=CC=C4OC | -6.6 | -5.7 | -77.89 |
| **51** | O=C(C1=CC=CC=C1N=C2N/N=C/C(C3=CC=CC=C3C=C4)=C4O)N2O | -7.6 | -9.4 | -133.21 |
| **52** | COC1=CC=C(C=C1)NC2=NC(NCC3=CC=CO3)=NC(N/N=C/C4=CC=CC=C4)=N2 | -5.3 | -5.4 | -68.15 |
| **53** | NNC1=NC(NC(C)(C)C)=NC(NC(C)(C)C)=N1 | -4.3 | -5.0 | -44.50 |
| **54** | OC(NN=C1C)=C1C(C2=C(NN=C2C)O)C3=CN(CC4=CC=CC5=CC=CC=C45)C6=CC=CC=C36 | -6.4 | -7.7 | -104.75 |
| **55** | OC1=CC(C2=CC=CC=C2)=NC(N1N=C3N)=C3/N=N/C4=CC(OC)=CC=C4 | -7.1 | -7.6 | -86.04 |
| **56** | OC1=CC(C2=CC=CC=C2)=NC(N1N=C3N)=C3/N=N/C4=CC=CC=C4 | -6.1 | -7.6 | -85.39 |
| **57** | OC1=CC(C2=CC=CC=C2)=NC(N1N=C3N)=C3/N=N/C4=CC=C(C)C=C4 | -6.5 | -7.6 | -81.72 |
| **58** | O=C(C(C(O)=C1)=CC=C1O)NNC2=C(C=CC=C3)C3=NC4=CC=CC=C24 | -6.6 | -8.8 | -101.29 |
| **59** | O=C(C1=CC=CC=C1OCC(N/N=C/C2=CC=C(C(O)=C2)O)=O)N/N=C\C3=CC=C(C(O)=C3)O | -6.8 | -7.1 | -99.68 |
| **60** | O=C1SC(SCC)=N/C1=C\C2=CC=C(C3=CC=CC=C3[N+]([O-])=O)O2 | -7.2 | -4.2 | -48.85 |
| **61** | N1(CC2=CC=CO2)C(C3=CC=CC=C3)=C(C4=CC=CC=C4)C5=C1N=CN=C5NCCCN6C=NC=C6 | -5.5 | -4.5 | -60.65 |
| **62** | O=C(C1=C(NC(CSC2=NNC3=C(C=CC=C4)C4=NC3=N2)=O)SC5=C1CCN(C)C5)OCC | -5.5 | -5.6 | -71.65 |
| **63** | N1(CCN(C2=CC=CC=C2)CC1)C3=NC(C4=CC=CC=N4)=NC5=CC=CC=C35 | -7.7 | -4.8 | -56.83 |
| **64** | OCCCNC1=C2C(SC3=C2CCCC3)=NC(N4N=C(C)C=C4C)=N1 | -5.5 | -4.7 | -51.66 |
| **65** | OC(C)CNC1=NC(N2N=C(C)C=C2C)=NC3=C1C(CCCC4)=C4S3 | -6.4 | -5.2 | -57.12 |
| **66** | BrC1=C[C@@](NS(=O)(C2=CC=CC=C2)=O)(C3=CC=CC=C3C1=O)P(OCC)(OCC)=O | -5.2 | -7.4 | -99.17 |
| **67** | ClC1=CC=C(C=C1)COC2=CC=C(C=C2)C3=NC(C#N)=C(NCCCN4C=NC=C4)O3 | -6.4 | -6.5 | -93.25 |
| **68** | ClC1=CC=C(C=C1)C2NC3=NC(NC(C4=CC=CO4)=O)=NN3C(C(C=C5)=CC=C5OC)C2 | -7.2 | -4.8 | -58.31 |
| **69** | COC1=CC=CC(OC)=C1C2=NN3C(S2)=NN=C3C4=NNC(C5=CC=CC=C5)=C4 | -7.5 | -4.7 | -59.12 |
| **Ceftriaxone** | O=C(C(N12)=C(CSC(N(C)NC3=O)=NC3=O)CS[C@]2([H])[C@H](NC(/C(C4=CSC(N)=N4)=N\OC)=O)C1=O)O | -6.6 | -9.9 | -165.02 |

**Reference:** Ceftriaxone

**Table S3:** The ADMET properties including physicochemical parameters, absorption, distribution and bioavailability of investigated compounds.

| **Sr. no.** | **MW** | **TPSA** | **logP** | **P-gp_inh** | **P-gp_sub** | **HIA** | **F20** | **Caco2** | **PPB** |
| --- | --- | --- | --- | --- | --- | --- | --- | --- | --- |
| **1** | 357.17 | 104.45 | 2.27 | 0.39 | 0.00 | 0.02 | 0.11 | -5.10 | 97.93 |
| **2** | 348.12 | 100.01 | 2.74 | 0.09 | 0.02 | 0.01 | 0.01 | -5.16 | 98.10 |
| **3** | 322.18 | 42.31 | 1.94 | 0.00 | 0.99 | 0.00 | 0.05 | -5.16 | 83.73 |
| **4** | 179.06 | 69.89 | 0.72 | 0.00 | 0.04 | 0.02 | 0.06 | -5.42 | 71.21 |
| **5** | 324.11 | 77.92 | 1.12 | 0.69 | 0.11 | 0.72 | 0.09 | -4.56 | 87.62 |
| **6** | 439.16 | 105.39 | 2.80 | 0.98 | 0.00 | 0.01 | 0.01 | -5.13 | 98.23 |
| **7** | 302.12 | 63.84 | 3.05 | 0.71 | 0.00 | 0.00 | 0.02 | -5.11 | 97.61 |
| **8** | 452.2 | 91.1 | 4.24 | 0.91 | 0.01 | 0.00 | 0.12 | -5.00 | 96.97 |
| **9** | 431.14 | 81.07 | 3.59 | 0.59 | 0.33 | 0.00 | 0.00 | -4.92 | 97.02 |
| **10** | 357.16 | 64.86 | 4.27 | 0.92 | 0.00 | 0.00 | 0.01 | -4.85 | 97.24 |
| **11** | 320.11 | 43.6 | 4.75 | 0.98 | 0.00 | 0.01 | 0.04 | -5.15 | 98.66 |
| **12** | 304.17 | 41.91 | 3.54 | 0.85 | 0.01 | 0.00 | 0.00 | -4.39 | 98.11 |
| **13** | 419.11 | 84.98 | 3.77 | 0.06 | 0.37 | 0.00 | 0.00 | -4.71 | 98.03 |
| **14** | 317.13 | 89.86 | 3.54 | 0.71 | 0.02 | 0.02 | 0.01 | -4.77 | 98.19 |
| **15** | 233.05 | 49.33 | 2.18 | 0.00 | 0.19 | 0.00 | 0.05 | -4.99 | 89.55 |
| **16** | 328.13 | 70.93 | 2.43 | 9.05 | 0.00 | 0.00 | 0.03 | -4.94 | 93.93 |
| **17** | 243.07 | 58.56 | 2.35 | 0.34 | 0.52 | 0.00 | 0.00 | -4.64 | 94.54 |
| **18** | 245.1 | 41.57 | 3.38 | 0.64 | 0.10 | 0.00 | 0.01 | -4.71 | 97.90 |
| **19** | 428.17 | 86.34 | 4.46 | 0.99 | 0.01 | 0.00 | 0.00 | -4.95 | 98.51 |
| **20** | 332.15 | 49.41 | 3.50 | 0.99 | 0.00 | 0.63 | 0.00 | -4.54 | 98.26 |
| **21** | 210.09 | 67.59 | 1.71 | 0.05 | 0.28 | 0.00 | 0.01 | -4.80 | 73.59 |
| **22** | 287.03 | 46.61 | 2.60 | 0.49 | 0.01 | 0.01 | 0.01 | -4.33 | 98.44 |
| **23** | 349.13 | 56.49 | 3.41 | 0.61 | 0.00 | 0.00 | 0.02 | -4.13 | 97.48 |
| **24** | 223.06 | 60.16 | 2.32 | 0.97 | 0.00 | 0.97 | 0.01 | -4.51 | 94.53 |
| **26** | 439.1 | 57.23 | 4.46 | 0.00 | 0.00 | 0.00 | 0.00 | -4.95 | 98.20 |
| **27** | 376.1 | 69.04 | 3.84 | 0.90 | 0.00 | 0.05 | 0.12 | -4.84 | 98.81 |
| **28** | 321.1 | 72.47 | 3.22 | 0.94 | 0.00 | 0.34 | 0.04 | -4.97 | 97.59 |
| **29** | 405.14 | 57.23 | 3.93 | 0.00 | 0.00 | 0.00 | 0.00 | -4.95 | 97.67 |
| **30** | 296.15 | 41.57 | 3.62 | 0.61 | 0.70 | 0.02 | 0.03 | -5.02 | 96.53 |
| **31** | 280.16 | 32.34 | 3.53 | 0.24 | 0.08 | 0.00 | 0.01 | -4.72 | 97.14 |
| **32** | 248.19 | 45.48 | 4.52 | 0.03 | 0.00 | 0.09 | 0.51 | -5.08 | 99.31 |
| **33** | 360.12 | 77.32 | 1.50 | 0.98 | 0.00 | 0.23 | 0.00 | -4.34 | 94.57 |
| **34** | 316.07 | 64.35 | 3.20 | 0.99 | 0.00 | 0.05 | 0.00 | -4.71 | 97.55 |
| **35** | 330.09 | 64.35 | 3.80 | 0.99 | 0.00 | 0.02 | 0.01 | -4.71 | 98.68 |
| **36** | 350.03 | 64.35 | 3.79 | 0.99 | 0.00 | 0.01 | 0.00 | -4.64 | 99.01 |
| **37** | 310.08 | 49.74 | 2.93 | 0.74 | 0.00 | 0.00 | 0.00 | -4.51 | 98.68 |
| **38** | 310.08 | 49.74 | 2.89 | 0.85 | 0.00 | 0.00 | 0.00 | -4.41 | 98.69 |
| **39** | 296.06 | 49.74 | 2.60 | 0.78 | 0.00 | 0.00 | 0.00 | -4.34 | 98.22 |
| **40** | 391.17 | 45.23 | 3.86 | 0.99 | 0.01 | 0.00 | 0.01 | -5.23 | 99.01 |
| **41** | 355.08 | 54.88 | 4.53 | 0.55 | 0.00 | 0.01 | 0.01 | -4.75 | 97.85 |
| **42** | 513.11 | 146.78 | 3.74 | 0.00 | 0.04 | 0.00 | 0.92 | -5.85 | 98.81 |
| **43** | 430.19 | 122.37 | 4.84 | 0.05 | 0.06 | 0.00 | 0.64 | -5.14 | 98.87 |
| **44** | 342.1 | 101.16 | 3.60 | 0.03 | 0.00 | 0.00 | 0.03 | -4.86 | 98.62 |
| **45** | 323.17 | 76.54 | 4.13 | 0.00 | 0.90 | 0.00 | 0.94 | -5.40 | 96.93 |
| **46** | 329.99 | 54.37 | 3.29 | 0.99 | 0.00 | 0.00 | 0.04 | -4.89 | 98.88 |
| **47** | 473.1 | 88.91 | 4.55 | 0.89 | 0.00 | 0.00 | 0.01 | -4.79 | 98.29 |
| **48** | 437.01 | 106.45 | 4.55 | 0.02 | 0.00 | 0.00 | 0.02 | -4.97 | 99.00 |
| **49** | 355.11 | 96.7 | 3.97 | 0.00 | 0.02 | 0.00 | 0.01 | -4.97 | 98.67 |
| **50** | 430.19 | 122.37 | 5.25 | 0.08 | 0.02 | 0.00 | 0.64 | -5.16 | 98.20 |
| **51** | 346.11 | 99.74 | 3.33 | 0.00 | 0.19 | 0.00 | 0.81 | -4.94 | 98.86 |
| **52** | 415.18 | 109.49 | 4.69 | 0.04 | 0.00 | 0.00 | 0.92 | -5.11 | 98.57 |
| **53** | 253.2 | 100.78 | 2.75 | 0.98 | 0.04 | 0.01 | 0.21 | -4.66 | 88.27 |
| **54** | 463.2 | 102.75 | 4.10 | 0.00 | 0.31 | 0.00 | 0.05 | -5.25 | 98.06 |
| **55** | 360.13 | 110.39 | 3.07 | 0.05 | 0.00 | 0.00 | 0.04 | -4.85 | 98.20 |
| **56** | 330.12 | 101.16 | 3.13 | 0.08 | 0.00 | 0.00 | 0.05 | -4.96 | 97.67 |
| **57** | 344.14 | 101.16 | 3.39 | 0.15 | 0.00 | 0.00 | 0.26 | -4.94 | 97.76 |
| **58** | 345.11 | 94.48 | 2.78 | 0.02 | 0.04 | 0.00 | 0.04 | -4.99 | 97.95 |
| **59** | 462.15 | 152.84 | 2.59 | 0.00 | 0.51 | 0.00 | 0.99 | -5.46 | 97.73 |
| **60** | 360.02 | 85.71 | 3.49 | 0.99 | 0.00 | 0.33 | 0.00 | -4.72 | 98.80 |
| **61** | 474.22 | 73.7 | 4.09 | 0.95 | 0.21 | 0.00 | 0.00 | -5.20 | 97.43 |
| **62** | 482.12 | 113.1 | 3.37 | 0.90 | 0.00 | 0.01 | 0.13 | -5.17 | 96.75 |
| **63** | 367.18 | 45.15 | 3.15 | 0.87 | 0.94 | 0.00 | 0.00 | -4.89 | 98.20 |
| **64** | 357.16 | 75.86 | 4.04 | 0.52 | 0.04 | 0.00 | 0.00 | -5.01 | 97.23 |
| **65** | 357.16 | 75.86 | 3.69 | 0.79 | 0.00 | 0.00 | 0.00 | -5.03 | 98.45 |
| **66** | 513 | 98.77 | 2.20 | 0.99 | 0.00 | 0.00 | 0.00 | -4.49 | 98.12 |
| **67** | 433.13 | 88.9 | 4.73 | 0.56 | 0.00 | 0.00 | 0.11 | -5.03 | 98.23 |
| **68** | 449.13 | 94.21 | 3.80 | 0.09 | 0.26 | 0.00 | 0.00 | -4.82 | 97.82 |
| **69** | 404.11 | 90.22 | 3.61 | 0.38 | 0.00 | 0.01 | 0.06 | -5.19 | 97.55 |
| **ceftriaxone** | 554.05 | 214.96 | -0.60 | 0.01 | 0.00 | 0.99 | 0.97 | -6.58 | 92.00 |

**MW:** Molecular Weight **TPSA:** Topological polar surface area **P-gp:** p-glycoprotein HIA: Human intestinal absorption **F20%:** Bioavailability **Caco-2:** Caco-2 cell membrane permeability **PPB:** Plasma Protein Binding

**Table S4:** The ADMET properties including Blood brain penetration, metabolism and excretion in terms of half-life of investigated ligands.

| **Sr. no.** | **BBB** | **CYP2D6-inh** | **CYP2D6-sub** | **CYP3A4-inh** | **CYP3A4-sub** | **T1/2** |
| --- | --- | --- | --- | --- | --- | --- |
| **1** | 0.00 | 0.00 | 0.00 | 0.00 | 0.99 | 0.69 |
| **2** | 0.66 | 0.00 | 0.00 | 0.00 | 0.72 | 1.10 |
| **3** | 0.91 | 0.00 | 0.00 | 0.00 | 0.99 | 0.98 |
| **4** | 0.00 | 0.00 | 0.00 | 0.00 | 0.26 | 1.53 |
| **5** | 0.10 | 0.00 | 0.96 | 0.51 | 0.01 | 1.20 |
| **6** | 0.06 | 0.02 | 0.03 | 0.99 | 0.01 | 0.55 |
| **7** | 0.97 | 0.73 | 0.06 | 0.99 | 0.01 | 0.59 |
| **8** | 0.01 | 0.02 | 0.25 | 0.21 | 0.02 | 0.56 |
| **9** | 0.00 | 0.00 | 0.01 | 0.99 | 0.57 | 0.85 |
| **10** | 0.54 | 0.00 | 0.89 | 0.99 | 0.76 | 0.26 |
| **11** | 0.57 | 0.01 | 0.11 | 0.15 | 0.99 | 0.52 |
| **12** | 0.99 | 0.86 | 0.90 | 0.70 | 0.89 | 0.25 |
| **13** | 0.89 | 0.00 | 0.04 | 0.88 | 0.01 | 0.60 |
| **14** | 0.17 | 0.06 | 0.02 | 0.02 | 0.01 | 0.83 |
| **15** | 0.80 | 0.00 | 0.00 | 0.29 | 0.00 | 1.28 |
| **16** | 0.01 | 0.00 | 0.99 | 0.00 | 0.14 | 0.69 |
| **17** | 0.39 | 0.00 | 0.00 | 0.09 | 0.01 | 1.00 |
| **18** | 0.93 | 0.01 | 0.77 | 0.31 | 0.07 | 0.86 |
| **19** | 0.19 | 0.00 | 0.00 | 0.99 | 0.17 | 0.28 |
| **20** | 0.00 | 0.87 | 0.00 | 0.01 | 0.01 | 0.30 |
| **21** | 0.69 | 0.00 | 0.08 | 0.36 | 0.02 | 1.19 |
| **22** | 0.98 | 0.02 | 0.03 | 0.90 | 0.05 | 0.90 |
| **23** | 0.81 | 0.06 | 0.99 | 0.98 | 0.37 | 0.69 |
| **24** | 0.00 | 0.98 | 0.00 | 0.57 | 0.01 | 1.66 |
| **26** | 0.00 | 0.00 | 0.95 | 0.99 | 0.99 | 0.69 |
| **27** | 0.10 | 0.00 | 0.99 | 0.38 | 0.00 | 0.69 |
| **28** | 0.00 | 0.99 | 0.00 | 0.95 | 0.00 | 0.52 |
| **29** | 0.01 | 0.00 | 0.96 | 0.98 | 0.99 | 0.52 |
| **30** | 0.06 | 0.018 | 0.99 | 0.13 | 0.00 | 0.86 |
| **31** | 0.82 | 0.047 | 0.01 | 0.21 | 0.03 | 0.76 |
| **32** | 0.01 | 0.053 | 0.03 | 0.00 | 0.02 | 0.50 |
| **33** | 0.00 | 0.00 | 0.00 | 0.54 | 0.03 | 0.76 |
| **34** | 0.04 | 0.97 | 0.00 | 0.89 | 0.00 | 0.85 |
| **35** | 0.02 | 0.95 | 0.00 | 0.97 | 0.01 | 0.67 |
| **36** | 0.13 | 0.99 | 0.00 | 0.87 | 0.07 | 0.83 |
| **37** | 0.19 | 0.00 | 0.00 | 0.00 | 0.00 | 0.53 |
| **38** | 0.32 | 0.00 | 0.00 | 0.02 | 0.05 | 0.52 |
| **39** | 0.17 | 0.00 | 0.00 | 0.00 | 0.00 | 0.62 |
| **40** | 0.98 | 0.99 | 0.16 | 0.99 | 0.95 | 0.76 |
| **41** | 0.88 | 0.93 | 0.23 | 0.99 | 0.24 | 0.20 |
| **42** | 0.00 | 0.00 | 0.00 | 0.00 | 0.99 | 0.63 |
| **43** | 0.00 | 0.00 | 0.02 | 0.99 | 0.01 | 0.54 |
| **44** | 0.00 | 0.00 | 0.24 | 0.00 | 0.00 | 0.71 |
| **45** | 0.00 | 0.99 | 0.00 | 0.99 | 0.00 | 0.37 |
| **46** | 0.83 | 0.01 | 0.01 | 0.00 | 0.00 | 0.98 |
| **47** | 0.03 | 0.00 | 0.00 | 0.92 | 0.03 | 0.71 |
| **48** | 0.00 | 1.00 | 0.98 | 0.89 | 0.11 | 0.82 |
| **49** | 0.78 | 0.00 | 0.00 | 0.02 | 0.06 | 0.86 |
| **50** | 0.00 | 0.00 | 0.06 | 0.99 | 0.00 | 0.43 |
| **51** | 0.00 | 0.01 | 0.01 | 0.89 | 0.01 | 0.78 |
| **52** | 0.00 | 0.00 | 0.01 | 0.99 | 0.00 | 0.43 |
| **53** | 0.00 | 0.00 | 0.98 | 0.01 | 0.96 | 1.19 |
| **54** | 0.15 | 0.00 | 0.01 | 0.01 | 0.99 | 0.89 |
| **55** | 0.00 | 0.10 | 0.00 | 0.98 | 0.00 | 0.90 |
| **56** | 0.00 | 0.00 | 0.00 | 0.93 | 0.00 | 0.94 |
| **57** | 0.00 | 0.00 | 0.02 | 0.16 | 0.00 | 0.94 |
| **58** | 0.097 | 0.99 | 0.98 | 0.99 | 0.01 | 1.25 |
| **59** | 0.00 | 0.00 | 0.00 | 0.01 | 0.00 | 1.08 |
| **60** | 0.914 | 0.72 | 0.04 | 0.97 | 0.82 | 0.66 |
| **61** | 0.95 | 1.00 | 0.00 | 1.00 | 0.03 | 0.39 |
| **62** | 0.26 | 0.79 | 0.99 | 0.85 | 0.85 | 0.85 |
| **63** | 0.97 | 0.00 | 0.03 | 0.96 | 0.81 | 0.27 |
| **64** | 0.46 | 0.00 | 0.71 | 0.23 | 0.03 | 0.34 |
| **65** | 0.07 | 0.00 | 0.88 | 0.00 | 0.11 | 0.50 |
| **66** | 0.00 | 0.00 | 0.11 | 0.99 | 0.02 | 0.87 |
| **67** | 0.00 | 1.00 | 0.74 | 1.00 | 0.09 | 0.73 |
| **68** | 0.10 | 0.00 | 0.76 | 0.98 | 0.00 | 0.56 |
| **69** | 0.01 | 0.00 | 0.98 | 0.99 | 0.00 | 0.50 |
| **ceftriaxone** | 0.01 | 0.01 | 0.00 | 0.00 | 0.00 | 1.92 |

**BBB:** Blood Brain Barrier (enhanced penetration with values close to 1). **T1/2:** Half Life

**Table S5:** MMGBSA ΔG binding energy calculations for compounds 42, 51, and ceftriaxone in complex with Artemis protein (PDB ID: 7ABS).

| **Ligand-Protein complex** | **0 ns** | **50 ns** | **100 ns** | **Average** |
| --- | --- | --- | --- | --- |
| **Compound 42-Artemis complex** | -37.48 | -37.23 | -38.56 | -36.94 |
| **Compound 51-Artemis complex** | -15.58 | -05.58 | -02.67 | -06.23 |
| **Compound Cef-Artemis complex** | -18.93 | -30.12 | -27.71 | -24.44 |


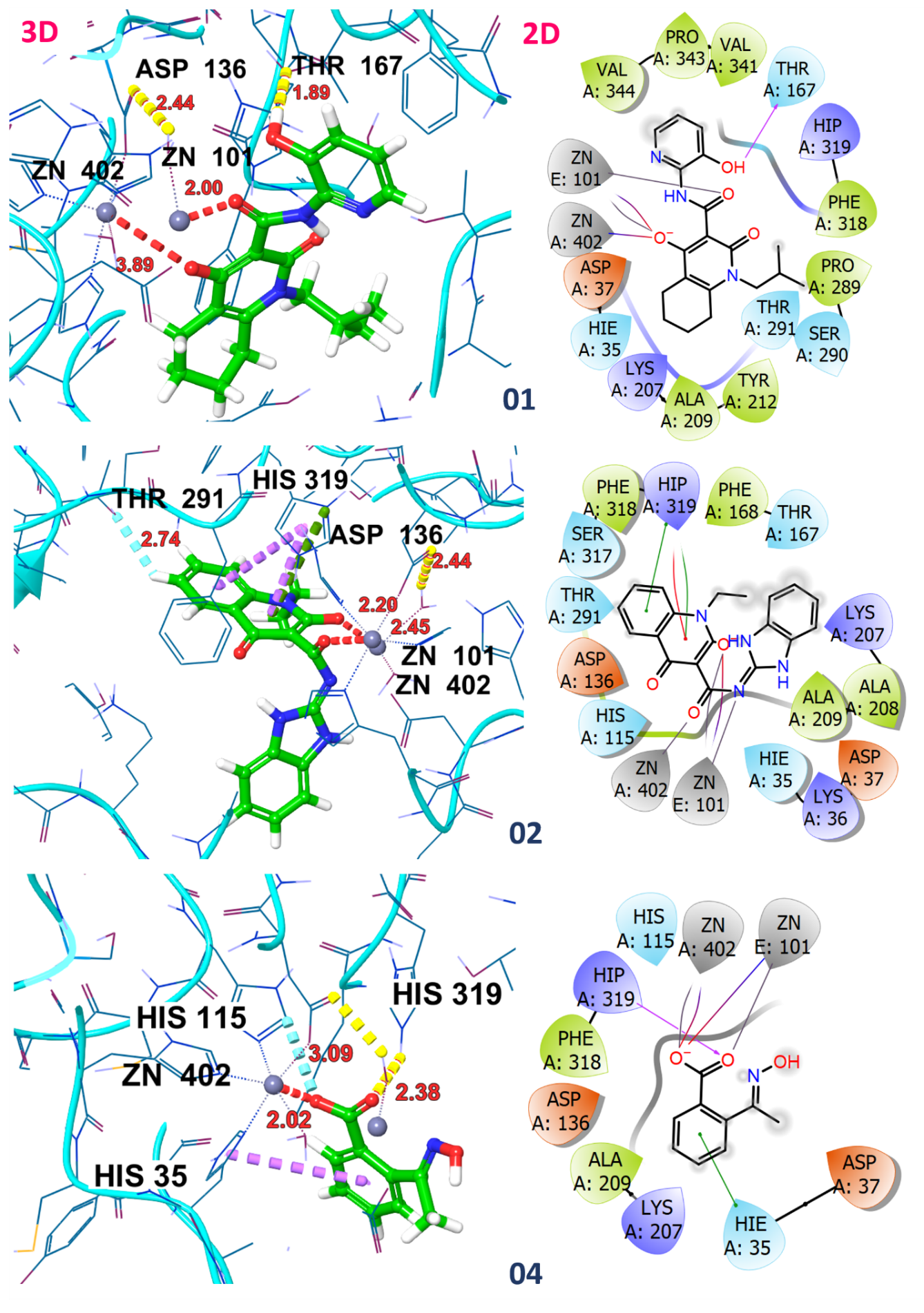


**Figure S1:** 2D and 3D interaction of investigated ligands 01, 02 and 04 with Artemis protein (PDB ID: 7ABS).

**
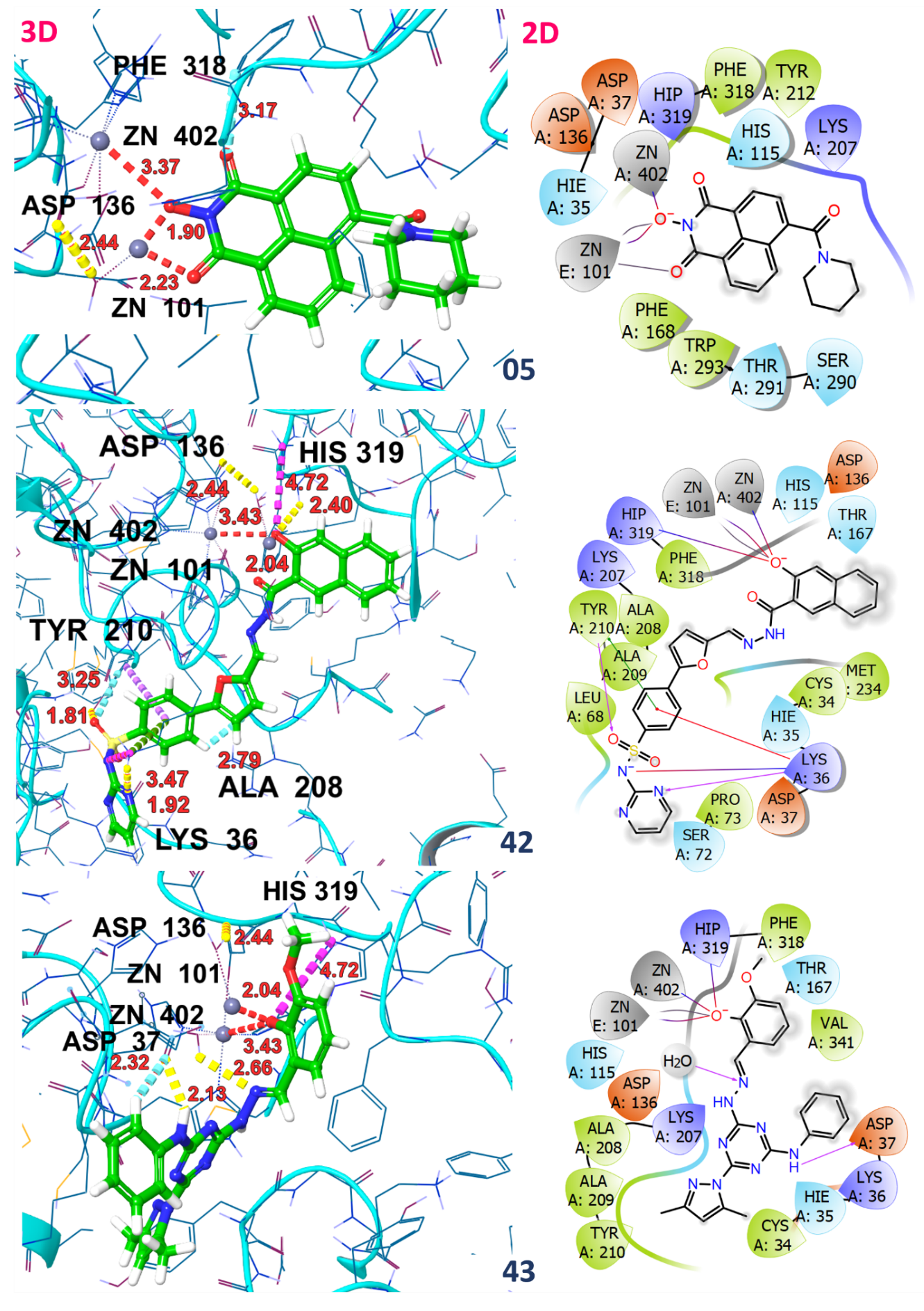
**

**Figure S2:** 2D and 3D interaction of investigated ligands 05, 42 and 43 with Artemis protein (PDB ID: 7ABS).

**
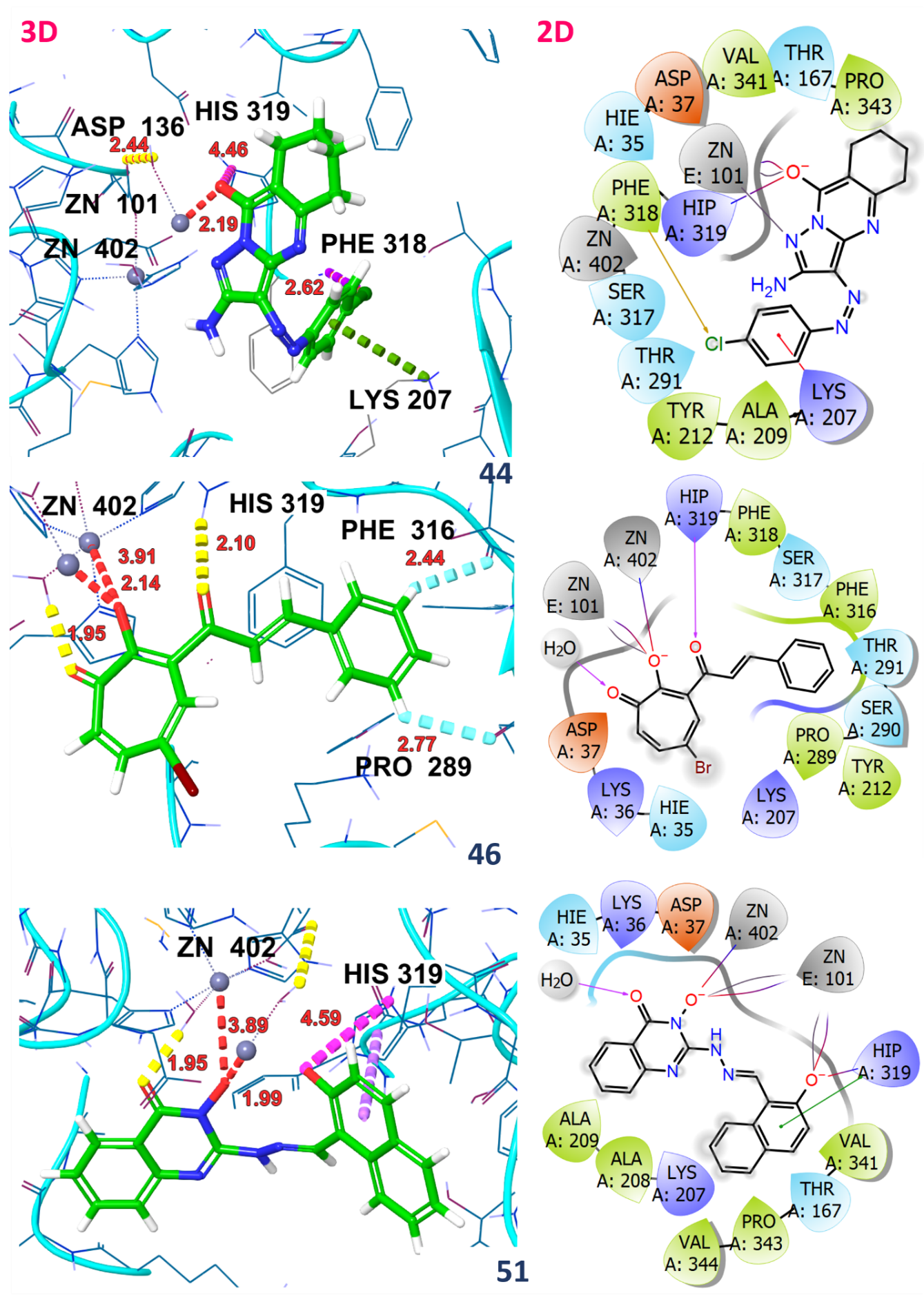
**

**Figure S3:** 2D and 3D interaction of investigated ligands 44, 46 and 51 with Artemis protein (PDB ID: 7ABS).

**
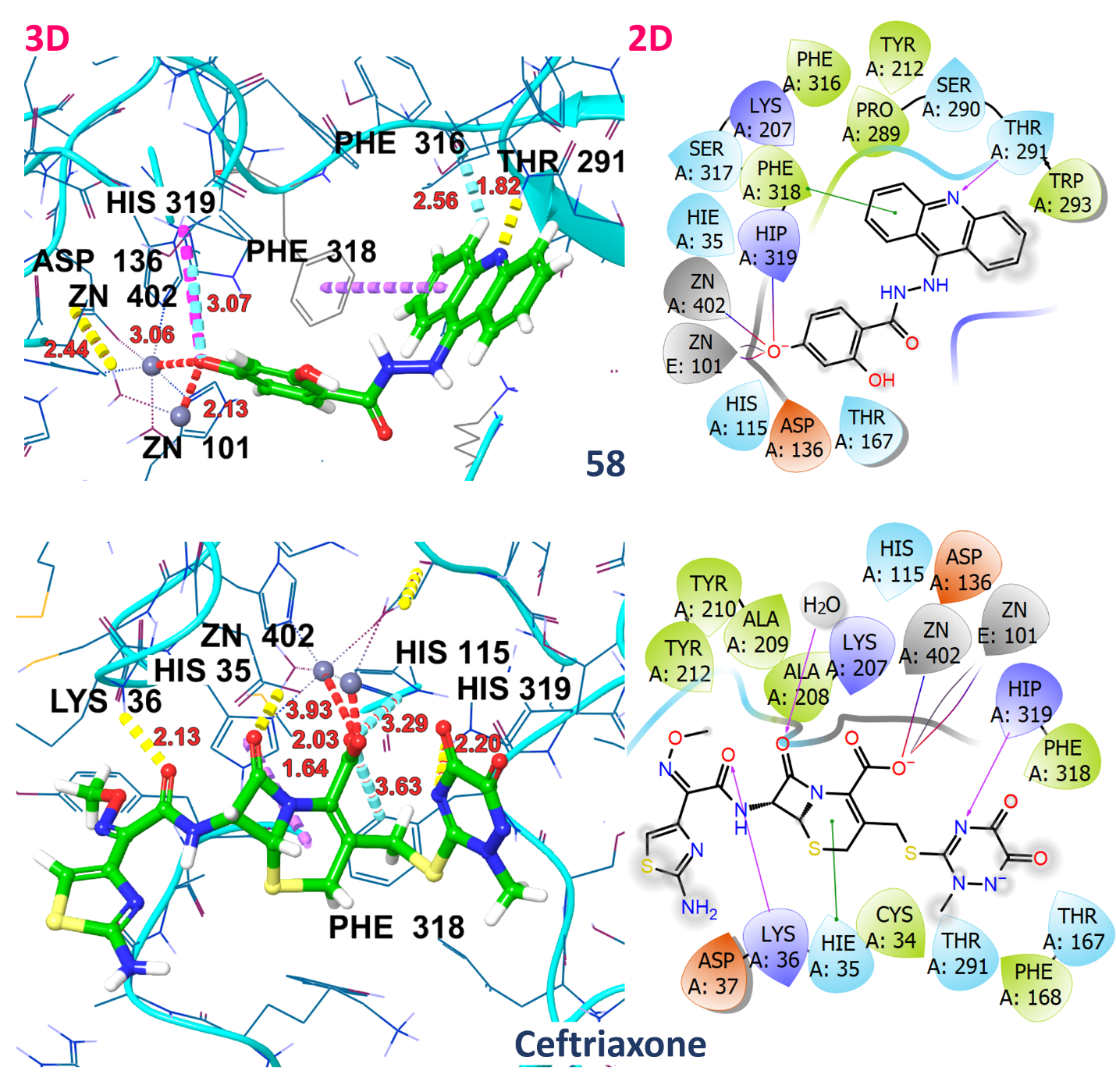
**

**Figure S4:** 2D and 3D interaction of investigated ligands 58 and ceftriaxone with Artemis protein (PDB ID: 7ABS).

**
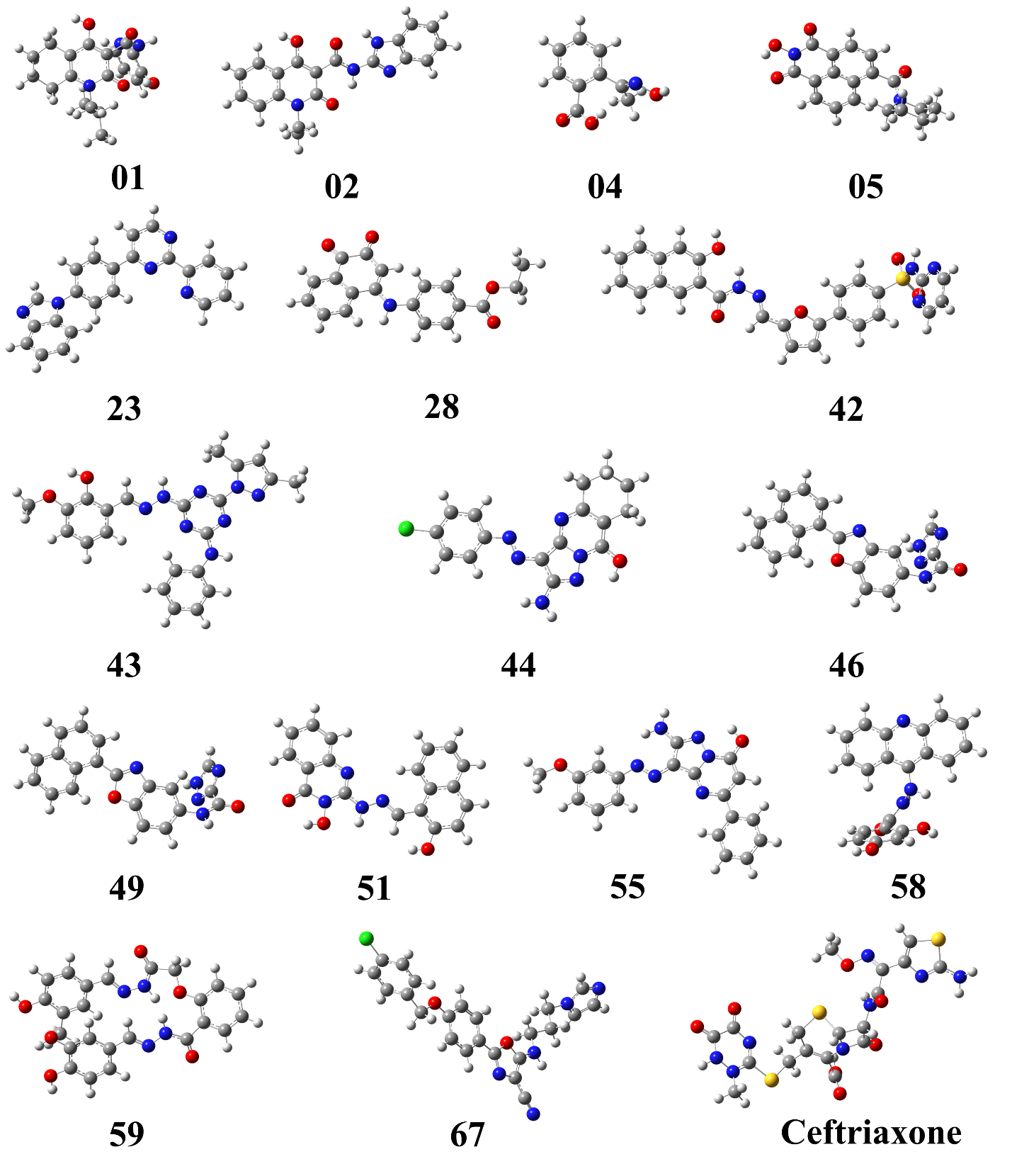
**

**Figure S5:** Optimized structures of the investigated compounds at B3LYP/6-311+g(d, p) level of DFT calculations in the gas phase.


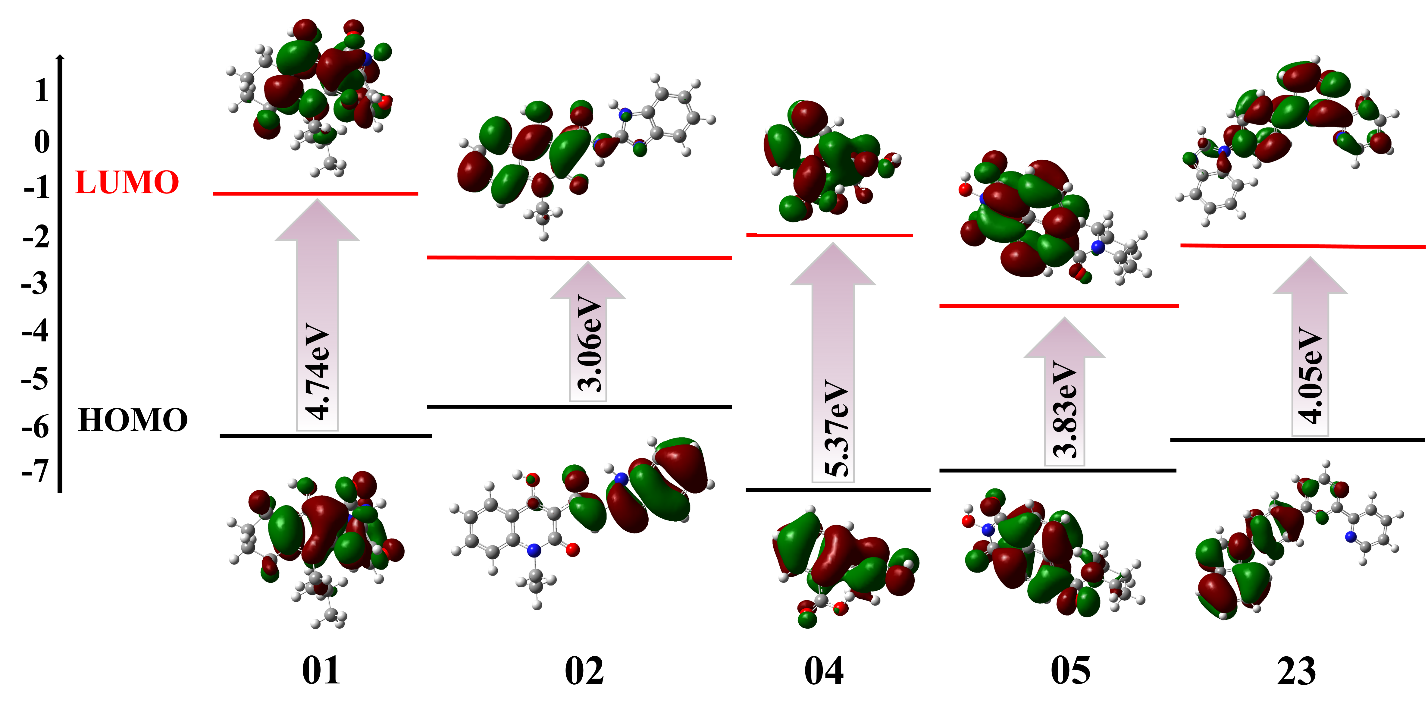


**Figure S6:** The contour plots of HOMOs and LUMOs for compounds 01, 02, 04, 05 and 23.


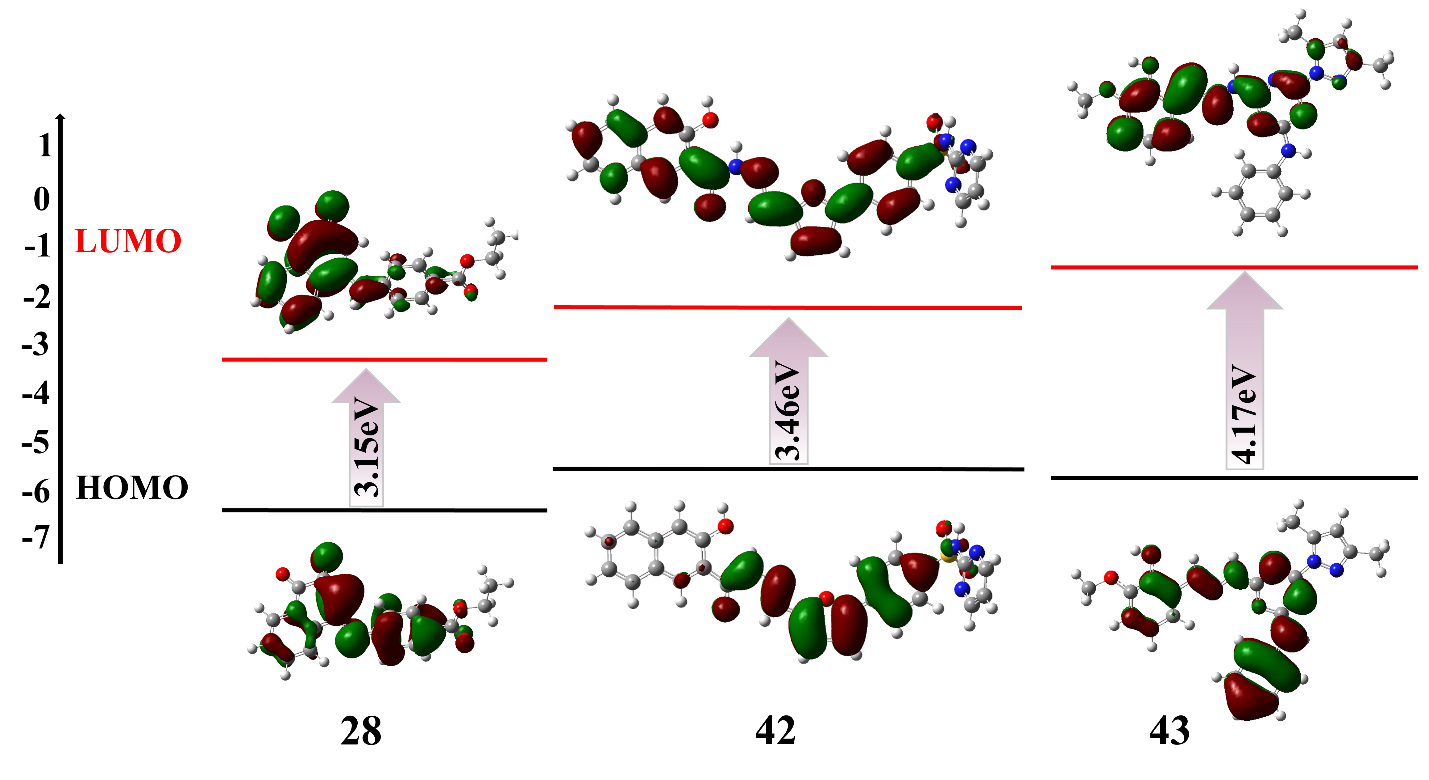


**Figure S7:** The contour plots of HOMOs and LUMOs for compounds 28, 42 and 43.


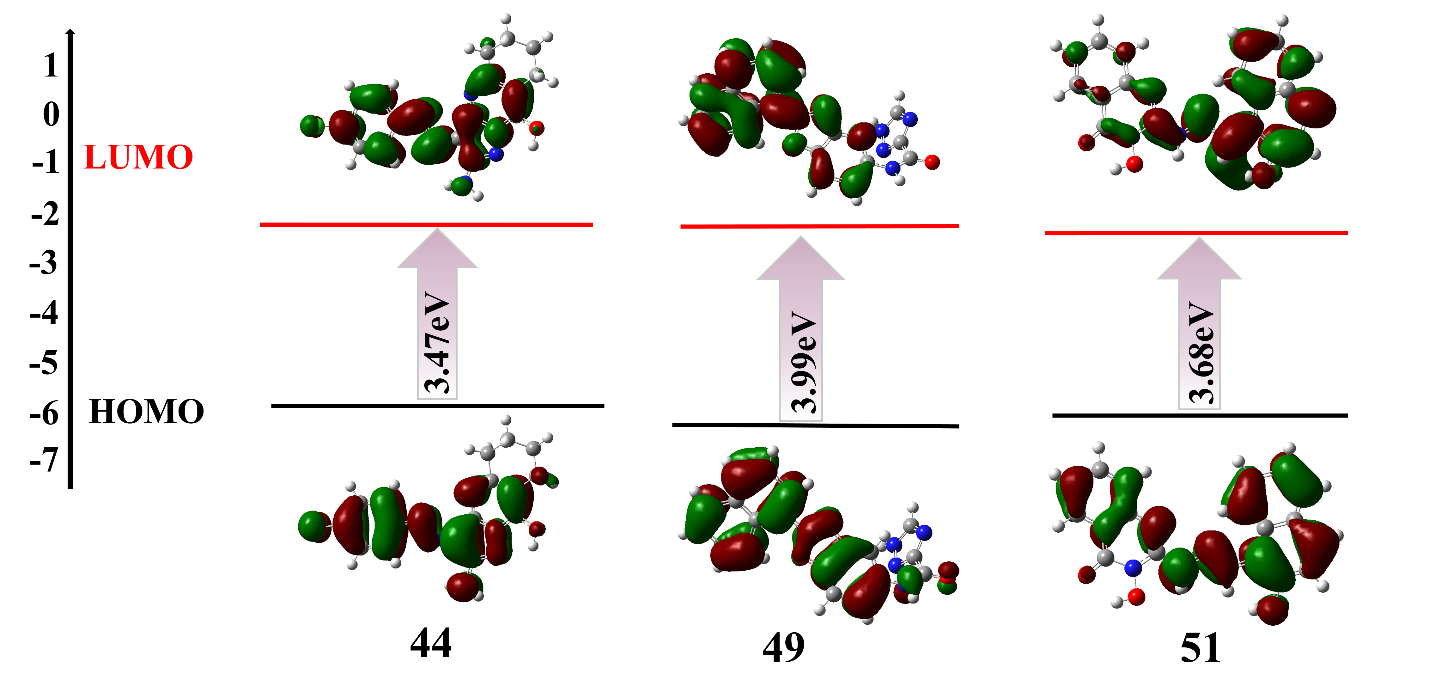


**Figure S8:** The contour plots of HOMOs and LUMOs for compounds 44, 46, 49 and 51.


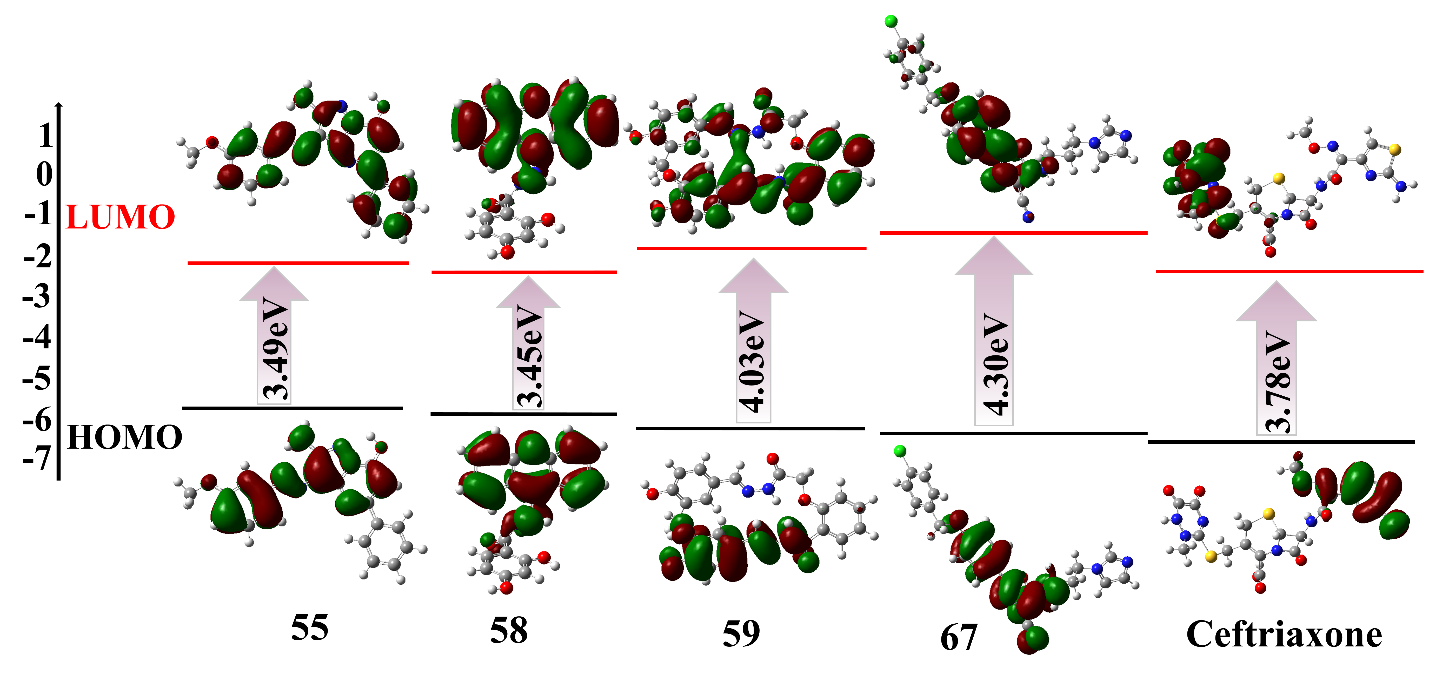


**Figure S9:** The contour plots of HOMOs and LUMOs for compounds 55, 58, 59, 67 and ceftriaxone.

#
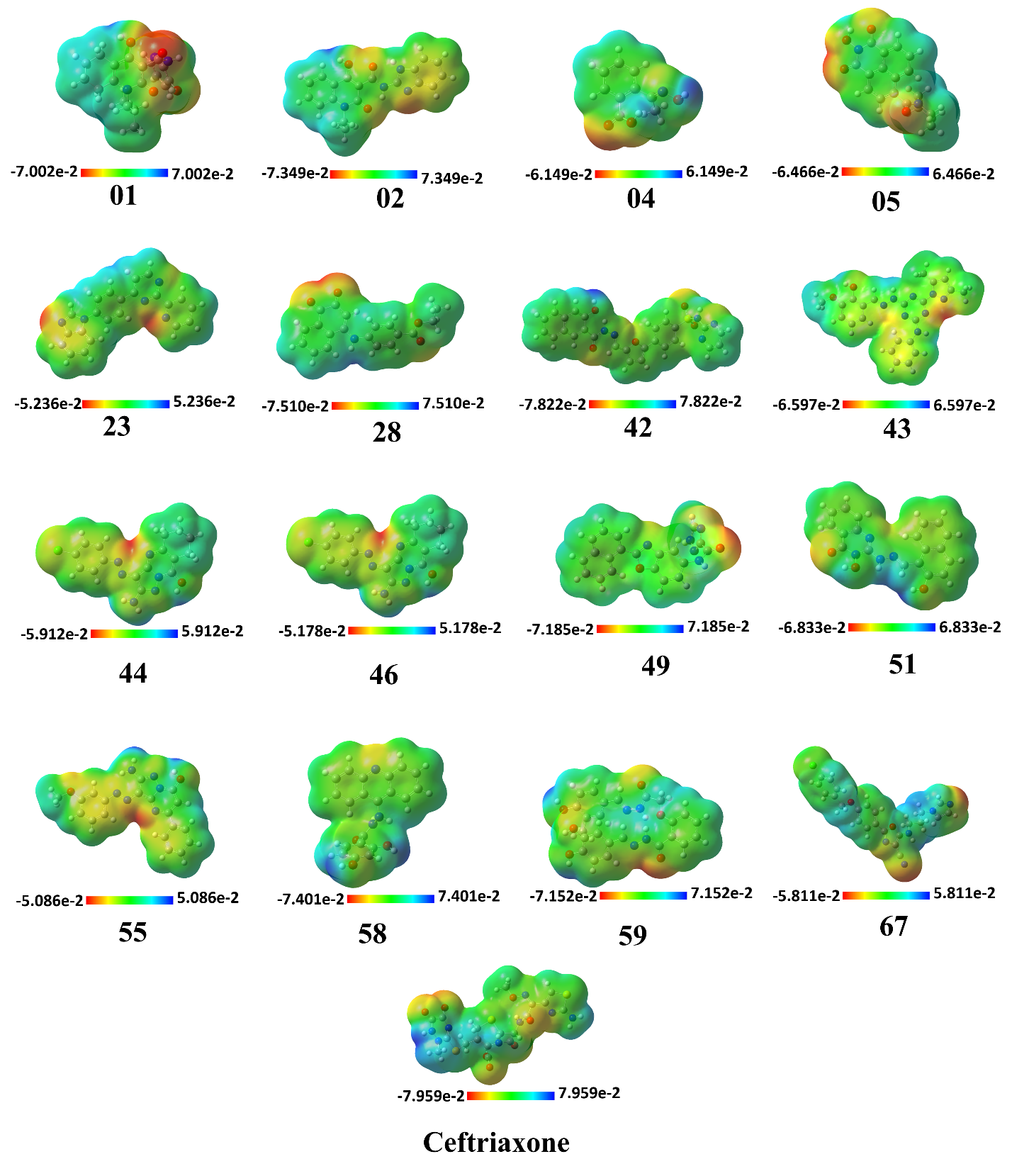
Figure S10: Molecular Electrostatic Potential (MEP) maps for the investigated compounds.


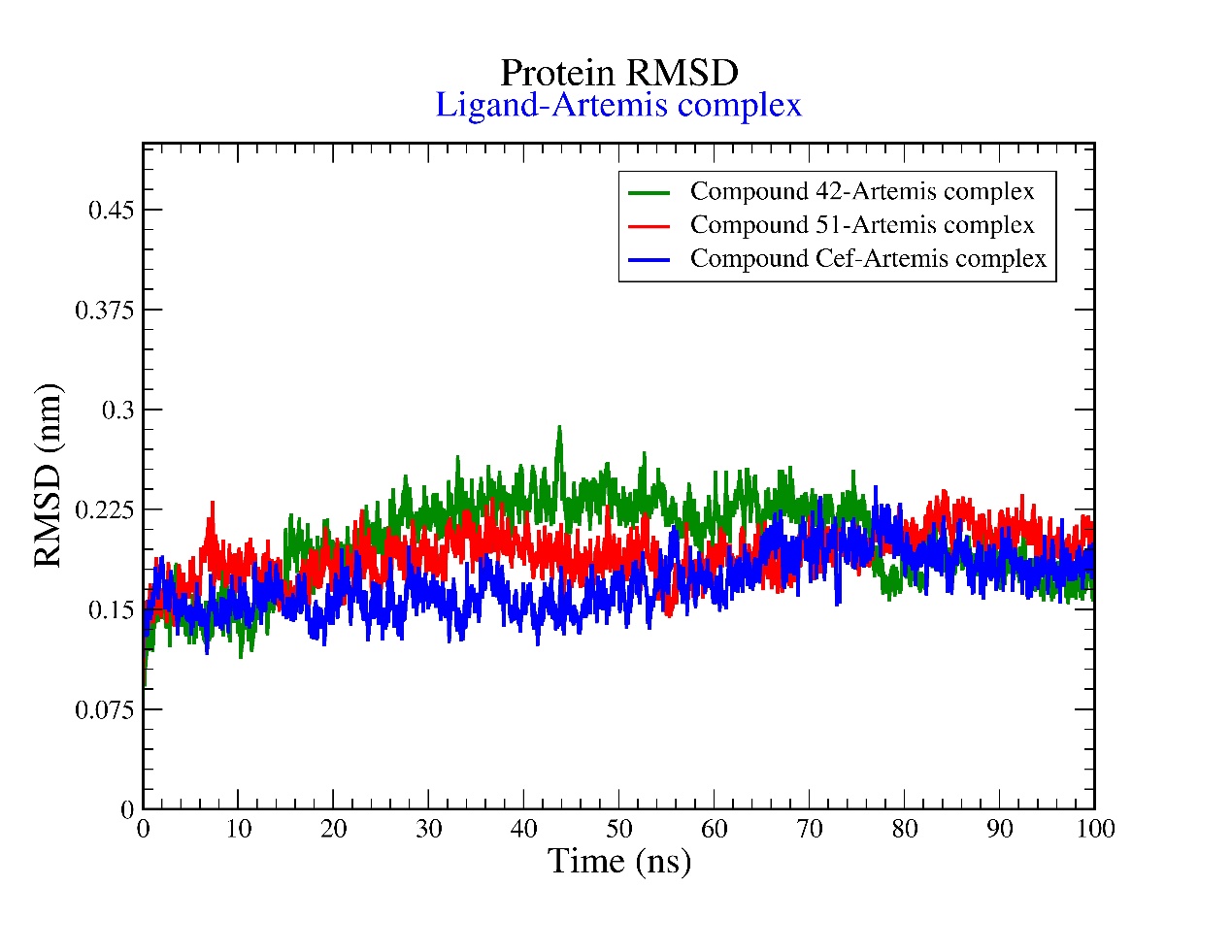


**Figure S11:** Graphical plots of Artemis-protein RMSD (nm) versus time (100 ns) for compounds 42, 51, and ceftriaxone in complex with the target protein.


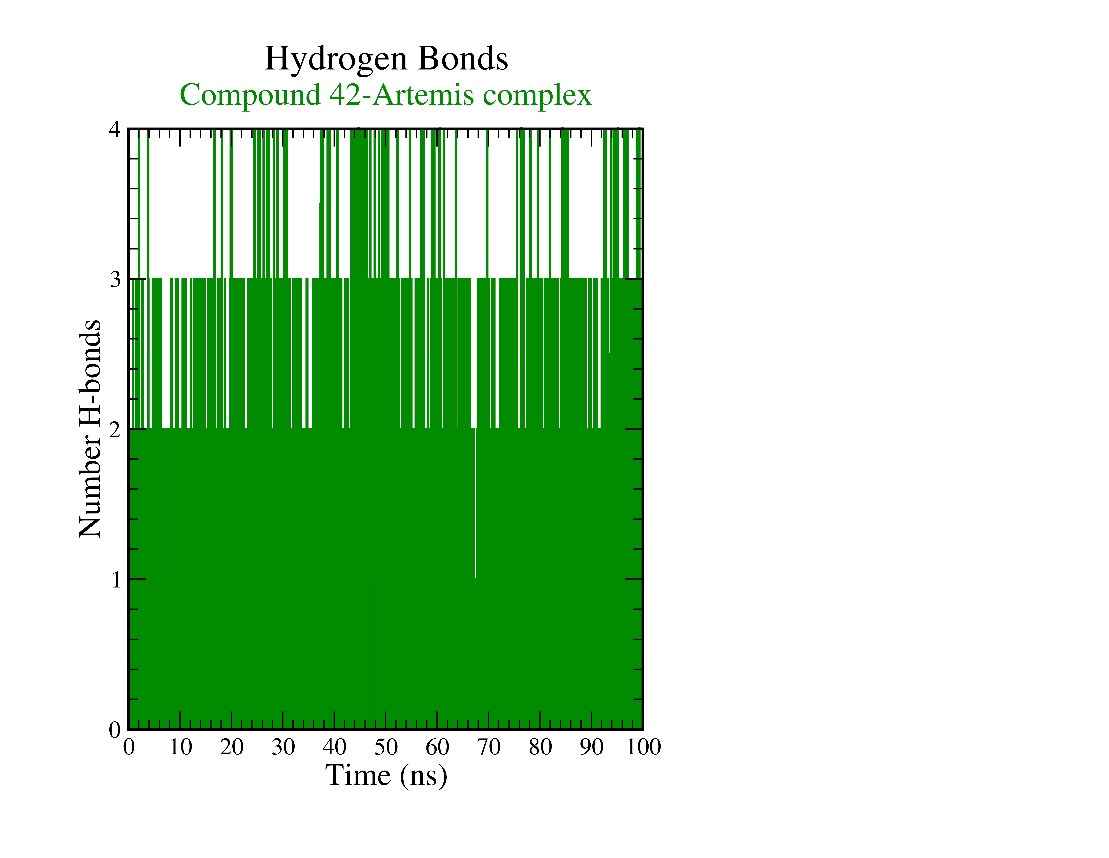

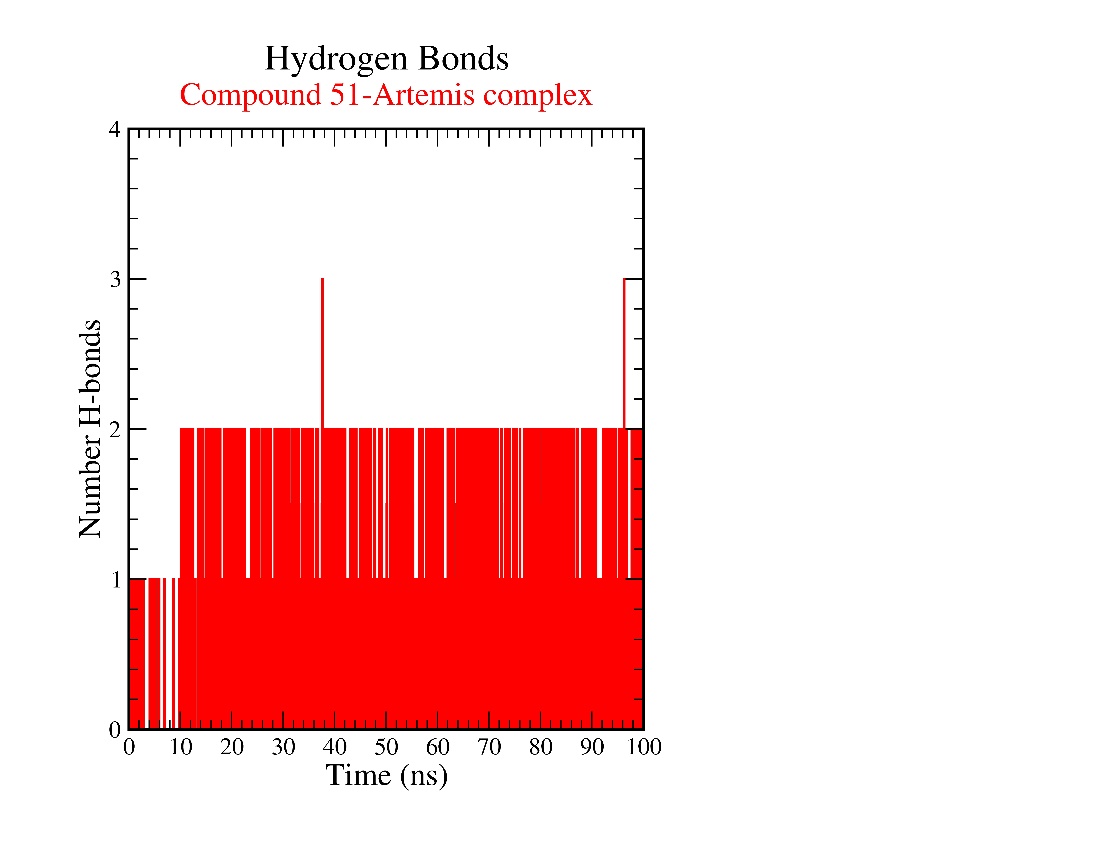

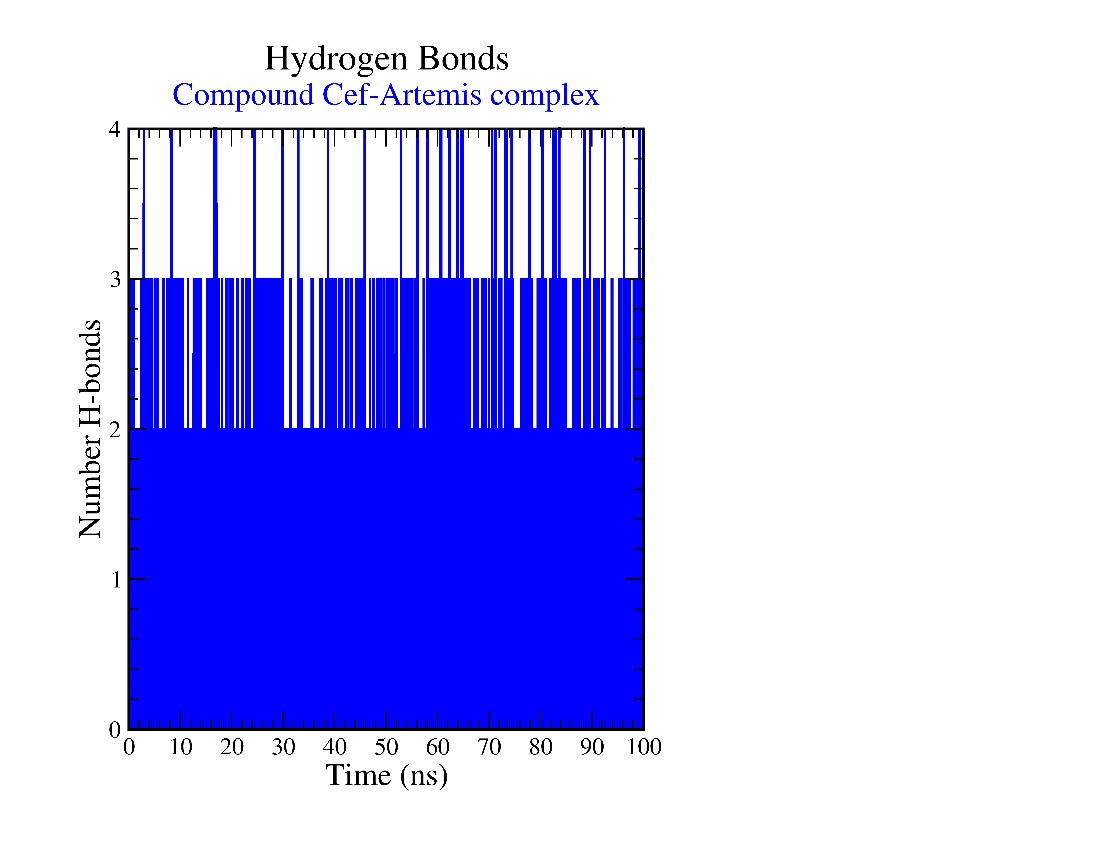


**Figure S12:** Graphical representation of the number of H-bond contacts formed by compounds 42, 51, and ceftriaxone in complex with Artemis protein.
